# The Dual Impact of Believing and Spreading Conspiracy Theories: Independent and Interactive Effects on Social Perceptions and Orientations

**DOI:** 10.1177/17470218251396952

**Published:** 2025-11-05

**Authors:** Aleksander B. Gundersen, Mikey Biddlestone, Jonas R. Kunst

**Affiliations:** 1 Department of Psychology, University of Oslo, Norway; 2 School of Psychology, University of Kent, Canterbury, England; 3 BI Norwegian Business School, Oslo, Norway

**Keywords:** conspiracy theory, dark triad, social distance, social perception, stereotype content

## Abstract

Prior research suggests that people who believe in and spread conspiracy theories are often viewed negatively, yet investigations systematically disentangling both factors are scarce. The present research addressed this gap through two pre-registered experiments with representative samples from the United States. In Study 1, 418 participants evaluated eight fictional individuals across 3,344 trials, presented as (a) believing in and/or (b) spreading conspiracy theories in a 2 × 2 within-subjects design. Analyses revealed that both characters who believed in conspiracy theories and those who spread them were perceived as less competent, moral, and warm, and as more narcissistic, Machiavellian, and psychopathic. Moreover, both believers and spreaders were perceived as likely to engage in conspiratorial actions themselves, and participants reported lower willingness to interact with them. However, significant interactions for all variables showed that these effects were particularly pronounced for characters who spread conspiracy theories without believing in them. Notably, participants’ own conspiracy beliefs and, to some extent, their right-wing political orientation attenuated several effects and reversed some. In Study 2, we employed the reverse-correlation technique to model 412 participants’ mental representations of individuals who varied in belief and/or spread of conspiracy theories using a 2 × 2 between-subjects design. Results were directionally consistent with Study 1—both believers and spreaders of conspiracy theories were mentally represented less favorably—but no interactions or moderations were observed. Moreover, believing had significantly stronger effects than spreading on the rating dimensions. We discuss the social implications of these results and outline future directions.

Conspiracy theories propose that events or phenomena result from the secret collaborations of powerful groups or individuals aiming to achieve nefarious goals that are harmful to the common good (Douglas et al., 2019; van der Linden et al., 2021). Notable examples include beliefs that 9/11 was an inside job, that John F. Kennedy’s (JFK) assassination was a Central Intelligence Agency operation, and that vaccines are embedded with microchips for surveillance and control. Some conspiracy beliefs are remarkably common. For example, post-2020 surveys revealed that one in five Americans believed the dangers of 5G technology were being covered up, over half the population questioned the official account of JFK’s assassination, and more than one-third suspected a covert group controls global events and rules the world (Uscinski, Enders, Klofstad, et al., 2022). While a few conspiracy theories—like the Watergate scandal or incidents of political and corporate misconduct—have been validated, the vast majority of those believed by the public throughout history have been false (Pipes, 1999).

A growing body of research has shed light on why people endorse conspiracy theories and their potential effects on individuals and society at large (for reviews, see Bowes et al., 2023; Douglas & Sutton, 2023; Douglas et al., 2019; Hornsey et al., 2023). Despite this, there is still much to learn about the social perceptions and interpersonal ramifications for those who endorse these theories (cf., Green et al., 2023; Toribio-Flórez et al., 2023, 2024). Our two pre-registered online experiments sought to address this gap, disentangling two key factors. In Study 1, we examined how participants perceived and judged others based on (a) their belief in, and (b) dissemination of, conspiracy theories. These individuals were evaluated across various dimensions (e.g., the Dark Triad; stereotypes), including participants’ willingness to have contact with them. Additionally, we investigated how these perceptions and contact willingness were shaped by the participants’ own conspiracy beliefs and political leanings. In Study 2, we investigated whether the perceptions and evaluations of individuals engaging with conspiracy theories extend beyond verbal descriptions, as studied in Study 1, to their mental/visual representation. Using the reverse correlation technique (Dotsch & Todorov, 2012), we modeled internal representations of faces of individuals who engage with conspiracy theories and examined how these representations differed based on whether the individuals believed (vs. did not believe) and/or spread (vs. did not spread) conspiracy theories.

## Social Perceptions of Conspiracy Believers

There is a prevailing notion that individuals who subscribe to conspiracy theories face negative judgments. The term “conspiracy theorist” often carries a derogatory connotation, implying a lack of personhood and competence among believers (Harambam & Aupers, 2017; Husting & Orr, 2007). Research by Green et al. (2023) supports this notion, showing that a hypothetical politician espousing conspiracy theories was viewed as less trustworthy, benevolent, predictable, and competent compared to an anti-conspiracy politician. Yet, to the best of our knowledge, no study has directly compared the social perceptions of ordinary conspiracy theory believers as directly compared to non-believers. Most critically, it is uncertain whether it is the mere belief in, the dissemination of, or a combination of both actions related to conspiracy theories that primarily affects these judgments.

While belief in conspiracy theories is relatively common (see, e.g., Uscinski, Enders, Klofstad, et al., 2022), much fewer individuals actively spread these theories (Douglas et al., 2019; Kunst, Gundersen, et al., 2024). Contrary to spreading indiscriminately from person-to-person (DeWitt et al., 2018), conspiracy theories are typically circulated within communities that already subscribe to them (Metaxas & Finn, 2017; Sunstein & Vermeule, 2009). Understanding the difference between merely believing in conspiracy theories and actively disseminating them is crucial for understanding how these individuals are perceived by others. Silent believers may be dismissed as naïve or stupid, but those who actively share these theories often face harsher judgments, being labeled as immoral or crazy (Uscinski et al., 2018). Despite this, there has been limited systematic research into how observers evaluate individuals based on their engagement with conspiracy theories, whether they believe them, spread them, or both.

### Stereotype Content

People commonly hold stereotypical beliefs about various social groups, including those who believe in conspiracy theories (Harambam & Aupers, 2017). These stereotypes are automatically activated upon encountering a group member (Devine, 1989), offering readily accessible information about the characteristics of typical group members which, in turn, shapes perceptions of them (Freeman & Ambady, 2011). The Stereotype Content Model (Cuddy et al., 2008; Fiske et al., 2002, 2007) offers a valuable lens for understanding how people view those who endorse and disseminate conspiracy theories. This model outlines two key dimensions of social perception—warmth and competence—that shape our judgments of individuals and groups. Warmth assesses perceived friendliness and likability, while competence gauges perceived skill, intelligence, and capability. These dimensions are crucial predictors of attitudes, emotions, and behaviors toward both groups and individuals, demonstrating their significance for intergroup and interpersonal relations (Cuddy et al., 2007, 2008).

In addition to warmth and competence, morality emerges as a critical additional dimension in evaluating groups, offering a more nuanced understanding of social attitudes. Morality, emphasizing sincerity and honesty, has been shown to be a potent predictor of attitudes toward groups, sometimes even more so than warmth and competence (Leach et al., 2007). Recent research underscores the complementary nature of these three dimensions—warmth, competence, and morality—in shaping our evaluations of groups and individuals. This triad collectively accounts for variations in how groups are perceived (Constantin & Cuadrado, 2021; Kunst et al., 2023). Interestingly, previous work has also demonstrated that groups perceived as cold and competent are viewed as more likely to conspire against one’s own ingroup (Winiewski et al., 2015).

In our studies, we tested how individuals are perceived based on their engagement with conspiracy theories, distinguishing among four distinct profiles in a 2 × 2 design: (1) those who spread, (2) those who believe, (3) those who do both, or (4) those who neither spread nor believe in conspiracy theories. Conspiracy theories are often viewed as false, illogical, and potentially harmful (Daniel & Harper, 2022), and those who engage with them can be seen in a negative light (Green et al., 2023; Klein et al., 2015). Accordingly, it seemed plausible that individuals who neither believe nor spread conspiracy theories might be perceived the most favorably. Meanwhile, numerous profit-driven operations have shown that conspiracy content can be monetized by leveraging sensational claims to attract advertising revenue, steering audiences into scams, and selling branded merchandise (Mahdhi & Wilmot, 2024; Robins-Early, 2022). Consequently, actors who openly disseminate conspiracy theories—sometimes referred to as “conspiracy entrepreneurs” (Sunstein & Vermeule, 2009)—could appear strategic or manipulative, possibly being criticized on moral grounds (Uscinski et al., 2018) and viewed as less warm (Cao et al., 2025). By contrast, those who believe conspiracy theories without disseminating them might be seen as misguided or less competent (Husting & Orr, 2007; Uscinski et al., 2018) but potentially less morally suspect than active spreaders. These silent believers may privately endorse conspiracy theories but refrain from actively spreading them, for example, due to concerns about social desirability or stigmatization. In such instances, perceivers may still infer belief indirectly (e.g., through subtle conversational cues or selective agreement) even when explicit spreading does not occur. Lastly, individuals who both believe and spread such theories could prompt more complex impressions, as they may be motivated by profit, ideological commitment, or social influence (Ren et al., 2023; Sunstein & Vermeule, 2009). We therefore anticipated that each combination of believing and spreading would yield distinct, if nuanced, perceptions regarding competence, morality, and warmth.

### Dark Personality Traits

Previous work has found links between the Dark Triad personality traits—narcissism, Machiavellianism, and psychopathy (Jonason & Webster, 2010)—and harboring conspiracy beliefs. Specifically, narcissism, defined by a sense of superiority, uniqueness, and entitlement, alongside sensitivity to perceived threats, has been linked to a propensity toward conspiracy theories (Cichocka et al., 2022; Stasielowicz, 2022). This link is partly due to narcissists’ desire for uniqueness, drawing them to conspiracy theories as sources of “exclusive” information that validates their need to feel special (Imhoff & Lamberty, 2017). Similarly, traits of psychopathy and Machiavellianism, characterized by callousness and manipulative behaviors, respectively, have been associated with a higher likelihood of endorsing conspiracy theories (Hughes & Machan, 2021; March & Springer, 2019; Uscinski, Enders, Diekman, et al., 2022). The manipulative and exploitative tendencies inherent to these dark traits may heighten individuals’ awareness of potential manipulation by authorities, as they might assume others would behave as they themselves would in positions of power—inclined to conspire (Douglas & Sutton, 2011; March & Springer, 2019). This perspective, in turn, may increase their inclination toward conspiracy beliefs. Yet, the degree to which individuals who believe in and/or disseminate conspiracy theories are also viewed as embodying narcissistic, Machiavellian, or psychopathic traits remains an open question.

Many conspiracy beliefs can have detrimental effects on both those who endorse them and the broader social environment (Daniel & Harper, 2022; Jolley et al., 2022). As such, individuals who believe or spread conspiracy theories may be perceived as exhibiting more dark personality traits than those who do not engage with such beliefs. In particular, spreading conspiracies without actually believing in them might come across as especially manipulative or antisocial, suggesting a potential link to traits like Machiavellianism or psychopathy. At the same time, conspiracy beliefs can fulfill a need for feeling special or unique (Cichocka et al., 2022; Kay, 2021), indicating that believing and actively spreading conspiracies could be associated with even higher perceptions of narcissism—especially when individuals are motivated to provoke reactions or display knowledge that sets them apart (Morosoli et al., 2022). Meanwhile, individuals who simply believe conspiracy theories—without disseminating them—might be seen as less overtly manipulative or attention-seeking but could still be perceived as more narcissistic, Machiavellian, and psychopathic than non-believers.

### Consequences for Social Orientations

Beyond affecting social perceptions, we posited that engaging with conspiracy theories—through belief and/or dissemination—would negatively impact interpersonal relationships. The stigma associated with believing in conspiracy theories (Harambam & Aupers, 2015) and the anticipation of negative judgment or social exclusion among those expressing such beliefs (Lantian et al., 2018) suggest that these individuals might be less favorably viewed and socially sanctioned. Indeed, conspiracy believers are more likely to experience social rejection (Biddlestone et al., 2025; Van Prooijen et al., 2023), and previous research has also found that people anticipate lower relationship satisfaction with strangers who endorse, rather than oppose, conspiracy theories in their online dating profiles (Toribio-Flórez et al., 2024). Therefore, we anticipated that participants would show a reduced willingness to have contact with individuals who believe in conspiracy theories. We also expected that participants would indicate the least willingness to have contact with individuals who are involved in spreading conspiracy theories—whether they believe in them or not—given that the disseminating behavior can be perceived as immoral and crazy (Uscinski et al., 2018).

### Are Those Who Engage With Conspiracy Theories Seen as Willing to Conspire?

Individuals who endorse conspiracy theories often attribute conspiratorial behavior to others, typically powerful elites (e.g., politicians, corporations). However, we sought to examine whether individuals who believe and/or spread conspiracy theories are themselves perceived as more likely to engage in conspiracies. While research on willingness to conspire is limited, Douglas and Sutton (2011) found that individuals were more likely to endorse conspiracy theories if they were personally willing to engage in conspiracies. Similarly, prior studies have shown that stronger conspiracy beliefs are associated with a greater tendency to act dishonestly (Alper et al., 2024) and to engage in criminal behavior (Jolley et al., 2019). Since dishonesty and criminal behavior are often integral to conspiratorial actions, we expect that individuals who believe in conspiracy theories, without spreading them, will be perceived as having greater intentions to conspire than those who neither believe nor spread conspiracy theories. However, perceptions of conspiratorial intentions may be strongest for individuals who spread conspiracy theories, particularly when they also believe in them, as this indicates a high level of conviction and a willingness to act upon it, despite the potential social costs this entails.

## The Moderating Role of Perceivers’ Conspiracy Beliefs and Political Orientation

While we expect individuals who engage with conspiracy theories to be generally evaluated unfavorably, characteristics of the perceiver may substantially influence these evaluations. Conservatives’ distrust of scientists, government officials, and mainstream media is thought to increase their susceptibility to conspiratorial thinking (van der Linden et al., 2021), and given this heightened conspiratorial mindset, they may evaluate others who engage with conspiracy theories more favorably. Specifically, research has shown that perceivers whose beliefs align with those endorsing conspiracy theories evaluate the latter more favorable. For example, Toribio-Flórez et al. (2024) demonstrated across several studies that participants’ own conspiracy beliefs moderated the relationship between others’ conspiracy beliefs and anticipated relationship satisfaction. This association was negative among perceivers with low conspiracy beliefs but showed signs of reversal among those with stronger conspiracy beliefs. Similarly, negative perceptions of a fictional politician espousing conspiracy theories were found to be less pronounced—even inverted—among individuals with right-wing political views or strong conspiracy beliefs (Green et al., 2023). Building on this, we anticipate similar findings in the current study: individuals who believe in and/or spread conspiracy beliefs are expected to be viewed generally more favorably by participants with strong conspiracy beliefs or a conservative/right-leaning political orientation. Furthermore, we expect that these participants will express greater willingness to have contact with such individuals.

## The Present Research

Prior work suggests that belief in, and expression of, conspiracy theories can lead to stigmatization (Harambam & Aupers, 2017; Lantian et al., 2018). However, the extent to which perceptions and evaluations vary—depending on whether an individual believes in, disseminates, both believes and disseminates, or does not engage with conspiracy theories—remains unexplored. The distinction between believing in and spreading conspiracy theories is crucial: while most conspiracy believers do not necessarily actively spread them, some misinformation and conspiracy-spreaders do not even care about or believe what they are spreading (Ren et al., 2023; Sinclair, 2024). Two pre-registered online experiments aimed to address this gap.

The first study aimed to test how individuals who varied experimentally in their engagement with conspiracy theories—whether they spread, believe, both, or neither—were perceived in terms of measures of stereotype content, dark personality traits, and intentions to conspire. Additionally, we assessed participants’ willingness to have contact with these individuals. In our pre-registered secondary analyses, we explored whether participants’ political orientation and personal conspiracy beliefs moderated the effects of spreading and believing in conspiracy theories on these perceptions and willingness for contact.

The second study, conducted in two steps, investigated whether the verbal descriptions from Study 1 extended to mental representations of individuals engaging with conspiracy theories. In the image generation step (Step 1), participants completed a reverse-correlation task, producing a single image per participant that approximated their imagined face of a person who believes (vs. does not believe) and/or spread (vs. does not spread) conspiracy theories. In the image rating step (Step 2), an independent sample, unaware of the hypotheses, evaluated these images using the same dimensions as Study 1. Additionally, guided by findings from Study 1, we examined the extent to which participants’ personal conspiracy beliefs and political orientation influenced the imagined facial images. In each study, we tested a set of pre-registered hypotheses. Please note that these differ slightly, as the hypotheses of Study 2 were developed based on results from Study 1. Both studies were oversampled to account for potential attrition due to failed attention checks, and Study 2 was oversampled more extensively, given the highly repetitive nature of the task.

Research on how individuals who engage with conspiracy theories are perceived remains limited. Existing studies have typically focused on either believers (e.g., Klein et al., 2015) or those who propagate such theories (e.g., Cao et al., 2025; Green et al., 2023), rather than examining both in tandem. The present research aimed to disentangle these two key factors by systematically investigating their independent and interactive effects on measures of social perception and orientation. Since perceptions of individuals and groups are known to shape attitudes, emotions, and behaviors toward them (Cuddy et al., 2007, 2008), understanding how different forms of engagement with conspiracy theories influence these perceptions and social consequences is crucial in an increasingly polarized society.

## Study 1

The first study examined how U.S. participants perceived individuals who differed in their engagement with conspiracy theories. The primary goal was to disentangle the effects of spreading and/or believing in conspiracy theories on social perceptions by testing both their separate and interactive effects. Using a 2 × 2 within-subjects design, participants evaluated eight fictional individuals who either believed (vs. did not believe) and/or spread (vs. did not spread) conspiracy theories. Participants rated these individuals on perceived competence, morality, warmth, Machiavellianism, narcissism, psychopathy, and intentions to conspire. Additionally, we assessed participants’ willingness to have contact with these individuals. We pre-registered all our hypotheses and the analysis plan on the Open Science Framework (OSF) at https://osf.io/tywk9?view_only=0bb0f7cd0855419787897862b074ef96.

We tested the following pre-registered hypothesis, predicting that different combinations of spread and belief in conspiracy theories would be ranked from highest to lowest on the following dimensions:

**H1:** Competence: neither believes nor spreads > spreads but does not believe > believes but does not spread > spreads and believes.
**H2:** Morality: neither believes nor spreads > spreads and believes = believes but does not spread > spreads but does not believe.
**H3:** Warmth: neither believes nor spreads > believes but does not spread > spreads but does not believe = spreads and believes.
**H4:** Machiavellianism: spreads but does not believe > spreads and believes > believes but does not spread > neither believes nor spreads.
**H5:** Narcissism: spreads and believes > spreads but does not believe > believes but does not spread > neither believes nor spreads.
**H6:** Psychopathy: spreads but does not believe > spreads and believes > believes but does not spread > neither believes nor spreads.
**H7:** Contact willingness^1^: neither believes nor spreads > believes but does not spread > spreads but does not believe = spreads and believes.
**H8:** Conspiracy intentions: spreads and believes > spreads but does not believe > believes but does not spread > neither believes nor spreads.

Several of the aforementioned perceived traits are known to influence social orientations, including willingness to engage with others who differ in the extent to which they exhibit these traits. For example, individuals perceived as both warm and competent are often admired, and the combination of high warmth and competence can facilitate behaviors such as helping, cooperation, and forming associations (Cuddy et al., 2007, 2008). In contrast, dark personality traits—particularly Machiavellianism and psychopathy—have been shown to negatively correlate with motivations to develop and maintain positive relationships with others (Jonason & Zeigler-Hill, 2018). Hence, we hypothesize that:

**H9:** Higher contact willingness is expected for fictional characters rated higher on competence and warmth, and lower on Machiavellianism, narcissism, and psychopathy, and conspiracy intentions.^2^

Since both political orientation and personal conspiracy beliefs can influence how individuals who engage with conspiracy theories are evaluated (Green et al., 2023; Toribio-Flórez et al., 2024), we examined the extent to which these factors moderated the effects of spreading and believing in conspiracy theories. Research suggests that individuals at both the far-left and far-right are more likely to support conspiracy theories than those with more moderate political views (Imhoff et al., 2022; Kunst, Gundersen, et al., 2024; but see, Enders et al., 2025, for new evidence contradicting this claim). Therefore, we tested whether political orientation moderated the effects of “spread” and “believe” in both linear and quadratic terms. Based on these considerations, we propose the following secondary hypotheses:

**HS1:** The separate effects of spreading and believing conspiracy theories on perceptions of individual differences and contact willingness will be reversed for participants scoring high on conspiracy beliefs and/or right-wing/conservative political orientation.^3^
**HS2:** The separate effects of spreading and believing conspiracy theories on perceptions of conspiracy intentions will be strengthened for participants scoring low on conspiracy beliefs but attenuated for those scoring high on conspiracy beliefs.

### Methods

We report how we determined our sample size, all data exclusions, all manipulations, and all measures in the study.

#### Participants

Using the lme4 (Bates et al., 2015) and simr (Green & MacLeod, 2016) packages in R, we conducted a pre-registered a priori power simulation. The results showed that enrolling 400 participants would provide over 90% power to detect an effect size of *d* = 0.15, with an α level of .05, at the level of a three-way interaction design incorporating eight trials per participant (*N*_trials_ = 3,200). Consequently, between June 15 and 19, 2023, we recruited 418 U.S. adults through the Prolific online survey platform. The sample comprised 200 men, 212 women, and 6 individuals who did not select a gender, all aged between 20 and 82 years (*M*_age_ = 44.61, *SD*_age_ = 14.19). This sample size afforded greater than 99% power to detect a three-way interaction effect size of *d* = 0.15 at an α level of .05. The sample closely mirrored the most recent U.S. political party affiliation distribution at the time of data collection (Republican: 29.7%, Democrat: 31.8%, Independent: 37.3%, other/no preference: 1.2%, as reported by the Gallup Organization, 2023). We pre-registered the inclusion of two attention checks, asking participants to respond in a certain way on a Likert-type scale (e.g., “It is important that you pay attention to this study. Please tick ‘Disagree’”); however, no participants failed both, so no exclusions were necessary. For further details on the sample demographics, refer to Table S1 in the Supplemental Materials (see link below).

#### Procedure and Materials

The Institutional Review Board of the department affiliated with the first and last authors approved this research (No. 26123962). All materials, code, and data for this study are available at https://osf.io/k34qm/.

Upon agreeing to participate in our survey, participants were first introduced to a definition of conspiracy theories from the Oxford English Dictionary (2023). This definition describes a conspiracy theory

. . . generally as a theory that an event or phenomenon occurs as a result of a conspiracy between interested parties, and specifically as a belief that some covert but influential agency (typically political in motivation and oppressive in intent) is responsible for an unexplained event.

We included this definition to establish a shared understanding among all participants of what “conspiracy theory” means in the context of our survey. The survey then consecutively presented participants with brief profiles of eight fictional characters: four with male names and four with female names. We randomly manipulated these characters’ (a) belief in and (b) dissemination of conspiracy theories, following a 2 (spread vs. does not spread) × 2 (believe vs. does not believe) format. For instance, one description would be: “Emma [believes/does not believe] in conspiracy theories [but/and] [spreads/does not spread] them.” Participants were asked after each trial to evaluate the character on the measures described next.

Participants rated each character on a scale from 1 (*not at all*) to 7 (*extremely*) based on several traits adopted from Leach et al. (2007). These traits included perceived *warmth* (i.e., 3 items: likeable, friendly, warm; α = .96), *competence* (i.e., 3 items: intelligent, competent, skilled; α = .96), and *morality* (i.e., 3 items: honest, sincere, trustworthy; α = .97). Additionally, participants assessed each character using a 12-item Dark Triad measure (Jonason & Webster, 2010), adapted to evaluate perceptions of the fictional characters’ dark triad traits. For instance, they rated the extent to which they perceived a character, like Emma, as *narcissistic* (4 items, e.g., “Emma would want others to admire them”; α = .93), *Machiavellian* (4 items, e.g., “Emma would manipulate others to get their way”; α = .98), or *psychopathic* (4 items, e.g., “Emma would lack remorse”; α = .94). These items were rated from 1 (*strongly disagree*) to 7 (*strongly agree*). The order of presenting perceived warmth, competence, morality, and the Dark Triad subscales was randomized.

Subsequently, we presented the participants with measures of *contact willingness* and *perceived conspiracy intentions*, in randomized order. The contact willingness toward each fictional character consisted of an adapted version of Bogardus (1933) social distance scale, and was gauged using 3 items (e.g., “How interested would you be in getting to know Emma?”; α = .90), with responses ranging from 1 (*very unlikely*) to 7 (*very likely*). The measure of conspiracy intentions utilized 4 items from the modified Generic Conspiracist Belief (GCB) Scale (Brotherton et al., 2013), tailored to assess intentions to conspire (e.g., “If Emma worked for the government, they would deliberately conceal information from the public”; α = .96). These items were rated on a scale from 1 (*strongly disagree*) to 7 (*strongly agree*).

Lastly, we assessed the participants’ own *beliefs in conspiracy theories* using the short form of the GCB Scale (Kay & Slovic, 2023). This involved 5 items (e.g., “The government allows or commits acts of terrorism on its own soil and disguises its involvement”; α = .89), rated from 1 (*strongly disagree*) to 7 (*strongly agree*). Following this, participants provided standard demographic information, including age, gender, ethnicity, education level, income, region of residence, and political orientation (ranging from 0—*very liberal/left-wing* to 10—*very conservative/right-wing*).

#### Analyses

In our analysis, we utilized multi-level modeling, where individual trials were nested within participants. This approach allowed us to examine both main effects and interactions, including up to a pre-registered three-way interaction: 2 (target spreads vs. does not spread) × 2 (target believes vs. does not believe) × perceiver political orientation/conspiracy beliefs. We conducted these analyses using IBM SPSS version 29 and R version 4.0.3 (R Core Team, 2020). For estimating the random effects models, we employed the lme4 (Bates et al., 2015) and lmerTest (Kuznetsova et al., 2017) packages. In these models, we set intercepts as random, and effects were also set to random, provided that the model converged successfully. Pairwise comparisons were tested with the emmeans (Lenth et al., 2021) package. Residual diagnostics for the multi-level models were tested with the DHARMa (Hartig, 2022) package, which evaluates whether model assumptions such as normality, homoscedasticity, independence of residuals, and potential overdispersion are met. Effects were visualized using the ggplot2 (Wickham, 2016) and interplot (Solt & Hu, 2015) packages. Deviating from the pre-registration, given the high number of tests, we decided to correct for Type 1 error inflation using Holm-corrected *p*-values to increase the robustness of our results.

### Results

The findings from our multi-level linear models, with various dependent variables, are detailed in Table 1. In each model, the intercepts for participants and trials, as well as the random effects for spreading and believing, were allowed to vary. Upon visually inspecting the residuals from each model, we observed that they followed a normal distribution. Additionally, dispersion tests across all models yielded non-significant results (*p* > .05). While residual outlier tests showed significance in three out of the eight models (*p* < .05), we opted to retain these residual outliers for a more conservative analytical approach, and as we did not pre-register excluding outliers. Residual diagnostics for the models are presented in Table S2 in the Supplemental Materials.

**Table 1.** Results of Multi-Level Linear Models: Examining the Fixed Effects of Spread and Believe, and Their Interaction on Dependent Variables, With Control for Random Intercepts and Effects.

Effect	Competence	Morality	Warmth	Machiavellianism	Narcissism	Psychopathy	Contact willingness	Conspiracy intentions
	*B* [95% CI]	*B* [95% CI]	*B* [95% CI]	*B* [95% CI]	*B* [95% CI]	*B* [95% CI]	*B* [95% CI]	*B* [95% CI]
Fixed effects								
(Intercept)	5.26*** [5.15, 5.37]	5.35*** [5.23, 5.46]	5.12*** [5.00, 5.24]	2.71*** [2.59, 2.83]	3.72*** [3.61, 3.84]	2.63*** [2.51, 2.75]	5.39*** [5.27, 5.50]	2.37*** [2.23, 2.50]
Spread	−1.75*** [−1.90, −1.60]	−3.00*** [−3.16, −2.84]	−2.04*** [−2.19, 1.90]	2.78*** [2.63, 2.94]	1.82*** [1.68, 1.97]	2.64*** [2.49, 2.79]	−2.73*** [−2.89, −2.57]	2.18*** [2.02, 2.34]
Believe	−0.84*** [−0.98, −0.69]	−0.81*** [−0.97, −0.66]	−0.46*** [−0.59, −0.34]	0.27*** [0.13, 0.42]	−0.03 [−0.15, 0.08]	0.49*** [0.35, 0.63]	−0.86*** [−1.01, −0.70]	0.40*** [0.24, 0.56]
Spread × Believe	0.91*** [0.80, 1.01]	1.97*** [1.84, 2.09]	0.97*** [0.86, 1.07]	−1.25*** [−1.38, −1.12]	−0.44*** [−0.55, −0.32]	−1.31*** [−1.43, −1.18]	1.42*** [1.31, 1.53]	−1.31*** [−1.45, −1.18]
Random effects	Variance (*SD*)	Variance (*SD*)	Variance (*SD*)	Variance (*SD*)	Variance (*SD*)	Variance (*SD*)	Variance (*SD*)	Variance (*SD*)
Participants intercept	0.95 (0.98)	1.07 (1.04)	1.08 (1.04)	1.12 (1.06)	1.12 (1.06)	1.00 (1.00)	0.98 (0.94)	1.44 (1.20)
Spread	1.71 (1.31)	2.00 (1.42)	1.73 (1.31)	1.78 (1.34)	1.60 (1.27)	1.66 (1.29)	1.99 (1.41)	1.79 (1.34)
Believe	1.70 (1.30)	1.86 (1.36)	1.15 (1.07)	1.43 (1.19)	0.76 (0.87)	1.29 (1.14)	1.94 (1.39)	2.03 (1.42)
Trial	0.00 (0.04)	0.00 (0.03)	0.00 (0.06)	0.00 (0.02)	0.00 (0.00)	0.00 (0.06)	0.00 (0.06)	0.01 (0.06)
*R* ^2^								
Conditional	.85	.86	.86	.81	.79	.81	.88	.81
Marginal	.19	.35	.26	.39	.29	.37	.34	.22

*Note. SE*s, *t*-values, and degrees of freedom for all analyses are reported in Tables S3 to S5 in the Supplemental Materials. CI = confidence interval; *SE* = standard error.

*** *p*_Holm_ < .001.

In each of our models, corresponding to the various dependent variables, the fixed effects analysis showed that the interactions between the factors “spread” and “believe” significantly predicted (*p*_Holm_ < .001) perceptions of the fictional characters in terms of competence (H1), morality (H2), warmth (H3), Machiavellianism (H4), narcissism (H5), psychopathy (H6), willingness to contact (H7), and conspiracy intentions (H8).

**Figure 1.**
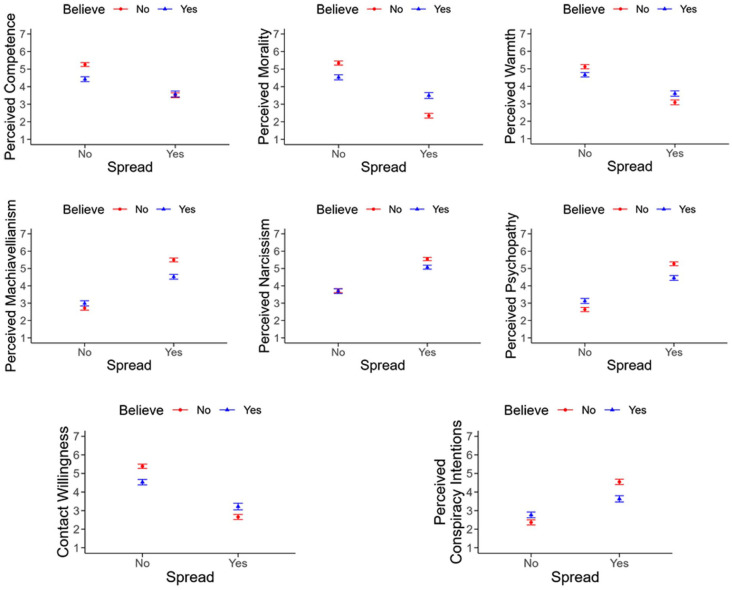
Estimated marginal means of all combinations of spread and believe on individual differences, contact willingness, and conspiracy intentions. *Note*. Error bars represent 95% CIs of estimated mean values. CI = confidence interval.

To examine our hypotheses (H1–H8), we conducted follow-up pairwise comparisons across the six possible comparisons between the four means resulting from the 2 spreading (yes/no) × 2 believing (yes/no) design for all our models. The findings are illustrated in Figure 1, with comprehensive statistics and effect sizes available in the Supplemental Materials, Section S1. Focusing on perceived competence (H1) as the dependent variable, our comparisons showed significant differences (*ps*_Holm_ < .001) across all six combinations of spread versus believe, with one exception: the comparison between characters who only spread conspiracy theories and those who both spread and believe did not yield a significant difference (*p*_Holm_ = .206). Partially supporting our hypothesis, the order of perceived competence among the fictional characters, from highest to lowest, was as follows: those who neither believe nor spread > those who believe but do not spread > those who both spread and believe and those who spread but do not believe.

In assessing perceived morality (H2), the results revealed significant differences (all *ps*_Holm_ < .001) between all combinations of spreading and believing. Characters perceived as most moral followed this order: those who neither believe nor spread > those who believe but do not spread > those who spread and believe > those who spread but do not believe.

When evaluating perceived warmth (H3), our analysis showed significant differences (all *ps*_Holm_ < .001) across all spread versus belief combinations. The characters perceived as warmest were ranked as follows: those who neither believe nor spread > those who believe but do not spread > those who spread and believe > those who spread but do not believe.

In the analysis of the Dark Triad traits, specifically focusing on Machiavellianism (H4), the pairwise comparisons revealed significant differences (all *ps*_Holm_ < .001) among all combinations of spreading and believing. The ranking of characters perceived as most Machiavellian was as follows: those who spread but do not believe > those who spread and believe > those who believe but do not spread > those who neither believe nor spread.

Regarding perceived narcissism (H5), there were significant differences (all *ps*_Holm_ < .001) between five of the six mean comparisons of spreading and believing. There was no significant difference (*p*_Holm_ = .586) between characters who neither spread nor believed and those who only believed. Thus, partially in line with our hypothesis, the characters perceived as most narcissistic were ranked in the following order: those who spread but do not believe > those who spread and believe > those who neither believe nor spread and those who believe but do not spread.

In assessing perceived psychopathy (H6), the analysis showed that all combinations of spreading and believing were significantly different from each other (all *ps*_Holm_ < .001). The characters perceived as most psychopathic ranked in the following order: those who spread but do not believe > those who spread and believe > those who believe but do not spread > those who neither believe nor spread.

Regarding willingness to have contact (H7), significant differences (all *ps*_Holm_ < .001) were observed among all spread versus believe comparisons. Participants were most willing to have contact with characters in this order: those who neither believe nor spread > those who believe but do not spread > those who spread and believe > those who spread but do not believe.

Finally, regarding perceived intentions to conspire (H8), the results showed significant differences (all *ps*_Holm_ < .001) across all combinations of spreading and believing. The characters perceived as having the highest intentions to conspire were ranked as follows: those who spread but do not believe > those who spread and believe > those who believe but do not spread > those who neither believe nor spread.

Additionally, for purposes of comparison with Study 2 presented later on, we ran another set of multi-level linear models to examine whether the main effects of spread and belief differed significantly across each rating dimension. Both variables were effect-coded to facilitate the interpretation of the main effects in the presence of interactions. We estimated a bootstrapped 95% confidence interval (CI; *N*_sim_ = 1,000) for the difference between the two main effects. Results indicated that spreading had a significantly stronger effect than believing on perceived competence, ∆*B* = −0.91, 95% CI [−0.98, −0.86], morality, ∆*B* = −2.19, 95% CI [−2.26, −2.12], warmth, ∆*B* = −1.58, 95% CI [−1.64, −1.52], Machiavellianism, ∆*B* = 2.51, 95% CI [2.44, 2.59], narcissism, ∆*B* = 1.86, 95% CI [1.80, 1.92], psychopathy, ∆*B* = 2.15, 95% CI [2.08, 2.22], conspiracy intentions, ∆*B* = 1.78, 95% CI [1.68, 1.84], and willingness for contact, ∆*B* = −1.87, 95% CI [−1.92, −1.80]. The results from the multi-level models are presented in Tables S6 to S8 in the Supplemental Materials.

To examine the relationships between competence, morality, warmth, Machiavellianism, narcissism, psychopathy, and conspiracy intentions with willingness to contact (H9 and H10), we employed another multi-level linear model. To assess whether these variables predicted contact willingness beyond the effects of the experimental manipulation, the “spread” and “believe” factors were included as covariates in the model. Consistent with our previous models, we allowed the intercepts for participants and trials and the random effects for spreading and believing to vary. However, we included the continuous predictors solely as fixed effects. This decision was made because including them as random effects would result in a higher number of random effects (4,180) than observations (3,344). Upon visually inspecting the residuals, we noted a normal distribution. Both the dispersion test (*p* = .768) and the outlier test (*p* = .078) yielded non-significant results. The detailed outcomes of this model are presented in Table 2.

**Table 2.** Results of Multi-Level Linear Models: Testing Effects of Individual Difference Traits on Contact Willingness With the Fictional Characters.

Effect		95% CI					
	*B*	*LL*	*UL*	*SE*	*t*	*df*	*p*_Holm_
Fixed effects							
(Intercept)	3.31	3.06	3.56	.13	25.85	2,327.92	< .001
Spread	−0.63	−0.75	−0.51	.07	−10.19	1,190.57	<.001
Believe	−0.35	−0.44	−0.27	.05	−7.83	698.91	<.001
Competence	0.13	0.09	0.16	.02	7.15	2,900.25	< .001
Warmth	0.18	0.14	0.22	.02	8.84	3,165.47	<.001
Morality	0.24	0.20	0.27	.02	13.43	3,219.64	<.001
Machiavellianism	−0.11	−0.14	−0.08	.02	−6.61	2,943.81	<.001
Narcissism	0.00	−0.03	0.03	.01	0.16	3,002.96	.875
Psychopathy	−0.07	−0.10	−0.03	.02	−3.94	3,057.41	<.001
Conspiracy intentions	−0.14	−0.17	−0.12	.01	−11.02	3,209.90	<.001
Spread × Believe	0.24	0.14	0.34	.05	4.77	3,057.86	<.001
Random effects	Variance (*SD*)						
Participants intercept	0.28 (0.53)						
Spread	0.56 (0.75)						
Believe	0.41 (0.64)						
Trial	0.00 (0.03)						
*R* ^2^							
Conditional	.91						
Marginal	.71						

*Note.* CI = confidence interval; *LL* = lower limit; *UL* = upper limit; *SE* = standard error; *df* = degree of freedom.

Consistent with our hypothesis, our findings revealed that higher ratings of perceived competence, morality, and warmth were significantly associated with higher willingness to have contact with the fictional characters. On the other hand, lower ratings of perceived Machiavellianism, psychopathy, and intentions to conspire were significantly associated with a decreased willingness to have contact with these characters. However, no statistically significant relationship was observed between ratings of narcissism and willingness to have contact with the fictional characters. Notably, the pattern of results remained consistent when “spread” and believe” were excluded from the model, with the exception that perceived narcissism became significantly associated with contact willingness (see Table S9 in the Supplemental Materials).

#### Secondary Hypotheses

We tested the secondary hypothesis that the separate effects of spreading and believing in conspiracy theories on perceived individual differences (i.e., perceived competence, morality, warmth, Machiavellianism, narcissism, psychopathy, and conspiracy intentions) and willingness to have contact with the fictional characters would differ depending on participants’ levels of conspiracy beliefs or their political orientation. Specifically, we expected that these negative effects would be reversed for participants with high conspiracy beliefs or a right-wing political leaning. Additionally, we anticipated that the positive effects of spreading and belief on perceptions of conspiracy intentions would increase for those with low conspiracy beliefs but diminish for those with high conspiracy beliefs. To evaluate these hypotheses, we employed another set of multi-level linear models for each trait. Specifically, we examined two-way interactions between “belief” and political orientation/conspiracy beliefs, as well as between “spread” and political orientation/conspiracy beliefs. Political orientation was modeled as both a linear effect and a quadratic effect to capture potential nonlinear relationships. Similar to our previous models, we allowed variations in the intercepts for participants and trials, as well as in the random effects for spread and belief. Residual diagnostics of the models indicated that all dispersion tests were non-significant (*p* > .05), while three out of the eight residual outlier tests showed significance (*p* < .05; see Table S10 in the Supplemental Materials). The residual outliers were retained for a more conservative analytical approach. The outcomes of the models considering participants’ conspiracy beliefs and political orientation are detailed in Table 3.

**Table 3.** Results of Multi-Level Linear Models: Investigating Separate Two-Way Interactions of Spread and Believe With Participants’ Conspiracy Beliefs and Political Orientation.

Effect	Competence		Morality		Warmth		Machiavellianism		Narcissism		Psychopathy		Contact willingness		Conspiracy intentions	
	*B* [95% CI]	*p*	*B* [95% CI]	*p*	*B* [95% CI]	*p*	*B* [95% CI]	*p*	*B* [95% CI]	*p*	*B* [95% CI]	*p*	*B* [95% CI]	*p*	*B* [95% CI]	*p*
Fixed effects																
(Intercept)	5.40 [5.12, 5.69]	<.001	5.24 [4.92, 5.56]	<.001	5.04 [4.74, 5.34]	<.001	2.19 [1.88, 2.49]	<.001	3.39 [3.09, 3.69]	<.001	2.11 [1.81, 2.40]	<.001	5.53 [5.25, 5.81]	<.001	1.49 [1.16, 1.82]	<.001
Spread	−1.88 [−2.25, −1.50]	<.001	−3.08 [−3.49, −2.66]	<.001	−2.16 [−2.54, −1.79]	<.001	3.11 [2.72, 3.49]	<.001	2.31 [1.96, 2.67]	<.001	3.14 [2.77, 3.51]	<.001	−3.11 [−3.50, −2.71]	<.001	2.31 [1.92, 2.71]	<.001
Believe	−1.94 [−2.28, −1.60]	<.001	−1.12 [−1.51, −0.72]	<.001	−0.92 [−1.23, −0.62]	<.001	0.59 [0.23, 0.95]	.011	0.26 [−0.02, 0.53]	.394	0.73 [0.38, 1.08]	<.001	−1.96 [−2.33, −1.60]	<.001	0.86 [0.44, 1.27]	<.001
GCB	−0.16 [−0.22, −0.09]	<.001	−0.17 [−0.24, −0.09]	<.001	−0.14 [−0.21, −0.06]	.002	0.25 [0.18, 0.32]	<.001	0.19 [0.12, 0.26]	<.001	0.25 [0.18, 0.31]	<.001	−0.18 [−0.25, −0.12]	<.001	0.37 [0.29, 0.44]	<.001
Political orientation	0.03 [−0.00, 0.07]	.205	0.04 [−0.00, 0.07]	.275	0.06 [0.02, 0.09]	.016	−0.01 [−0.05, 0.02]	>.999	−0.04 [−0.08, −0.00]	.210	−0.00 [−0.04, 0.03]	>.999	0.03 [−0.01, 0.08]	.497	−0.02 [−0.06, 0.02]	>.999
Political orientation^2^	8.61 [2.65, 14.57]	.025	6.86 [0.17, 13.54]	.227	9.21 [2.95, 15.48]	.021	−5.07 [−11.38, 1.25]	.583	3.68 [−2.60, 9.97]	.973	−3.63 [−9.72, 2.47]	>.999	7.23 [1.45, 13.01]	.073	−4.40 [−11.18, 2.37]	>.999
GCB × Spread	**0.15** [0.06, 0.24]	**.005**	**0.26** [0.16, 0.36]	**<.001**	**0.15** [0.06, 0.24]	**.005**	**−0.25** [−0.34, −0.16]	**<.001**	**−0.24** [−0.32, −0.16]	**<.001**	**−0.28** [−0.37, −0.19]	**<.001**	**0.25** [0.16, 0.35]	**<.001**	**−0.25** [−0.34, −0.16]	**<.001**
GCB × Believe	**0.32** [0.24, 0.40]	**<.001**	**0.25** [0.16, 0.34]	**<.001**	**0.23** [0.16, 0.30]	**<.001**	**−0.16** [−0.24, −0.07]	**.003**	**−0.10** [−0.16, −0.03]	**.029**	**−0.15** [−0.23, −0.07]	**.002**	**0.35** [0.27, 0.44]	**<.001**	**−0.22** [−0.32, −0.12]	**<.001**
Political Orientation × Spread	0.01 [−0.03, 0.05]	>.999	0.03 [−0.02, 0.08]	.810	0.01 [−0.03, 0.06]	>.999	−0.01 [−0.06, 0.03]	>.999	0.03 [−0.02, 0.07]	.973	−0.03 [−0.07, 0.02]	>.999	0.03 [−0.01, 0.08]	.497	0.02 [−0.03, 0.06]	>.999
Political Orientation × Believe	**0.07** [0.03, 0.11]	**.008**	0.06 [0.02, 0.11]	.051	0.02 [−0.02, 0.06]	>.999	−**0.06** [−0.11, −0.02]	**.023**	−0.03 [−0.06, 0.00]	.439	−**0.06** [−0.10, −0.02]	**.028**	**0.09** [0.05, 0.14]	<**.001**	−0.05 [−0.10, −0.00]	.196
Political Orientation^2^ × Spread	−2.86 [−10.67, 4.94]	>.999	−1.88 [−10.51, 6.75]	>.999	−0.66 [−8.47, 7.14]	>.999	3.59 [−4.47, 11.66]	>.999	−1.22 [−8.70, 6.25]	.973	3.66 [−3.98, 11.30]	>.999	−1.50 [−9.71, 6.71]	.720	2.96 [−5.30, 11.21]	>.999
Political Orientation^2^ × Believe	−2.25 [−9.36, 4.85]	>.999	−0.83 [−9.12, 7.45]	>.999	−3.31 [−9.73, 3.12]	>.999	3.18 [−4.41, 10.76]	>.999	2.69 [−3.04, 8.42]	.937	1.38 [−5.92, 8.69]	>.999	−5.42 [−13.04, 2.20]	.497	3.82 [−4.86, 12.49]	>.999
Random effects	Variance (*SD*)		Variance (*SD*)		Variance (*SD*)		Variance (*SD*)		Variance (*SD*)		Variance (*SD*)		Variance (*SD*)		Variance (*SD*)	
Participants intercept	0.93 (0.97)		1.10 (1.05)		1.06 (1.03)		0.95 (0.98)		1.03 (1.02)		0.89 (0.94)		0.83 (0.91)		1.14 (1.07)	
Spread	1.66 (1.29)		1.93 (1.39)		1.67 (1.29)		1.65 (1.28)		1.48 (1.22)		1.46 (1.21)		1.83 (1.35)		1.72 (1.31)	
Believe	1.33 (1.15)		1.74 (1.32)		1.04 (1.02)		1.40 (1.18)		0.73 (0.86)		1.30 (1.14)		1.53 (1.24)		1.96 (1.40)	
Trial	0.00 (0.04)		0.00 (0.04)		0.00 (0.06)		0.00 (0.03)		0.00 (0.00)		0.00 (0.07)		0.00 (0.06)		0.01 (0.07)	
*R* ^2^																
Conditional	.84		.80		.84		.79		.78		.78		.85		.78	
Marginal	.24		.34		.29		.39		.31		.37		.40		.22	

*Note.* Holm-corrected *p*-values are presented. Significant two-way interactions are presented in bold. *SE*s, *t*-values, and degrees of freedom for all analyses are reported in Tables S11 to S13 in the Supplemental Materials. CI = confidence interval. GCB = Generic Conspiracist Beliefs; *SE* = standard error.

#### Two-Way Interactions With Participants’ Conspiracy Beliefs

**Figure 2.**
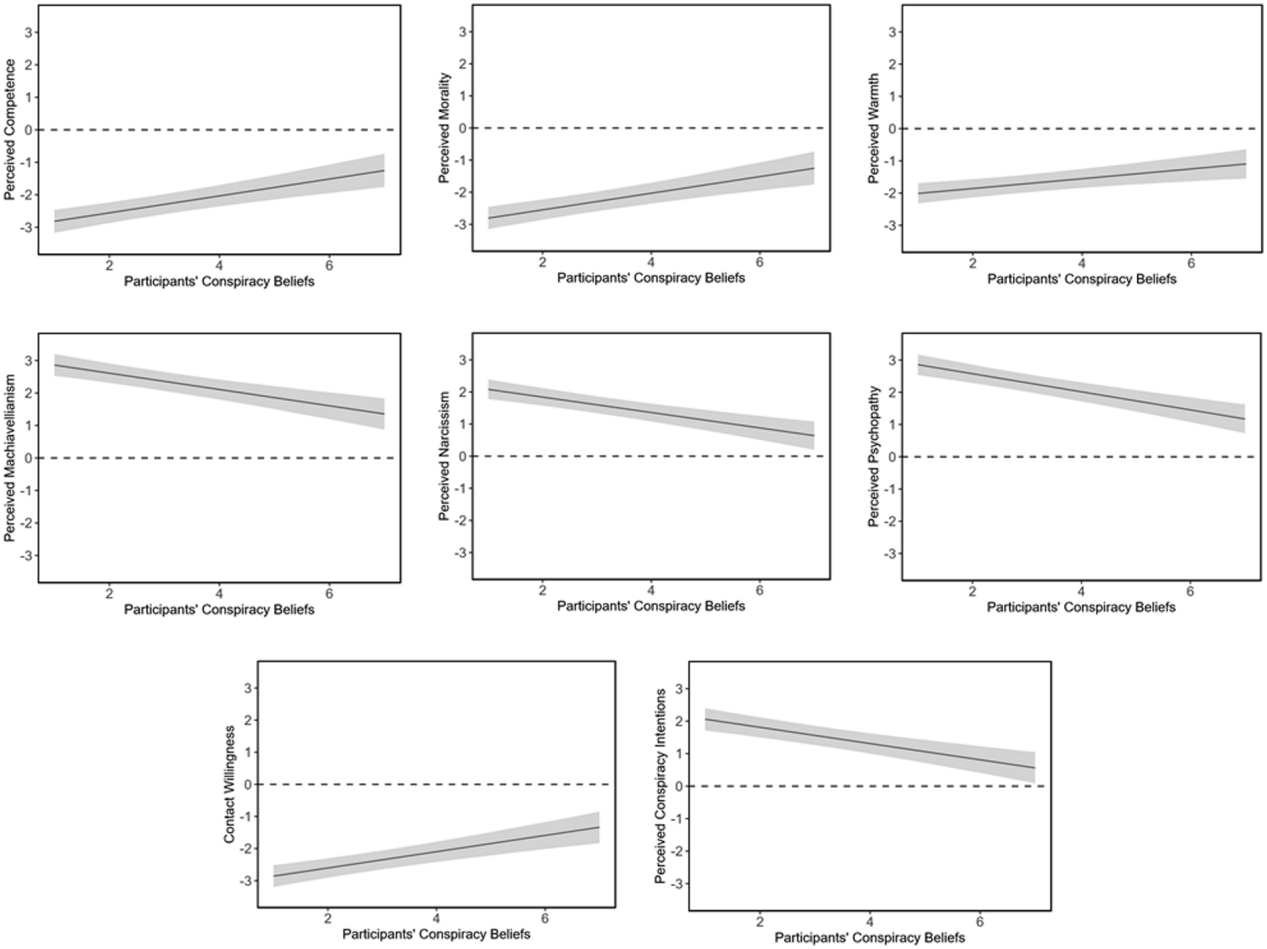
The effect (*B*) of spreading conspiracy theories on dependent variables across different levels of participants’ conspiracy beliefs. *Note*. Effects are unstandardized regression coefficients (*B*). Ribbons represent 95% CIs. CI = confidence interval.

All two-way interactions between the factors “spread” and “believe” and the participants’ conspiracy beliefs were significant (*ps*_Holm_ < .05; see Table 3). Across all models, we observed that the influence of the “spread” factor was significantly attenuated, yet not reversed, the higher participants scored on the GCB Scale, as visualized in Figure 2.

As an example, whereas the effect of spreading conspiracy theories on perceived competence was markedly negative for participants with low conspiracy beliefs, it got weaker and closer to zero for participants with high conspiracy beliefs, while still exerting a significant negative effect. The same pattern was observed for all remaining variables.

**Figure 3.**
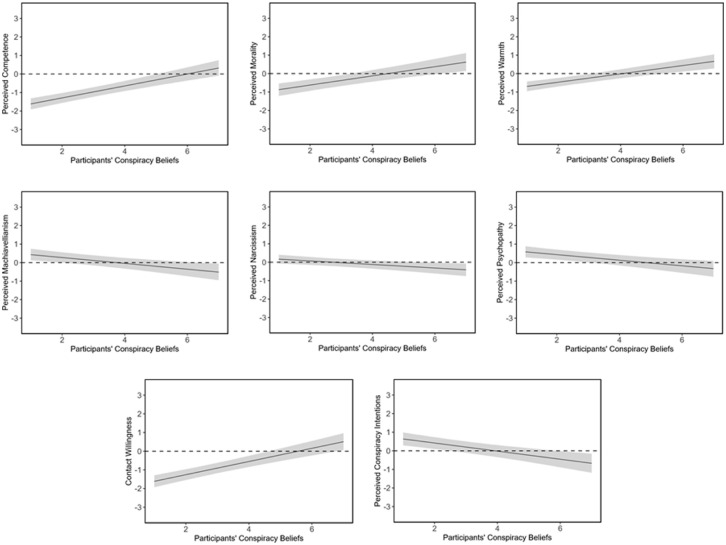
The effect (*B*) of believing in conspiracy theories on dependent variables across different levels of participants’ conspiracy beliefs. *Note*. Effects are unstandardized regression coefficients (*B*). Ribbons represent 95% CIs. CI = confidence interval.

By contrast, as visualized in Figure 3, the impact of “belief” on most traits was strongly attenuated and often reversed for participants with high versus low conspiracy beliefs. Whereas participants with low conspiracy beliefs evaluated the fictional characters who believed in conspiracy theories as less moral and warm, these characters were evaluated more positively on these dimensions by participants with high conspiracy beliefs. Regarding perceived competence, participants with low conspiracy beliefs rated the believing characters as less competent, whereas the effect of belief was close to zero for participants’ scored high on conspiracy beliefs. In terms of the dark triad, the same tendency was observed for Machiavellianism. Low conspiracy believers evaluated the believing character as more Machiavellian, whereas high conspiracy believers evaluated the character as less Machiavellian. However, for narcissism, believing characters were rated as lower (i.e., more favorably) by participants with high conspiracy beliefs, whereas the effect was close to zero for participants with low conspiracy beliefs. For perceived psychopathy, believing characters were rated as more psychopathic by participants with low conspiracy beliefs, while the effect was close to zero for participants with high conspiracy beliefs. Finally, whereas participants with low conspiracy beliefs indicated lower contact willingness and perceived higher intentions to conspire, the association was the opposite for participants with high conspiracy beliefs.

#### Two-Way Interactions With Participants’ Political Orientation

The results of the models that took into account participants’ political orientation are elaborated in Table 3. In these models, we observed significant two-way interactions between the “belief” factor and participants’ political orientation, specifically for perceived competence, Machiavellianism, psychopathy, and willingness to contact. No statistically significant interactions between the “spread” factor and participants’ political orientation were observed, nor between the quadratic effect of political orientation and the “spread” and “believe” factors.

**Figure 4.**
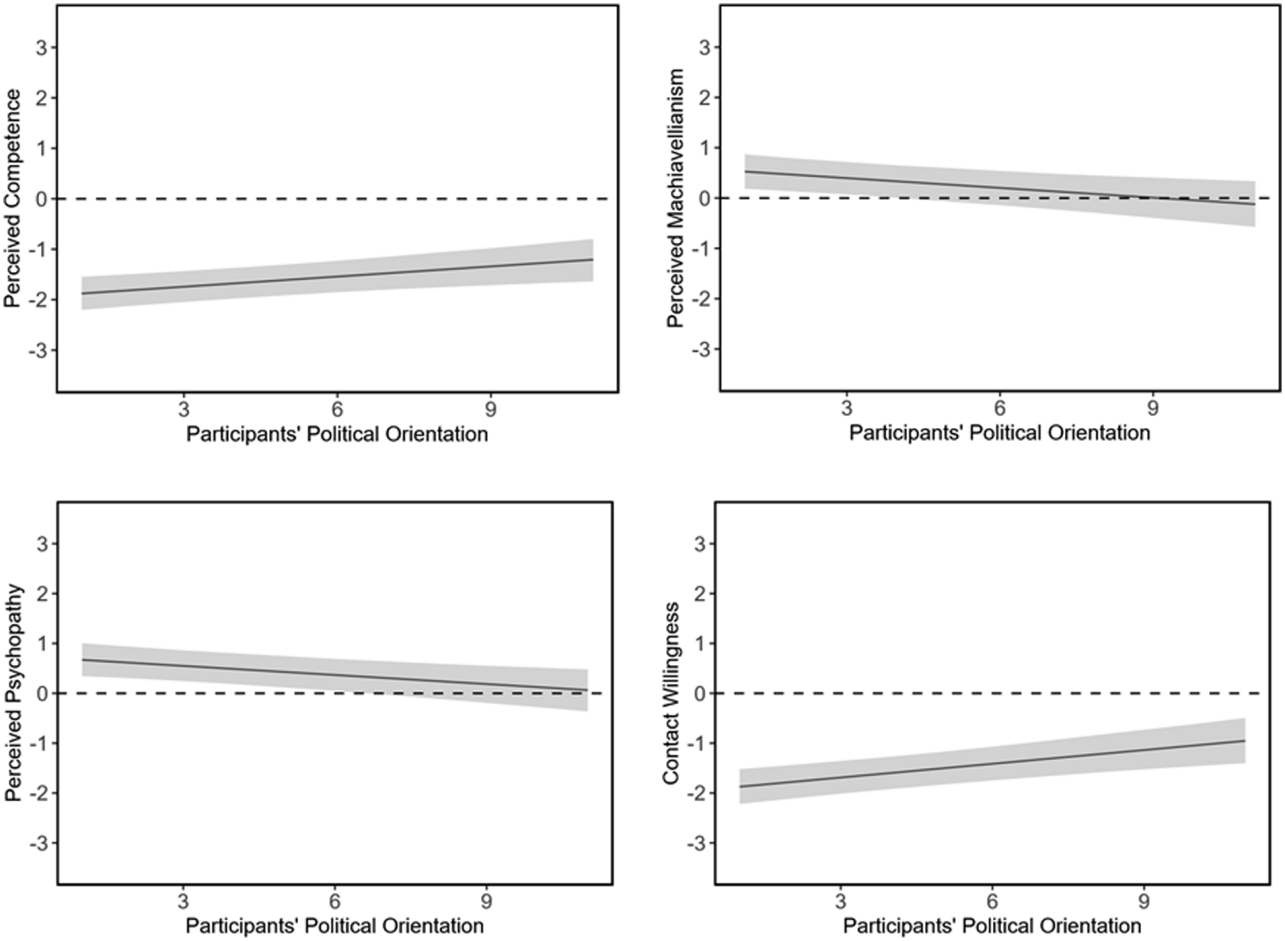
The effect (*B*) of believing in conspiracy theories on dependent variables across different levels of participants’ political orientation. *Note*. Effects are unstandardized regression coefficients (*B*). Ribbons represent 95% CIs. Higher values on the *x*-axis indicate a more right-leaning political orientation. CI = confidence interval.

As detailed in Figure 4, the impact of belief on the perceived competence of the fictional characters was more negative for participants on the political left, and this effect was weaker for participants further right on the political spectrum they were. In terms of perceived Machiavellianism and psychopathy, we observed that participants on the far left evaluated the believing characters as more Machiavellian and psychopathic, whereas this effect approached zero for participants on the political right. Finally, whereas participants on the far-left indicated lower contact willingness with the believing characters, this negative effect was reduced for more right-leaning participants.

#### Three-Way Interactions

Finally, to determine if participants’ conspiracy beliefs and political orientation moderated the interactive effects of “spread” and “believe” on the individual-difference variables, we conducted the same models as before but included three-way interactions. These interactions involved “spread,” “belief,” and either participants’ conspiracy beliefs or their political orientation (included in the same model). We again included both the linear term and the quadratic term of political orientation. In these models, we allowed the intercepts for participants and trials to vary, as well as the random effects of “spread” and “belief.” All dispersion tests were non-significant (*p* > .05). However, residual outlier tests showed significance (*p* < .05) in two out of the eight models (see Table S14 in the Supplemental Materials). Similar to our previous models, we chose to retain the residual outliers in our analysis for a more conservative approach, and as no outlier exclusions were pre-registered. The model results are displayed in Table 4.

**Table 4.** Results of Multi-Level Linear Models: Investigating Three-Way Interactions of Spread and Believe With Participants’ Conspiracy Beliefs and Political Orientation.

Effect	Competence		Morality		Warmth		Machiavellianism		Narcissism		Psychopathy		Contact willingness		Conspiracy intentions	
	*B* [95% CI]	*p*	*B* [95% CI]	*p*	*B* [95% CI]	*p*	*B* [95% CI]	*p*	*B* [95% CI]	*p*	*B* [95% CI]	*p*	*B* [95% CI]	*p*	*B* [95% CI]	*p*
Fixed effects																
(Intercept)	5.72 [5.43, 6.01]	<.001	5.91 [5.60, 6.23]	<.001	5.38 [5.08, 5.69]	<.001	1.86 [1.54, 2.17]	<.001	3.31 [3.00, 3.62]	<.001	1.71 [1.41, 2.01]	<.001	6.04 [5.75, 6.32]	<.001	1.22 [0.88, 1.56]	<.001
Spread	−2.55 [−2.95, −2.15]	<.001	−4.46 [−4.90, −4.03]	<.001	−2.87 [−3.28, −2.47]	<.001	3.78 [3.36, 4.21]	<.001	2.48 [2.09, 2.88]	<.001	3.94 [3.54, 4.34]	<.001	−4.15 [−4.57, −3.74]	<.001	2.88 [2.45, 3.31]	<.001
Believe	−2.55 [−2.92, −2.18]	<.001	−2.39 [−2.80, −1.98]	<.001	−1.57 [−1.90, −1.24]	<.001	1.23 [0.83, 1.62]	<.001	0.41 [0.09, 0.73]	.138	1.50 [1.13, 1.88]	<.001	−2.92 [−3.31, −2.54]	<.001	1.36 [0.92, 1.81]	<.001
GCB	−0.17 [−0.24, −0.10]	<.001	−0.20 [−0.27, −0.12]	<.001	−0.15 [−0.22, −0.08]	<.001	0.26 [0.18, 0.33]	<.001	0.16 [0.09, 0.24]	<.001	0.26 [0.19, 0.33]	<.001	−0.20 [−0.26, −0.13]	<.001	0.35 [0.27, 0.42]	<.001
Political orientation	0.03 [−0.01, 0.06]	.595	0.02 [−0.01, 0.06]	.583	0.05 [0.01, 0.08]	.075	−0.01 [−0.05, 0.03]	>.999	−0.03 [−0.07, 0.01]	.758	−0.00 [−0.04, 0.03]	>.999	0.01 [−0.02, 0.04]	.549	−0.02 [−0.06, 0.02]	>.999
Political orientation^2^	9.50 [3.51, 15.59]	.016	8.10 [1.58, 14.61]	.084	10.95 [4.64, 17.26]	.007	−4.50 [−11.04, 2.04]	>.999	5.03 [−1.48, 11.53]	.782	−4.44 [−10.64, 1.76]	.862	9.27 [3.39, 15.15]	.013	−6.13 [−13.08, 0.83]	.595
GCB × Spread	0.17 [0.08, 0.27]	.003	0.31 [0.21, 0.42]	<.001	0.18 [0.08, 0.27]	.002	−0.25 [−0.35, −0.15]	<.001	−0.19 [−0.28, −0.10]	<.001	−0.32 [−0.41, −0.22]	<.001	0.28 [0.19, 0.38]	<.001	−0.21 [−0.31, −0.11]	<.001
GCB × Believe	0.35 [0.26, 0.43]	<.001	0.30 [0.21, 0.40]	<.001	0.25 [0.18, 0.33]	<.001	−0.16 [−0.25, −0.07]	.008	−0.05 [−0.12, 0.03]	.877	−0.19 [−0.28, −0.10]	<.001	0.38 [0.29, 0.47]	<.001	−0.18 [−0.28, −0.07]	.009
Political Orientation × Spread	0.03 [−0.02, 0.08]	.620	0.06 [0.01, 0.11]	.103	0.03 [−0.01, 0.08]	.323	−0.02 [−0.07, 0.03]	>.999	0.00 [−0.04, 0.05]	>.999	−0.03 [−0.08, 0.02]	.862	0.07 [0.02, 0.12]	.026	0.01 [−0.04, 0.06]	>.999
Political Orientation × Believe	0.08 [0.04, 0.13]	.002	0.09 [0.04, 0.13]	.006	0.03 [−0.00, 0.07]	.314	−0.07 [−0.11, −0.02]	.049	−0.05 [−0.08, −0.01]	.145	−0.06 [−0.10, −0.02]	.080	0.12 [0.08, 0.17]	<.001	−0.06 [−0.11, −0.00]	.284
Political Orientation^2^ × Spread	−5.43 [−13.86, 2.99]	.620	−5.97 [−14.98, 3.04]	.583	−5.03 [−13.42, 3.36]	.323	3.22 [−5.64, 12.08]	>.999	−3.85 [−12.07, 4.38]	>.999	6.26 [−2.12, 14.64]	.862	−6.64 [−15.33, 2.04]	.269	7.26 [−1.78, 16.29]	.695
Political Orientation^2^ × Believe	−4.00 [−11.66, 3.66]	.620	−3.41 [−11.93, 5.11]	.583	−6.57 [−13.48, 0.34]	.314	2.17 [−6.02, 10.36]	>.999	0.13 [−6.43, 6.70]	>.999	3.08 [−4.71, 10.87]	>.999	−9.34 [−17.31, −1.37]	.088	6.84 [−2.34, 16.02]	.695
Spread × Believe	1.31 [1.00, 1.62]	<.001	2.71 [2.37, 3.06]	<.001	1.38 [1.08, 1.68]	<.001	−1.34 [−1.71, −0.96]	<.001	−0.32 [−0.65, 0.00]	.414	−1.60 [−1.96, −1.25]	<.001	2.05 [1.74, 2.36]	<.001	−1.11 [−1.49, −0.73]	<.001
GCB × Spread ×Believe	−0.06 [−0.13, 0.01]	.585	**−0.13** [−0.21, −0.05]	**.013**	−0.06 [−0.13, 0.01]	.314	0.01 [−0.07, 0.10]	>.999	−0.09 [−0.17, −0.02]	.156	0.08 [−0.00, 0.16]	.421	−0.07 [−0.15, −0.00]	.127	−0.07 [−0.16, 0.02]	.695
Political Orientation × Spread × Believe	−0.04 [−0.07, −0.00]	.301	−0.05 [−0.09, −0.01]	.084	−0.03 [−0.07, 0.00]	.308	0.01 [−0.04, 0.05]	>.999	0.04 [0.00, 0.08]	.377	0.00 [−0.04, 0.04]	>.999	**−0.06** [−0.10, −0.03]	**.003**	0.01 [−0.03, 0.05]	>.999
Political Orientation^2^ × Spread × Believe	5.41 [−0.90, 11.71]	.558	8.99 [1.87, 16.12]	.084	**8.69** [2.56, 14.82]	**.044**	0.02 [−7.63, 7.68]	>.999	4.74 [−1.99, 11.48]	.839	−5.76 [−13.06, 1.54]	.853	**10.68** [4.34, 17.03]	**.007**	−8.55 [−16.34, −0.76]	.284
Random effects	Variance (*SD*)		Variance (*SD*)		Variance (*SD*)		Variance (*SD*)		Variance (*SD*)		Variance (*SD*)		Variance (*SD*)		Variance (*SD*)	
Participants intercept	0.88 (0.94)		1.01 (1.01)		1.02 (1.01)		0.96 (0.98)		1.03 (1.02)		0.86 (0.93)		0.83 (0.91)		1.13 (1.06)	
Spread	1.65 (1.28)		1.81 (1.34)		1.66 (1.29)		1.62 (1.27)		1.47 (1.21)		1.43 (1.20)		1.79 (1.34)		1.66 (1.29)	
Believe	1.34 (1.16)		1.63 (1.28)		1.01 (1.01)		1.31 (1.15)		0.72 (0.85)		1.19 (1.09)		1.49 (1.22)		1.86 (1.36)	
Trial	0.00 (0.04)		0.00 (0.03)		0.00 (0.06)		0.00 (0.02)		0.00 (0.00)		0.00 (0.06)		0.00 (0.06)		0.00 (0.06)	
*R* ^2^																
Conditional	.85		.86		.86		.81		.79		.81		.88		.81	
Marginal	.26		.41		.31		.42		.32		.40		.44		.26	

*Note.* Holm-corrected *p*-values are presented. Significant three-way interactions are presented in bold. *SE*s, *t*-values, and degrees of freedom for all analyses are reported in Tables S15 to S17 in the Supplemental Materials. CI = confidence interval. GCB = Generic Conspiracist Beliefs; *SE* = standard error.

**Figure 5.**
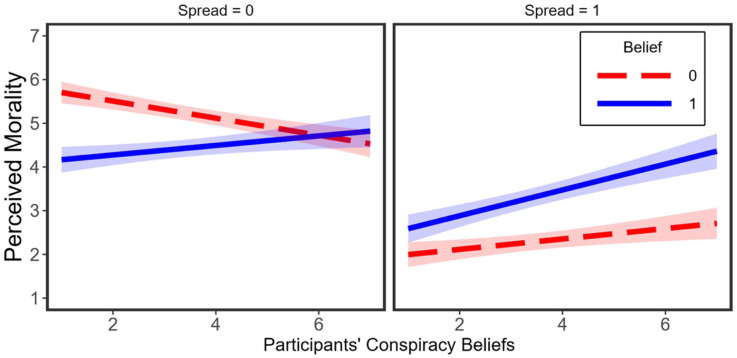
Three-way interaction between spread, believe, and participants’ conspiracy beliefs on perceived morality. *Note*. Ribbons represent 95% CIs. CI = confidence interval.

In relation to participants’ conspiracy beliefs, the three-way interaction involving perceived morality was significant, as illustrated in Figure 5. Specifically, the stronger participants themselves held conspiracy beliefs, the more moral they perceived the fictional character who believed in conspiracy theories to be when this character did not spread them (left panel, blue line), *B* = 0.11, 95% CI [0.02, 0.20], standard error (*SE*) = 0.05, *t* = 2.41, *p* = .016, but this effect was stronger when the character also spread them (right panel, blue line), *B* = 0.30, 95% CI [0.20, 0.39], *SE* = 0.05, *t* = 5.97, *p* < .001.

Furthermore, the more participants endorsed conspiracy beliefs themselves, the more moral they perceived the character who spread conspiracy theories without actually believing them to be (right panel, red line), *B* = 0.12, 95% CI [0.03, 0.20], *SE* = 0.04, *t* = 2.75, *p* = .006. By contrast, the more participants themselves held conspiracy beliefs, the less moral they perceived the characters who neither spread nor believed in conspiracy theories (left panel, red line), *B* = −0.20, 95% CI [−0.27, −0.12], *SE* = 0.04, *t* = −5.24, *p* < .001. No other significant three-way interactions with participants’ conspiracy beliefs were observed.

Concerning participants’ political orientation, a significant three-way interaction was observed in relation to perceived warmth of the fictional characters and the quadratic term of political orientation. Similarly, for willingness to have contact with the characters, significant interactions were noted, involving both the linear and quadratic terms of political orientation. No other significant three-way interactions with political orientation were found.

**Figure 6.**
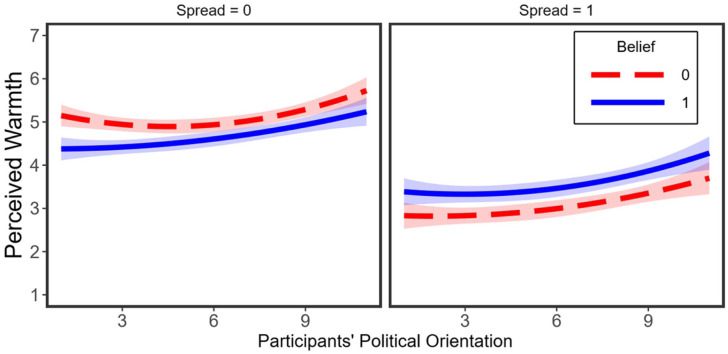
Three-way interaction between spread, believe, and participants’ political orientation on perceived warmth. *Note*. Ribbons represent 95% CIs. CI = confidence interval.

The results concerning perceived warmth are displayed in Figure 6. Generally, the slopes were relatively flat from the left to the middle of the political spectrum, with perceptions of warmth increasing especially when moving to the right-wing. One exception concerned the evaluation of the warmth of characters who do not spread and do not believe (left panel, red line), who seemed to be perceived somewhat warmer by participants on both ends of the political spectrum.

**Figure 7.**
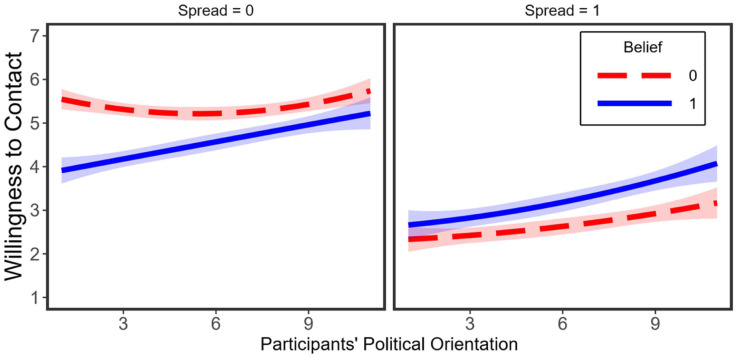
Three-way interaction between spread, believe, and participants’ political orientation on willingness to contact. *Note*. Ribbons represent 95% CIs. CI = confidence interval.

Next, as visualized in Figure 7 (left panel, blue line), there was a relatively linear and positive association between political orientation and willingness to have contact with individuals who believed in conspiracy theories but did not spread them, *B* = 0.13, 95% CI [0.09, 0.17], *SE* = 0.02, *t* = 5.96, *p* < .001. Thus, right-wing individuals showed a higher contact willingness than left-wing individuals here. A similar association was observed in terms of willingness to have contact with the characters who believed in conspiracy theories and spread them (right panel, blue line), *B* = 0.14, 95% CI [0.09, 0.19], *SE* = 0.03, *t* = 5.50, *p* < .001, but this effect accelerated toward the right-wing side of the political orientation scale as indicate by a slightly curved slope. In terms of the characters who neither spread nor believed (left panel, red line), the linear association with political orientation was not significant, *B* = 0.01, 95% CI [−0.02, 0.04], *SE* = 0.02, *t* = 0.60, *p* = .549. However, a slight curvilinear relationship was observed with political orientation, with both left- and right-wing participants tending to show a slightly higher contact willingness than politically moderate participants. For the characters who spread but did not believe in conspiracy theories (right panel, red line), the association with contact willingness was relatively linear. Specifically, participants with a right-wing political orientation demonstrated a higher contact willingness, *B* = 0.08, 95% CI [0.04, 0.12], *SE* = 0.02, *t* = 3.82, *p* < .001.

#### Pre-Registered Exploratory Analyses

We found no significant three-way interactions between the factors “spread,” “believe,” and the gender of the fictional characters across all models (see Tables S18–S20 in the Supplemental Materials).

### Preliminary Discussion

Based on prior work, we expected to find evidence that individuals are perceived as higher or lower on certain traits depending on whether they believed (vs. did not believe) and/or spread (vs. did not spread) conspiracy theories. We indeed found that people who engage with conspiracy theories are generally perceived less favorably than people who do not engage with conspiracy theories. Importantly, we demonstrate that spreading and believing conspiracy theories are influential characterizing features, which can have distinctive social consequences. While believers received relatively less favorable ratings than non-believers, we found that spreaders—and particularly non-believing spreaders—received the most negative assessments.

This first study employed a relatively common top-down approach to examine how individuals and groups are perceived, requiring participants to explicitly rate targets on predefined traits (e.g., Cuddy et al., 2008, 2009; Imhoff et al., 2013). Although this method provides valuable insights, it remains uncertain whether similar effects would be observed using data-driven methods assessing variables at a less explicit level.

To address this question, the second study adopted a reverse-correlation approach, allowing us to test these effects in a more subtle and less directive manner, capturing implicit mental representations of individuals who differ in whether they believe (vs. do not believe) and/or spread (vs. do not spread) conspiracy theories.

## Study 2

This pre-registered second study aimed to build on the findings of Study 1 by investigating whether the verbal descriptions of individuals who engage with conspiracy theories align with how they are mentally represented. Using the reverse-correlation technique (Dotsch & Todorov, 2012), we examined the mental representations participants had of individuals who differed in terms of whether they believed (vs. did not believe) and/or spread (vs. did not spread) conspiracy theories. All hypotheses and the analysis plan were pre-registered on the OSF at https://osf.io/52kcm?view_only=067540c4d3da4064be64e82fcced5e77.

The reverse-correlation technique (Brinkman et al., 2017; Dotsch & Todorov, 2012) is a data-driven method in which participants are repeatedly presented with two (or more) adjacent stimuli—visually similar faces with slight differences—and asked to select the one that better fits a specific target category (e.g., gender, ethnicity). To create variation between the stimuli, random noise patterns are superimposed on the same base image (e.g., an average White male or female face). Because these noise patterns are randomly generated, each stimulus face appears slightly different. In the two-image version used in this study, the differences between images are maximized as the inverse noise pattern is applied to one of the images. After participants complete a series of trials, researchers calculate the average image selected by each participant or condition, resulting in an approximation of their mental representation of the target category as perceived by the participants. The resulting image is typically referred to as a “classification image.”

In a subsequent step, independent raters, blind to the hypotheses, evaluate these classification images based on dimensions of interest to the researchers. This technique has been used to identify features that distinguish faces associated with, for example, various ethnicities, nationalities, and personality traits (for an overview, see Brinkman et al., 2017). Importantly, it has also demonstrated how moderating participant characteristics, such as prejudice (Dotsch et al., 2008) or sexism (Gundersen & Kunst, 2019), can influence these classification images.

Here, we used the same experimental conditions as in Study 1, however in a between-subjects design in the image generation part, to investigate how the classification images of individuals who believe (vs. do not believe) and/or spread (vs. do not spread) conspiracy theories are mentally represented across several dimensions: competence, morality, warmth, Machiavellianism, narcissism, and psychopathy. We also addressed whether these classification images suggested intentions to conspire, the extent to which participants were willing to have contact with such individuals, and the gender most associated with the imagined faces (i.e., most resembling a man or a woman).

Based on the findings from Study 1, we formulated slightly revised hypotheses regarding how individuals would be ranked according to whether they believe (vs. do not believe) and/or spread (vs. do not spread) conspiracy theories. More specifically, these hypotheses were updated to align with the results obtained in Study 1, anticipating distinct scores across the measured dimensions—listed here in order from highest to the lowest:

**H1:** Competence: neither believes nor spreads > believes but does not spread > believes and spreads = spreads but does not believe.
**H2:** Morality: neither believes nor spreads > believes but does not spread > believes and spreads > spreads but does not believe.
**H3:** Warmth: neither believes nor spreads > believes but does not spread > believes and spreads > spreads but does not believe.
**H4:** Machiavellianism^4^: spreads but does not believe > believes and spreads > believes but does not spread > neither believes nor spreads.
**H5:** Narcissism: spreads but does not believe > believes and spreads > believes but does not spread = neither believes nor spreads.
**H6:** Psychopathy: spreads but does not believe > believes and spreads > believes but does not spread > neither believes nor spreads.
**H7:** Conspiracy intentions: spreads but does not believe > believes and spreads > believes but does not spread > neither believes nor spreads.
**H8:** Contact willingness: neither believes nor spreads > believes but does not spread > believes and spreads > spreads but does not believe.

Furthermore, as pre-registered and informed by the findings of Study 1, we hypothesized that classification images of individuals who believe in and/or spread conspiracy theories would be perceived as more moral when these images were generated by participants scoring high (vs. low) on the GCB Scale (Kay & Slovic, 2023). Conversely, we anticipated that classification images of individuals who neither believe in nor spread conspiracy theories would be perceived as less moral when images of them were generated by participants high (vs. low) in conspiracy beliefs.

Additionally, we predicted that classification images of individuals who believe in and/or spread conspiracy theories would be perceived as warmer, and that participants would express greater willingness to have contact with them when these images were generated by individuals on the political right (vs. political left). However, we did not expect political orientation to influence the ratings of classification images for individuals who neither believe in nor spread conspiracy theories.

### Methods

We report how we determined our sample size, all data exclusions, all manipulations, and all measures in the study.

#### Participants

For Step 1 of this study, the two-image forced-choice task, we conducted an a priori power simulation using a shiny app based on the InteractionPowerR package (Baranger et al., 2023) in R. The simulation results based on 1,000 iterations revealed that, with 200 participants contrasting two out of four between-group total conditions, we could achieve over 90% power to detect a two-way interaction effect (β = .21) between a binary and a continuous variable, assuming an α level of .05. This chosen effect size corresponds to the smallest two-way interaction effect that was significant at *p* < .01 in Study 1. Additionally, considering the power achieved and our planned use of multi-level models in Step 2—incorporating 12 raters per trait, effectively increasing the number of cases—we anticipate sufficient power to detect a three-way interaction effect. Based on these findings, we set a recruitment target of at least 400 participants for Step 1 of the study (i.e., 100 participants per condition, totaling 4,800 ratings per trait).

As pre-registered, we excluded nine participants who failed two attention checks and ten participants who completed the survey in less than 10 min. The 10-min cut-off was adopted from a prior reverse-correlation study (Kunst, Juettemeier, et al., 2024). The final sample consisted of 412 U.S. adults aged 20 to 78 years (*M*_age_ = 41.43, *SD*_age_ = 12.37). This sample size affords the same level of power as estimated in the a priori power simulation, as the two conditions with the fewest participants total *n* = 200 (see condition descriptions below). Participants were recruited through Prolific using quota sampling to ensure gender balance and alignment with the U.S. census distribution for ethnicity and political affiliation at the time of data collection. Data collection took place at the end of April 2024, and all participants were compensated at a rate equivalent of £9.00/hr. Detailed demographic information from Step 1 is provided in the Supplemental Materials, Table S21.

For Step 2 of this study, the image-rating task, we conducted an a priori multi-level power simulation using the lme4 (Bates et al., 2015) and simr (Green & MacLeod, 2016) packages in R. The simulation indicated that with 108 participants (12 raters per trait across nine traits), we would achieve 97% power to detect a two-way interaction effect (β = .20), assuming an α level of .05. Please note that, due to random sampling variability produced by the power simulation, the observed effect size in this step differed slightly from the expected effect size in Step 1. The model included 400 Level 2 units (target sample size in Step 1), with 12 Level 1 units nested within each Level 2 unit (12 raters per trait), resulting in a total of 4,800 ratings per trait. Given this multi-level structure, we were confident that we had sufficient power to detect three-way interactions as well. To further increase power and account for the highly repetitive nature of the task, which may increase attrition, we intentionally oversampled by recruiting additional participants beyond the minimum required *n* for adequate power.

As pre-registered, we excluded seven participants who failed two attention checks and four participants who completed the task in under 10 min. The final sample consisted of 183 U.S. raters aged 18 to 78 years (*M*_age_ = 45.82, *SD*_age_ = 15.78; 51.6% women) quota-representative of the U.S. population in terms of gender, age, ethnicity, race, and education. This expanded sample effectively increased the number of Level 1 units to approximately 20 raters per trait, who evaluated 412 images (Level 2 units) across nine dimensions, resulting in 8,240 ratings per trait. Ratings were nested within the classification images generated in Step 1. This final sample size thus provided 98% power to detect a two-way interaction effect size of β = .20 at an α level of .05. Participants were recruited through CloudResearch Connect. All participants were compensated approximately $12.00/hr for their time. Detailed demographic information for Step 2 is provided in the Supplemental Materials, Table S22.

#### Procedure and Materials

This study was approved by the Institutional Review Board at the department affiliated with the first and last authors (No. 26123962). Materials, R code, and data for this study are available at https://osf.io/k34qm/. The survey for both steps was administered using Qualtrics.

##### Step 1: Image Generation

**Figure 8.**
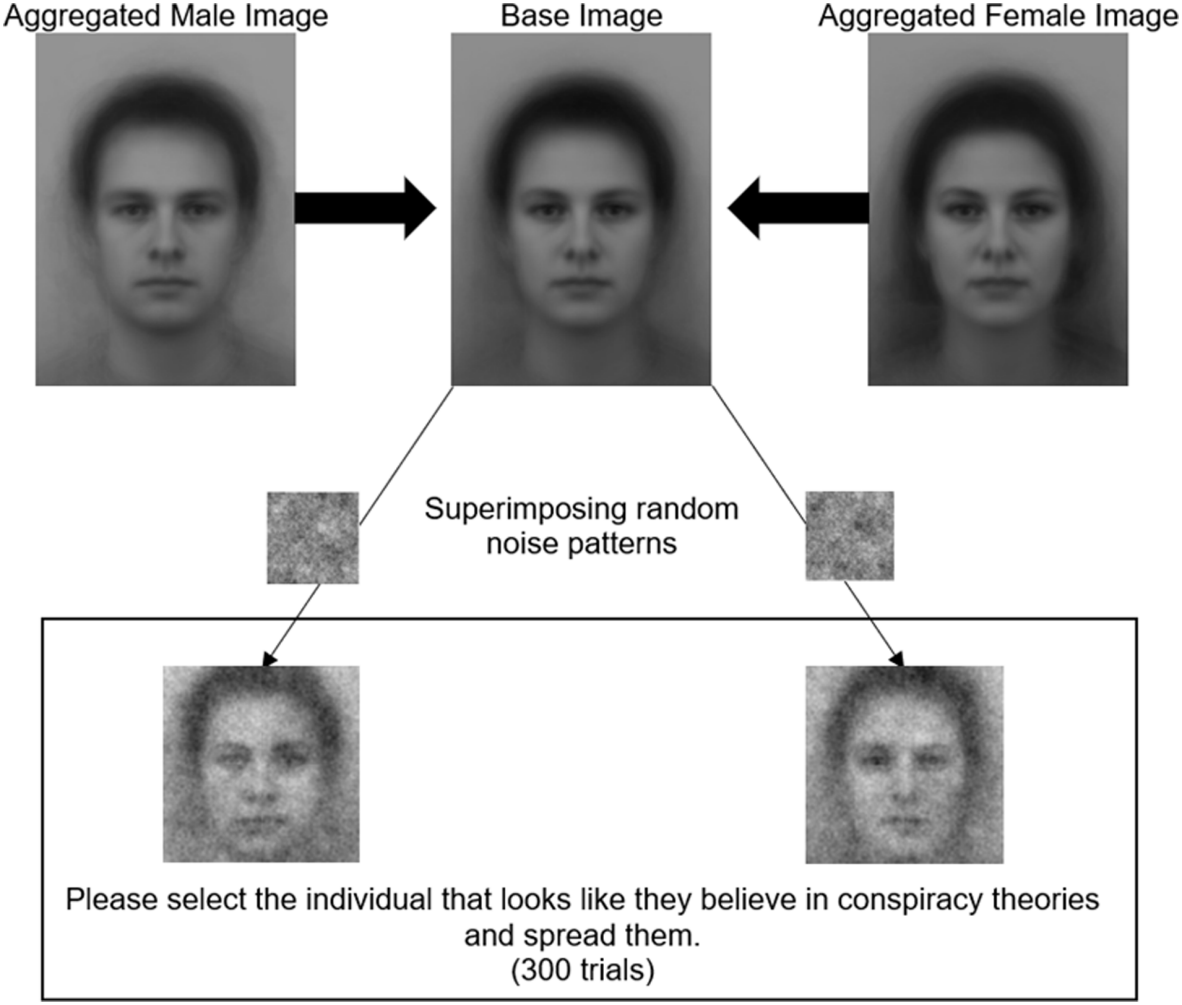
Base image creation and an example of the two-image forced-choice task in Step 1. *Note*. Pre-testing revealed that the base image perceived as the most “gender neutral” consisted of 40% opacity from the aggregated male image and 60% opacity from the aggregated female image.

After providing informed consent, participants completed a two-image forced-choice task using the reverse-correlation technique (Dotsch & Todorov, 2012; Dotsch et al., 2008) to assess their mental representations of individuals who differ in terms of whether they spread (vs. do not spread) and/or believe (vs. do not believe) in conspiracy theories. Specifically, as illustrated in Figure 8, a gender neutral facial base image was created by averaging aggregated male and female facial images from the Karolinska Face Database (Lundqvist et al., 1998). A pre-test determined that a base image with 40% opacity of aggregated male images and 60% opacity of aggregated female images was perceived as the most gender neutral (details of the pre-test are available in the Supplemental materials).

Next, we superimposed random noise patterns to the base image using default parameters, generating 600 visually altered versions of it (Dotsch, 2023). Half of these images featured original random noise patterns, while the other matched half used the mathematical inverted noise patterns. Participants were randomly assigned to one of four experimental conditions and completed 300 trials. In each trial, they were presented with two adjacent images—one with an original noise pattern and the other with its inverse—and asked to select the image that best matched a specific description based on the between-group condition they had been randomly assigned to. The descriptions were: “Please select the individual who looks like they neither believe in conspiracy theories nor spread them” (*n* = 89), “. . . believe in conspiracy theories but do not spread them” (*n* = 108), “. . . do not believe in conspiracy theories but spread them” (*n* = 114), or “. . . believe in conspiracy theories and spread them” (*n* = 101).

To create individual classification images, the noise patterns from the images selected by each participant were extracted and superimposed on the base image. This process resulted in a visual approximation of how participants mentally represented a person who engaged with conspiracy theories across the 300 trials, depending on their assigned condition. Additionally, we generated average classification images from each experimental condition. After completing the two-image forced-choice task, participants proceeded to complete the additional measures described below.

After completing the image selection task, participants’ conspiracy beliefs were measured using the 5-item short form of the GCB Scale (Kay & Slovic, 2023). Participants rated items (e.g., “The government permits or perpetrates acts of terrorism on its own soil, disguising its involvement”) on a scale from 1 (*strongly disagree*) to 7 (*strongly agree; M* = 3.73, *SD* = 1.40, α = .83). Classification images were generated based on median splits (median = 3.80) of participants’ scores on this scale within each experimental condition. One attention check was embedded among these items, asking participants to select a specific response option (i.e., “It is important that you pay attention to this study. Please tick ‘Disagree’”).

Before being debriefed, participants completed standard demographic questions (see Table S21 in the Supplemental Materials) and indicated their political orientation on a scale from 1 (*very liberal/left-wing*) to 11 (*very conservative/right-wing; M* = 5.37, *SD* = 2.92). Classification images were also generated based on median splits (median = 6.00) of participants’ political orientation within each condition. A second attention check was included among the demographic items, instructing participants to select a specific color (i.e., “The color test you are about to take part in is very simple. When asked for your favorite color, you must select ‘black’. This is an attention check.”).

##### Step 2: Image Rating Task

After obtaining informed consent, participants rated the classification images generated in Step 1. A total of 432 classification images were evaluated: 412 individual classification images, one image aggregated across participants from each of the four conditions, eight images aggregated across participants based on median splits of the GCB-5 within each condition, and eight images aggregated across participants based on median splits of political orientation within each condition. Participants rated the images on several traits, including competence, morality, warmth, Machiavellianism, narcissism, and psychopathy, using a 7-point scale ranging from 1 (*not at all*) to 7 (*extremely*). Before providing their ratings, participants were presented with descriptions of each trait (see Section 2 in the Supplemental Materials).

Participants also rated their willingness to have contact with the individuals depicted in the images. Specifically, they responded to the question: “How would you feel about having this person as a neighbor?” on a scale from 1 (*very negative*) to 7 (*very positive*).

To measure perceived conspiracy intentions, participants indicated their agreement with the statement: “This person is open to conspiring with others on hidden agendas that prioritize personal or group gain over the common good,” using a scale from 1 (*strongly disagree*) to 7 (*strongly agree*).

The perceived gender of the individuals in the images was assessed by asking participants to rate to what extent the individuals appeared mostly like a man or a woman, rated from 1 (*definitely a man*) to 11 (*definitely a woman*). Each participant was randomly assigned to evaluate the classification images on only one of these dimensions, resulting in 17 to 23 raters per dimension.

To ensure data quality, three attention checks were interspersed randomly among the images, instructing participants to select a specific response option (see Figure S1 in the Supplemental Materials for an example). After completing the image-rating task, participants answered standard demographic questions (i.e., gender, age, ethnicity, political orientation, education, and income) before being debriefed.

#### Analyses

Based on recommendations for rating classification images (see Cone et al., 2021), we had participants rate individual classification images in addition to aggregated condition-level images. Our analysis employed multi-level modeling, with ratings from Step 2 nested within the classification images generated by participants in Step 1. We tested the main effects and interactions, including a three-way interaction: 2 (spread vs. do not spread) × 2 (believe vs. do not believe) × political orientation/conspiracy beliefs.

Please note that we, deviating from the pre-registration, bootstrapped (*N*_sim_ = 1,000) all our multi-level models to obtain robust and reliable estimates of uncertainty, given the structure of our data and some violations of assumptions observed in Study 1 (see Supplemental Materials). Multi-level models account for nested dependencies (e.g., repeated measures within participants, ratings nested within images), which can lead to non-normality and heteroskedasticity in residuals (Snijders & Bosker, 2012). Traditional parametric methods for estimating *SE*s and CIs rely on assumptions that may not be held in hierarchical data structures. Bootstrapping provides a non-parametric approach that mitigates these concerns by repeatedly resampling the data and estimating the model across resampled datasets (van der Leeden et al., 2008). Note that the pattern of results remained the same without this approach (see Table S23 in the Supplemental Materials for bias estimates).

Ratings of the aggregated images for each condition and the median splits of political orientation and conspiracy beliefs were analyzed using repeated-measures analyses of variance (ANOVAs). Each dimension (e.g., competence, morality, etc.) was analyzed separately, with all *p-*values adjusted using the Holm-method. The same software and R packages as in Study 1 were used.

### Results

**Figure 9.**
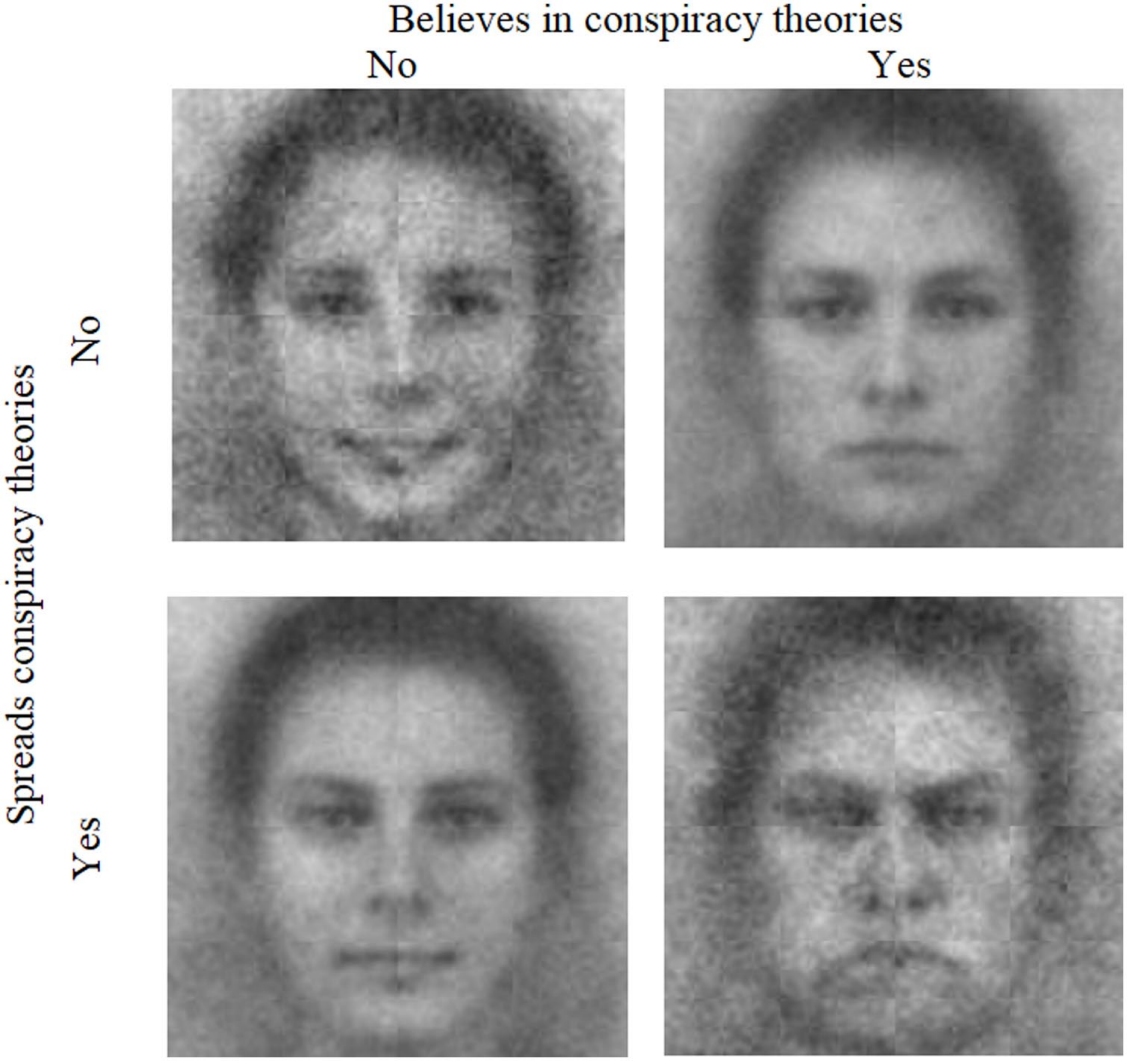
Aggregated classification images for the experimental conditions.

To evaluate the informational value of the classification images, we calculated the infoVal statistic, which estimates the probability that an observed classification image deviates from those generated by random noise (i.e., distinguishing signal from noise). The infoVal statistic can be interpreted as a *z*-score. The obtained infoVal statistics were relatively low for the individual classification images (*M* = 1.33, *SD* = 1.19), and across conditions (neither believe nor spread: *M* = 1.48, *SD* = 1.13; only believe: *M* = 1.25, *SD* = 1.26; only spread: *M* = 1.33, *SD* = 1.26; both believe and spread: *M* = 1.29, *SD* = 1.12). By contrast, the aggregated classification images for each experimental condition showed much higher infoVal statistics, with *z*-scores ranging from 16.59 to 19.43. The aggregated classification images for the four experimental conditions are shown in Figure 9, not only provided primarily for visualization purposes but also analyzed later on.

To test our main hypotheses, we conducted separate multi-level models for each dimension, setting random intercepts for participants and trials, as well as random effects for the “spread” and “believe” factors. In these models, the fixed effects analysis revealed no significant interactions between the factors “spread” and “believe” on any outcome measure (all *ps* > .05; see Tables S24–S26 in the Supplemental Materials for details). To facilitate the interpretation of the main effects of “spread” and “believe,” we re-ran the models with both variables effect-coded. Across all models, we observed significant main effects of both the “spread” and “believe” factors on the various rating dimensions. The results of our multi-level linear models are summarized in Table 5.

**Table 5.** Results of Multi-Level Linear Models: Examining the Fixed Effects of Spread and Believe on Dependent Variables, With Control for Random Intercepts and Effects.

Effect	Competence		Morality		Warmth		Machiavellianism		Narcissism		Psychopathy		Conspiracy intentions		Contact willingness	
	*B* [95% CI]	*p*	*B* [95% CI]	*p*	*B* [95% CI]	*p*	*B* [95% CI]	*p*	*B* [95% CI]	*p*	*B* [95% CI]	*p*	*B* [95% CI]	*p*	*B* [95% CI]	*p*
Fixed effects																
(Intercept)	3.69 [3.23, 4.12]	<.001	3.81 [3.56, 4.08]	<.001	3.49 [3.04, 3.97]	<.001	3.83 [3.61, 4.11]	<.001	3.70 [3.45, 3.99]	<.001	3.52 [3.14, 4.01]	<.001	3.74 [3.49, 3.98]	<.001	3.98 [3.79, 4.23]	<.001
Spread	−0.26 [−0.37, −0.13]	<.001	−0.34 [−0.46, −0.18]	<.001	−0.35 [−0.51, −0.17]	<.001	0.22 [0.10, 0.31]	.001	0.22 [0.05, 0.34]	.002	0.47 [0.30, 0.62]	<.001	0.36 [0.20, 0.51]	<.001	−0.37 [−0.51, −0.23]	<.001
Believe	−0.44 [−0.57, −0.29]	<.001	−0.47 [−0.64, −0.33]	<.001	−0.50 [−0.67, −0.32]	<.001	0.27 [0.12, 0.40]	.003	0.33 [0.11, 0.50]	.002	0.63 [0.47, 0.82]	<.001	0.50 [0.35, 0.68]	<.001	−0.54 [−0.70, −0.32]	<.001
Spread × Believe	−0.06 [−0.29, 0.19]	.621	−0.04 [−0.24, 0.16]	.763	−0.08 [−0.30, 0.18]	.573	0.04 [−0.08, 0.19]	.592	0.09 [−0.08, 0.28]	.338	0.08 [−0.34, 0.48]	.639	0.09 [−0.13, 0.35]	.524	−0.09 [−0.36, 0.17]	.457
Random effects	Variance (*SD*)		Variance (*SD*)		Variance (*SD*)		Variance (*SD*)		Variance (*SD*)		Variance (*SD*)		Variance (*SD*)		Variance (*SD*)	
Participants intercept	0.79 (0.88)		0.40 (0.64)		0.79 (0.89)		0.48 (0.70)		0.47 (0.69)		1.08 (1.04)		0.32 (0.56)		0.40 (0.63)	
Spread	0.03 (0.17)		0.06 (0.24)		0.05 (0.21)		0.05 (0.21)		0.05 (0.22)		0.03 (0.17)		0.02 (0.15)		0.03 (0.17)	
Believe	0.08 (0.29)		0.08 (0.28)		0.08 (0.28)		0.11 (0.33)		0.17 (0.41)		0.06 (0.24)		0.05 (0.22)		0.08 (0.28)	
Trial	0.27 (0.52)		0.32 (0.57)		0.44 (0.67)		0.10 (0.32)		0.14 (0.37)		0.59 (0.77)		0.41 (0.64)		0.36 (0.60)	
*R* ^2^																
Conditional	.53		.49		.65		.36		.30		.58		.41		.50	
Marginal	.03		.05		.04		.02		.02		.04		.04		.06	

*Note.* Variables are effect coded. *SE*s, *t*-values, and degrees of freedom for all analyses are reported in Tables S27 to S29 in the Supplemental Materials. CI = confidence interval; *SE* = standard error.

The results indicated that the classification images of individuals who believe in and/or spread conspiracy theories were perceived as significantly less competent, moral, and warm, while being seen as more Machiavellian, narcissistic, and psychopathic compared to those of individuals who did not engage in such beliefs or behaviors. Additionally, conspiracy believers and spreaders were perceived as having significantly higher intentions to conspire, and participants reported being less positive about contact with them. Taken together, main effects but not interactions provided partial support for Hypotheses H1 to H8. Mean scores for the rating dimensions across conditions are visualized in Figure S2 in the Supplemental Materials.

To test whether spreading and believing in conspiracy theories had significantly different effects on the rating dimensions, we estimated bootstrapped 95% CIs (*N*_sim_ = 1,000) for the difference between the two effects. Results indicated that believing had a significantly stronger effect than spreading on perceived competence, ∆*B* = −0.18, 95% CI [−0.23, −0.11], morality, ∆*B* = −0.13, 95% CI [−0.18, −0.07], warmth, ∆*B* = −0.15, 95% CI [−0.20, −0.10], narcissism, ∆*B* = 0.12, 95% CI [0.04, 0.19], psychopathy, ∆*B* = 0.17, 95% CI [0.10, 0.24], conspiracy intentions, ∆*B* = 0.14, 95% CI [0.08, 0.21], and willingness for contact, ∆*B* = −0.17, 95% CI [−0.21, −0.12]. There was no significant difference between the effects of spreading and believing on perceived Machiavellianism, ∆*B* = 0.05, 95% CI [−0.01, 0.11].

For exploratory purposes, we examined how the classification images were perceived on a spectrum from man to woman. Significant effects were observed for both “spread,” *B* = −0.56, 95% CI [−0.83, −0.35], *SE* = 0.13, *t*(60.70) = −4.24, *p* < .001, and “believe,” *B* = −0.60, 95% CI [−0.92, −0.34], *SE* = 0.14, *t*(48.86) = −4.33, *p* < .001. Estimated marginal means suggested that the classification image of the individual who neither spread nor believed in conspiracy theories appeared more woman-like (*M* = 6.14, *SE* = 0.39). In contrast, the images of individuals who spread but did not believe (*M* = 5.58, *SE* = 0.39), and those who believed but did not spread (*M* = 5.53, *SE* = 0.39) appeared more man-like. Finally, the image of the individual who both spread and believed in conspiracy theories was perceived as more man-like (*M* = 4.97, *SE* = 0.41).

#### Interactions With Participants’ Conspiracy Beliefs and Political Orientation

As pre-registered, we next tested whether classification images of individuals who spread and/or believe in conspiracy theories were perceived as more moral when generated from participants with high scores on the GCB-5. Additionally, we examined whether these classification images were perceived as warmer and whether participants exhibited greater willingness to have contact with them when the images were generated by participants on the political right compared to the political left. To address these hypotheses, we employed an additional set of multi-level models. In these models, political orientation was included as both a linear and a quadratic effect at Level 2. Consistent with our previous models, we set random intercepts for participants and trials as well as random effects for “spread” and “believe.”

No significant two-way interactions were observed between the factors (i.e., spread and believe) and the conspiracy beliefs of the participants who generated the images for any variable (see Tables S30–S32 of the Supplemental Materials).

Next, we tested for three-way interactions: 2 (spread vs. do not spread) × 2 (believe vs. do not believe) × political orientation/conspiracy beliefs. None of these interaction effects were significant (see Tables S33–S35 in the Supplementary Materials).

#### Ratings of Aggregated Classification Images

As pre-registered, we conducted separate repeated-measures ANOVAs for each rating dimension using the aggregated classification images from each condition, as well as those generated based on median splits of participants’ conspiracy beliefs (low vs. high) and political orientation (left vs. right) within each condition. However, since this approach is generally not recommended (Cone et al., 2021), these results are provided in the Supplemental Materials for interested readers (see Section S3).

### Preliminary Discussion

In this second pre-registered online experiment, we aimed to test whether the verbal descriptions of individuals who engage with conspiracy theories, as examined in Study 1, also extended to how they are mentally represented from a bottom-up perspective. While we found that representations of both believers and spreaders were generally rated less favorably than their non-believing and non-spreading counterparts, the effects were relatively modest. Specifically, the representations of both believers (compared to non-believers) and spreaders (compared to non-spreaders) were perceived as significantly less competent, moral, and warm, and more Machiavellian, narcissistic, and psychopathic. These representations were also seen as having higher intentions to conspire, and participants expressed less positivity about having them as neighbors. Interestingly, although spreading was the stronger driver of these effects in Study 1, believing exerted a significantly greater influence on these perceptions in the current study.

We found no evidence that participants’ conspiracy beliefs or political orientation significantly influenced their visual representations. While some previous reverse-correlation studies have shown that explicit measures influence visual representations, others have not. For example, Dotsch et al. (2008) found that participants with higher prejudice scores generated more stereotypical-looking mental representations of stigmatized foreigners. Similarly, Gundersen and Kunst (2019) reported that individuals scoring high on hostile sexism produced more masculine-looking representations of feminist women. In contrast, Petsko et al. (2021) found no significant differences in how U.S. participants visually represented Arabs, regardless of their level of explicit dehumanization. The lack of influence from explicit measures in this study suggests that individuals who engage with conspiracy theories may be overall mentally represented more unfavorably than those who do not, possibly independent of the perceiver’s own explicitly reported level of conspiracy belief or political orientation.

## General Discussion

Across two pre-registered online experiments, we systematically examined both the independent and interactive effects of *spreading* and *believing* in conspiracy theories on social perceptions—specifically, stereotype content, dark personality traits, perceived conspiracy intentions, and participants’ willingness to have contact with these individuals. Previous research has typically focused on either the effects of believing (e.g., Klein et al., 2015) or disseminating (e.g., Cao et al., 2025; Green et al., 2023) in isolation, rather than jointly exploring how these two factors shape perceptions when considered side by side. Our online experiments address this gap by demonstrating that both believing in and spreading conspiracy theories carry unique social consequences. Because perceptions of individuals and groups can strongly influence emotional responses, attitudes, and behavioral intentions (Cuddy et al., 2007, 2008), understanding how people evaluate those who engage with conspiracy theories holds both theoretical and practical significance for intergroup relations, political psychology, and misinformation/conspiracy theory research.

Study 1 employed a 2 × 2 within-subjects design, in which participants explicitly evaluated fictional individuals who believed (vs. did not believe) and/or spread (vs. did not spread) conspiracy theories across multiple dimensions (i.e., competence, morality, warmth, Machiavellianism, psychopathy, conspiracy intentions, and willingness to have contact with the individual). In Study 2, we aimed to extend these findings using a more subtle approach: we tested whether participants’ mental representations of individuals varied in accordance with the individuals’ belief in, and/or dissemination of, conspiracy theories. Specifically, we used a 2 × 2 between-subjects design in a two-step reverse-correlation procedure. First, we modeled participants’ mental representations of such individuals. Then, a second independent sample rated these representations on the same dimensions employed in Study 1.

### Independent and Interactive Effects of Spreading and Believing

Our findings from Study 1 indicate that individuals who believe in but do not spread conspiracy theories are viewed less favorably compared to non-believers. However, while they are generally perceived more negatively, they are, in absolute terms, still rated quite positively in terms of competence, morality, warmth, and willingness for contact, assessed in the upper half of the measurement scales for these positive traits. Conversely, they are positioned in the lower half of the measurement scales for negative traits such as perceived narcissism, Machiavellianism, psychopathy, and intentions to conspire. These results suggest that individuals who believe in conspiracy theories but do not disseminate them may be afforded a degree of leniency for their beliefs, facing less severe social penalties than those who spread conspiracy beliefs they do not hold. This interpretation is further supported by participants’ expressed willingness to interact with believers, which scored above the scale’s midpoint.

Our results from Study 1 indicate that the primary factor leading to negative perceptions of individuals involved with conspiracy theories is the act of spreading them. Participants evaluated those who disseminated conspiracy theories without believing in them the most negatively among all fictional characters and showed the lowest willingness for contact with them. This act of dissemination, particularly without personal belief, is akin to spreading disinformation, defined as the intentional sharing of false information to deceive, often for political or financial reasons (Fallis, 2015; Wardle & Derakhshan, 2017). The Global Risks Report by the World Economic Forum (2024) highlights the dissemination of disinformation and misinformation as humanity’s foremost global risk, and scholars have linked it to the proliferation of harmful misconceptions, such as vaccine skepticism and holocaust denial (Lewandowsky et al., 2024). Spreading false information without personal belief might be perceived as especially immoral and a violation of social norms, which could explain the particularly harsh judgments toward these disseminators.

While our findings from Study 2 were directionally consistent with those from Study 1, the pattern of results changed. Using the reverse-correlation technique (Dotsch & Todorov, 2012), we modeled visual representations of individuals engaging with conspiracy theories to investigate whether the verbal descriptions obtained in Study 1 extended to people’s mental representations. Our findings revealed that while believers and spreaders were perceived significantly less favorably across all rating dimensions compared to their non-believing and non-spreading counterparts, the effects were relatively modest. Moreover, belief in conspiracy theories emerged as the primary driver of these effects, rather than the spreading of conspiracy theories (as observed in Study 1). Because believing in conspiracy theories is generally more common than actively disseminating them (Douglas et al., 2019; Uscinski, Enders, Klofstad, et al., 2022), participants in Study 2 may have found the believer stereotype more readily accessible, making belief the more impactful factor. Our results suggest that although people hold strong verbal stereotypes about individuals who engage with conspiracy theories, their mental representations of such individuals are less clearly defined—yet appear notably stronger when those individuals are perceived as believing rather than merely spreading conspiratorial ideas.

This ambiguity may stem from the fact that individuals who engage with conspiracy theories are not a visually distinctive social group, and people likely hold varied expectations about what a “typical” person engaging with conspiracy theories looks like. For instance, in the image-selection task of Study 2, participants may have relied on diverse mental shortcuts when choosing facial images. Some might have selected the image that resembled a person they know who believes in conspiracy theories, others might have chosen one resembling a conspiracy-promoting politician, while some may have focused on selecting an image that contained particular facial features. As a result, the visual stereotype of individuals who engage with conspiracy theories may be highly heterogeneous within the general population. This notion is further supported by the relatively low infoVal statistics observed at the individual level, which indicate that there was more random noise and less signal than desired in the classification images across all conditions. However, the group-level classification images yielded much higher infoVal statistics, suggesting a more distinct visual stereotype when data are aggregated at the group level.

### The Role of Participants’ Own Conspiracy Beliefs

In Study 1, we found that the stronger a participant’s belief in conspiracy theories, the less negatively they perceived others who spread these beliefs on all dimensions. In cases involving fictional individuals who believed in conspiracy theories, we even observed a reversal of the typical effect. Specifically, participants with stronger personal conspiracy beliefs viewed these believers more favorably, perceiving them as warmer, more moral, and less narcissistic and Machiavellian, with correspondingly lower intentions for conspiratorial actions. These participants also showed a greater willingness to interact with fellow conspiracy believers. Intriguingly, this trend suggests that conspiracy believers prefer associating with like-minded individuals, yet they may penalize those who publicly spread these theories. A potential explanation for this could be a collective awareness among conspiracy believers of the stigma attached to such theories (e.g., Harambam & Aupers, 2017; Lantian et al., 2018), leading to a cautious approach toward dissemination to avoid further stigmatization, favoring discussion within more private spheres (e.g., social media groups). However, different effects might have emerged if we had tested the extent to which participants themselves shared conspiracy beliefs online.

In our studies, we not only explored how spreading and believing in conspiracy theories are separately influenced by participants’ own conspiracy beliefs but also examined the combined effect of these factors on social perceptions and contact willingness. Notably, in Study 1, participants with stronger personal conspiracy beliefs perceived individuals engaging with conspiracy theories (whether believing, spreading, or both) as more moral when compared to participants with less conspiracy beliefs. Conversely, individuals who neither believed nor spread conspiracy theories were seen as less moral by participants with stronger conspiracy beliefs. Framing these findings within the context of intergroup relations, our results contribute to the body of literature demonstrating ingroup favoritism in the evaluation of ingroups and outgroups (Brewer, 2017; Everett et al., 2015). Individuals engaging with conspiracy theories were likely viewed as more moral because they were assumed to share common values, beliefs, and attitudes with the participants, leading to more positive evaluations on this dimension as possibly perceptions of shared group membership. Conversely, individuals abstaining from conspiracy theories might be viewed as part of an outgroup (e.g., ignorant “sheeple”; see Franks et al., 2017), resulting in perceptions of them as less moral. This aligns with prior work showing that individuals low in conspiracy beliefs tend to attribute endorsement of such beliefs to intra-individual causes (e.g., reasoning biases or paranoia), whereas individuals high in conspiracy beliefs are less likely to do so (Leveaux et al., 2022). Our research extends this line of work by examining not only belief but also spread, and by focusing on more fine-grained social perceptions, including specific trait judgments and contact willingness. Another potential explanation, however, is that conspiracy beliefs themselves may be partially motivated by moral concerns. Conspiratorial narratives often frame the purported conspiracy as a moral violation (e.g., see Galgali et al., 2024), and those who reject such beliefs may therefore be perceived as dismissing the seriousness of this violation. In this way, perceiving non-believers as immoral may stem not only from intergroup dynamics but also from moral judgments that operate independently of group membership.

Despite the moderating effects of personal conspiracy beliefs on participants’ ratings in Study 1, we found no such moderation in Study 2. Specifically, no significant differences emerged between the mental representations generated from participants with strong conspiracy beliefs and those with low conspiracy beliefs. As noted earlier, this might be due to a generally weak or indistinct visual stereotypes of how individuals who engage with conspiracy theories are expected to look. For instance, individuals with both low and high conspiracy beliefs may envision those who engage with conspiracy theories as a diverse group without a single, stereotypical appearance.

### The Role of Participants’ Political Orientation

Our findings from Study 1 also underscore the significant role of political orientation in shaping how individuals perceive those who believe in conspiracy theories. While participants across the political spectrum viewed conspiracy theory believers as less competent and were less willing to have contact with them, this trend was notably less pronounced among those with a far-right political leaning. In contrast, participants from the far left to the center perceived conspiracy believers as more Machiavellian and psychopathic, a perception not shared by right-leaning individuals. Given the rise in ideological polarization in the United States in recent decades (Boxell et al., 2022; Draca & Schwarz, 2021) and the propagation of conspiracy theories by prominent right-leaning media outlets (e.g., Motta et al., 2020), these findings may reflect why left-leaning individuals harbor more negative views toward conspiracy believers, viewing them as more manipulative and antisocial, an outlook not echoed by the political right, who may view them as more normalized.

We observed three-way interactions where participants on the far-right of the political spectrum perceived characters engaging with conspiracy theories (whether spreading, believing, or both) as warmer and were more willing to have contact with them, compared to participants with left-leaning orientations. The link between political orientation and conspiracy beliefs remains complex. While some research suggests that conservatives are more prone to conspiracy beliefs (van der Linden et al., 2021) other studies argue that such beliefs are common at both extremes of the political spectrum (Imhoff et al., 2022; Kunst, Gundersen, et al., 2024). Additionally, the connection between political orientation and conspiracy beliefs may vary based on the socio-political context and the nature of the conspiracy theories (Enders et al., 2023). Our findings indicate that in the United States, the political right is more receptive to individuals engaging with conspiracy theories, possibly viewing them less as a stigmatized group and more as “critical freethinkers” (Harambam & Aupers, 2017) questioning authority. This perception could explain their more favorable evaluations. Future research should explore how different social and national contexts, as well as specific conspiracy beliefs, influence the relationship between political orientation and perceptions of conspiracy engagement.

Although participants on the political right differed from those on the political left in their explicit evaluations of individuals engaging with conspiracy theories, findings from Study 2 suggest they do not differ in their mental representations. Once again, this may stem from the absence of a clear visual stereotype regarding how such individuals are expected to look.

### Social Implications

The processes observed in our studies may have significant social consequences for individuals who engage with conspiracy theories. Several traits measured in our studies, such as competence and warmth, are known to systematically predict attitudes, emotions, and behaviors toward individuals who exhibit these traits (Cuddy et al., 2007, 2008). For example, groups perceived as cold and incompetent often elicit emotions such as contempt and disgust, as well as active and passive behaviors like harm and neglect (Fiske et al., 2002). It is therefore likely that individuals engaging with conspiracy theories provoke similar emotional and behavioral responses from those who do not engage with such theories. Although our studies did not directly measure participants’ emotional sentiments, we found that individuals who engaged with conspiracy theories were generally evaluated more negatively across all dimensions. Moreover, participants expressed significantly less willingness to have contact with such individuals. Avoiding social interaction with disliked individuals can potentially undermine social cohesion and further exacerbate factual belief polarization, where citizens’ perceptions of reality diverge (Rekker & Harteveld, 2022).

Similarly, prior research has shown that individuals who believed Covid-19 conspiracy theories during the pandemic were more likely to experience social rejection (Van Prooijen et al., 2023), while non-believers reported fears of social exclusion when asked to endorse conspiracy theories on their social media profiles (Lantian et al., 2018). These fears seem not unfounded. Our findings from Study 1 indicate that individuals who spread conspiracy theories face the most severe negative evaluations, with participants expressing the least willingness to have contact with them. Strikingly, even those who themselves held strong conspiracy beliefs were negative toward having contact with such spreaders. Consequently, spreading conspiracy theories may carry the most unfavorable consequences for endorsing individuals. It is unsurprising, then, that conspiracy theories are typically shared within and remain confined to groups and communities that already believe in them (Metaxas & Finn, 2017; Sunstein & Vermeule, 2009).

Our findings also demonstrated that both conspiracy believers and spreaders were perceived as more willing to engage in conspiratorial plots themselves. Previous work suggests that one determinant of conspiracy beliefs is a projection of one’s own intentions onto others (see Douglas & Sutton, 2011). In other words, conspiracy believers believe others are conspiring because they expect others to act in a similar way to themselves. Our work takes this further, showing that people appear to hold an intuitive awareness of this association when evaluating conspiracy believers and spreaders. In contrast, those who hold stronger conspiracy beliefs themselves are less accurate in this intuition, revealing a potential bias that makes them more vulnerable to falling victim to conspiracies orchestrated by their own ingroup members (see also Biddlestone et al., 2022). More work is required to further understand the mechanisms of this process, but we suggest that an ingroup favoritism effect may be at play. That is, conspiracy believers may perceive other conspiracy believers as fellow crusaders in the benevolent fight against unjust plots. As a result, they may trust these individuals due to their shared goals. At the same time, these individuals appear unreflective and thus unaware of their group’s ironic higher willingness to engage in conspiratorial plots compared to the general public.

### Limitations and Future Directions

Previous research has linked conspiracy beliefs to feelings of exclusion (Biddlestone et al., 2025; Graeupner & Coman, 2017) and social isolation (Moulding et al., 2016). Building on this, our study shows that individuals are less inclined to engage with those who endorse conspiracy theories, with this reluctance co-occurring with negative perceptions of social traits (i.e., competence, morality, warmth) and dark personality characteristics (i.e., Machiavellianism, psychopathy). However, the desire for social contact is multifaceted, affected by various aspects of an individual’s identity, such as gender, age, profession, ethnicity, and other significant group identities. In our studies, we solely manipulated their engagement with conspiracy theories, not accounting for these other potential influences on social perceptions and willingness for contact. Notably, the gender of the characters did not impact their perception. Additionally, it is important to recognize that perceptions are shaped by complex bottom-up and top-down processes—an aspect we did not directly investigate. In perceiving and interpreting others’ attributes and behavior, bottom-up information from various sensory modalities is rapidly integrated with various top-down information sources such as stereotypes, cultural knowledge, and context (Freeman & Ambady, 2011). Furthermore, the type and quality of social relationships and cross-group membership (e.g., belonging to the same sports club) may further shape how individuals who engage with conspiracy theories are perceived. Future research could enrich our understanding by incorporating more detailed character descriptions or contextual factors to see how they influence perceptions of individuals who engage with conspiracy theories.

Across the two studies, we drew on three U.S. samples that were representative of key demographic variables (e.g., age, gender, ethnicity, political orientation, education). However, the specific dimensions on which each sample achieved quota-representativeness varied, which poses some limitations for generalizing the findings. Moreover, given that the prevalence of conspiracy beliefs and the nature of conspiracy theories vary internationally (Gundersen et al., 2023; Hornsey & Pearson, 2022; Imhoff et al., 2022), our results may not extend to different national contexts with distinct sociocultural and political environments. Additionally, our use of a broad definition for “conspiracy theory” without specifying particular theories limits our understanding of what participants envisioned regarding individuals engaging with such theories. Future research should explore how perceptions of individuals engaging with conspiracy theories differ across various contexts and incorporate specific conspiracy theories to gain a deeper insight into the distinctions in how individuals engaging with conspiracies are perceived across different sociocultural settings and in relation to particular beliefs. Moreover, future work should consider the role of plausibility, as individuals may respond differently to those who endorse conspiracy theories that appear more plausible versus those that are widely regarded as implausible.

In our studies, we explicitly described how the fictional individuals engaged with conspiracy theories. However, such detailed information may only be available in certain contexts. For example, people express their beliefs and support of conspiracy theories on social media (Kunst, Gundersen, et al., 2024). Consequently, we believe that our findings are most applicable within this specific context.

Finally, although our measure of contact willingness—an adapted version of the social distance scale (Bogardus, 1933)—is widely used to assess perceived social distance and prejudice (particularly toward out-groups; see Wark & Galliher, 2007), it remains unclear how well these findings translate to real-world behavior. Moreover, previous research indicates that perceived social distance is influenced by individual personality traits (e.g., Jonáš et al., 2023) and sociodemographic factors (Parrillo & Donoghue, 2005). Future studies could therefore explore how people actively distance themselves from individuals who engage with conspiracy theories (e.g., by unfollowing them on social media) and examine the extent to which other relevant factors shape these distancing behaviors.

## Conclusion

In conclusion, our studies demonstrate that believing in and spreading conspiracy theories exert independent and, at times, interactive effects on social perceptions and orientations. Individuals who engage with conspiracy theories are generally viewed more negatively than those who do not, being perceived as less competent, moral, and warm, while also being attributed to higher levels of narcissism, Machiavellianism, and psychopathy. Moreover, participants report lower willingness to have contact with such individuals and view them as more likely to engage in conspiratorial activities. Notably, *spreading* was the primary driver of effects on verbal descriptions in Study 1, whereas *believing* emerged as the main driver in Study 2 (mental representations). Furthermore, although Study 1 suggests that participants’ own conspiracy beliefs and political orientation shape their evaluations, these moderating effects did not replicate in the mental representations examined in Study 2. Together, these findings underscore the complex interplay of personal beliefs, political orientation, and social perceptions in shaping attitudes toward individuals who engage with conspiracy theories.

## Supplemental Material

sj-docx-1-qjp-10.1177_17470218251396952 – Supplemental material for The Dual Impact of Believing and Spreading Conspiracy Theories: Independent and Interactive Effects on Social Perceptions and Orientations Supplemental material, sj-docx-1-qjp-10.1177_17470218251396952 for The Dual Impact of Believing and Spreading Conspiracy Theories: Independent and Interactive Effects on Social Perceptions and Orientations by Aleksander B. Gundersen, Mikey Biddlestone and Jonas R. Kunst in Quarterly Journal of Experimental Psychology

## Notes

1. In the pre-registration, we termed this variable contact avoidance. However, we analyze the reversed scores here, therefore termed contact willingness.

2. Hypotheses H9 and H10 from the pre-registration were merged here because they represented inverse formulations of the same expected relationship between trait ratings and contact willingness.

3. In the pre-registration, this hypothesis specified “or” rather than “and/or.” We revised the wording to “and/or” to accurately reflect our prediction.

4. Although labeled “manipulative” in the pre-registration and questionnaire, we refer to it as “Machiavellianism” in the manuscript for consistency with Study 1 and given that it is a core feature of the construct.
