# Supplementary material for: The Dual Impact of Believing and Spreading Conspiracy Theories: Independent and Interactive Effects on Social Perceptions and Orientations
Source: Q J Exp Psychol (Hove). 2025 Nov 5;79(7):1884–915. doi: 10.1177/17470218251396952 (PMC13310293; doi:10.1177/17470218251396952)
Supplement: sj-docx-1-qjp-10.1177_17470218251396952 – Supplemental material for The Dual Impact of Believing and Spreading Conspiracy Theories: Independent and Interactive Effects on Social Perceptions and Orientations [file sj-docx-1-qjp-10.1177_17470218251396952.docx]

Supplementary Materials for:

**The Dual Impact of Believing and Spreading Conspiracy Theories: Independent and Interactive Effects on Social Perceptions and Orientations**

Aleksander B. Gundersen^1^, Mikey Biddlestone^2^, and Jonas R. Kunst^3,1^

^1^ Department of Psychology, University of Oslo, Norway

^2^ School of Psychology, University of Kent, United Kingdom

^3^ BI Norwegian Business School, Norway

**Author Note**

Correspondence concerning this article should be addressed to Aleksander B. Gundersen, Department of Psychology, University of Oslo, Postboks 1094 Blindern, 0317 Oslo, Norway. Email: a.b.gundersen@psykologi.uio.no. Web link to data and materials: https://osf.io/k34qm/?view_only=d9e787a4f05f4115b6f6b8f10f4da4ad (Gundersen, 2025S).

**Study 1**

| **Table S1** | | |
| --- | --- | --- |
| *Sample Demographic Characteristics* | | |
| Variable | *n* | % |
| Gender |  |  |
| Male | 200 | 47.5 |
| Female | 212 | 50.7 |
| Missing | 6 | 1.4 |
| Ethnicity |  |  |
| White/Caucasian | 329 | 78.7 |
| African American | 40 | 9.6 |
| Hispanic | 10 | 2.4 |
| Asian | 21 | 5.0 |
| Arab | 2 | 0.5 |
| Other | 16 | 3.8 |
| Region of residence |  |  |
| City | 307 | 73.4 |
| Countryside | 111 | 26.6 |
| Political party affiliation |  |  |
| Republican | 124 | 29.7 |
| Democrat | 133 | 31.8 |
| Independent | 156 | 37.3 |
| Other | 3 | 0.7 |
| No preference | 2 | 0.5 |
| Highest level of education |  |  |
| Less than high school degree | 2 | 0.5 |
| High school graduate | 67 | 16.0 |
| Some college but no degree | 77 | 18.4 |
| Associate degree in college | 46 | 11.0 |
| Bachelor’s degree in college | 151 | 36.1 |
| Master’s degree | 61 | 14.6 |
| Doctoral degree | 8 | 1.9 |
| Professional degree | 6 | 1.4 |
| Household income per year before taxes |  |  |
| Less than $15,000 | 28 | 6.7 |
| $15,000 – $24,999 | 33 | 7.9 |
| $25,000 – $34,999 | 32 | 7.7 |
| $35,000 – $49,999 | 70 | 16.7 |
| $50,000 – $74,999 | 80 | 19.1 |
| $75,000 – $99,999 | 68 | 16.3 |
| $100,000 – $149,999 | 72 | 17.2 |
| $150,000 or more | 35 | 8.4 |

| **Table S2** | | | | |
| --- | --- | --- | --- | --- |
| *Residual Diagnostics for Multi-Level Linear Models Presented in Table 1 in the Main Document* | | | | |
| Models | *p*  Dispersion Test | *p*  Outlier Test | *p*  KS Test |  |
| Competence | .576 | **< .001** | **< .001** |  |
| Morality | .368 | .118 | **< .001** |  |
| Warmth | .624 | **.040** | **.002** |  |
| Contact Willingness | .240 | > .999 | **< .001** |  |
| Machiavellianism | .648 | .626 | **< .001** |  |
| Narcissism | .656 | .770 | **<. 001** |  |
| Psychopathy | .248 | .380 | **.013** |  |
| Conspiracy Intentions | .424 | **< .001** | **< .001** |  |
| *Note.* KS = Kolmogorov-Smirnov | | | | |

| **Table S3** | | | | | | | | | | | | |
| --- | --- | --- | --- | --- | --- | --- | --- | --- | --- | --- | --- | --- |
| *Results of Multi-Level Linear Models: Examining the Fixed Effects of Spread and Believe, and Their Interaction on Perceived Competence, Morality, and Warmth, with Control for Random Intercepts and Effects* | | | | | | | | | | | | |
|  | Competence | | | | Morality | | | | Warmth | | | |
| Effect | *B*  [95% CI] | *SE* | *t* | *df* | *B*  [95% CI] | *SE* | *t* | *df* | *B*  [95% CI] | *SE* | *t* | *df* |
| Fixed effects |  |  |  |  |  |  |  |  |  |  |  |  |
| (Intercept) | 5.26***  [5.15, 5.37] | 0.06 | 94.05 | 292.56 | 5.35***  [5.23, 5.46] | 0.06 | 89.74 | 331.07 | 5.12***  [5.00, 5.24] | 0.06 | 83.61 | 173.10 |
| Spread | -1.75***  [-1.90, -1.60] | 0.07 | -23.39 | 562.72 | -3.00***  [-3.16, -2.84] | 0.08 | -36.66 | 573.00 | -2.04***  [-2.19, 1.90] | 0.07 | -27.39 | 557.07 |
| Believe | -0.84***  [-0.98, -0.69] | 0.07 | -11.30 | 541.16 | -0.81***  [-0.97, -0.66] | 0.08 | -10.29 | 554.07 | -0.46***  [-0.59, -0.34] | 0.06 | -7.24 | 574.55 |
| Spread * Believe | 0.91***  [0.80, 1.01] | 0.06 | 16.31 | 2656.13 | 1.97***  [1.84, 2.09] | 0.06 | 31.30 | 2708.13 | 0.97***  [0.86, 1.07] | 0.05 | 17.94 | 2671.71 |
| Random Effects | Variance (*SD*) | | | | Variance (*SD*) | | | | Variance (*SD*) | | | |
| Participants intercept | 0.95 (0.98) | | | | 1.07 (1.04) | | | | 1.08 (1.04) | | | |
| Spread | 1.71 (1.31) | | | | 2.00 (1.42) | | | | 1.73 (1.31) | | | |
| Believe | 1.70 (1.30) | | | | 1.86 (1.36) | | | | 1.15 (1.07) | | | |
| Trial | 0.00 (0.04) | | | | 0.00 (0.03) | | | | 0.00 (0.06) | | | |
| *R^2^* |  |  |  |  |  |  |  |  |  |  |  |  |
| Conditional | .85 | | | | .86 | | | | .86 | | | |
| Marginal | .19 | | | | .35 | | | | .26 | | | |
| *Note.* CI = confidence interval.  ****p_Holm_* < .001. | | | | | | | | | | | | |

| **Table S4** | | | | | | | | | | | | |
| --- | --- | --- | --- | --- | --- | --- | --- | --- | --- | --- | --- | --- |
| *Results of Multi-Level Linear Models: Examining the Fixed Effects of Spread and Believe, and Their Interaction on Perceived Machiavellianism, Narcissism, and Psychopathy, with Control for Random Intercepts and Effects* | | | | | | | | | | | | |
|  | Machiavellianism | | | | Narcissism | | | | Psychopathy | | | |
| Effect | *B*  [95% CI] | *SE* | *t* | *df* | *B*  [95% CI] | *SE* | *t* | *df* | *B*  [95% CI] | *SE* | *t* | *df* |
| Fixed effects |  |  |  |  |  |  |  |  |  |  |  |  |
| (Intercept) | 2.71***  [2.59, 2.83] | 0.06 | 44.11 | 343.68 | 3.72***  [3.61, 3.84] | 0.06 | 62.90 | 471.84 | 2.63***  [2.51, 2.75] | 0.06 | 43.05 | 193.33 |
| Spread | 2.78***  [2.63, 2.94] | 0.08 | 34.68 | 609.48 | 1.82***  [1.68, 1.97] | 0.07 | 24.54 | 587.97 | 2.64***  [2.49, 2.79] | 0.08 | 34.20 | 607.46 |
| Believe | 0.27***  [0.13, 0.42] | 0.07 | 3.68 | 618.62 | -0.03  [-0.15, 0.08] | 0.06 | -0.54 | 675.22 | 0.49***  [0.35, 0.63] | 0.07 | 6.94 | 618.66 |
| Spread * Believe | -1.25***  [-1.38, -1.12] | 0.07 | -18.52 | 2837.97 | -0.44***  [-0.55, -0.32] | 0.06 | -7.40 | 2833.24 | -1.31***  [-1.43, -1.18] | 0.06 | -20.31 | 2822.65 |
| Random Effects | Variance (*SD*) | | | | Variance (*SD*) | | | | Variance (*SD*) | | | |
| Participants intercept | 1.12 (1.06) | | | | 1.12 (1.06) | | | | 1.00 (1.00) | | | |
| Spread | 1.78 (1.34) | | | | 1.60 (1.27) | | | | 1.66 (1.29) | | | |
| Believe | 1.43 (1.19) | | | | 0.76 (0.87) | | | | 1.29 (1.14) | | | |
| Trial | 0.00 (0.02) | | | | 0.00 (0.00) | | | | 0.00 (0.06) | | | |
| *R^2^* |  |  |  |  |  |  |  |  |  |  |  |  |
| Conditional | .81 | | | | .79 | | | | .81 | | | |
| Marginal | .39 | | | | .29 | | | | .37 | | | |
| *Note.* CI = confidence interval.  ****p_Holm_* < .001. | | | | | | | | | | | | |

| **Table S5** | | | | | | | | | | | | |
| --- | --- | --- | --- | --- | --- | --- | --- | --- | --- | --- | --- | --- |
| *Results of Multi-Level Linear Models: Examining the Fixed Effects of Spread and Believe, and Their Interaction on Contact Willingness, and Conspiracy Intentions, with Control for Random Intercepts and Effects* | | | | | | | | | | | | |
|  | Contact Willingness | | | | Conspiracy Intentions | | | |  | | | |
| Effect | *B*  [95% CI] | *SE* | *t* | *df* | *B*  [95% CI] | *SE* | *t* | *df* |  |  |  |  |
| Fixed effects |  |  |  |  |  |  |  |  |  |  |  |  |
| (Intercept) | 5.39***  [5.27, 5.50] | 0.06 | 93.19 | 147.23 | 2.37***  [2.23, 2.50] | 0.07 | 33.31 | 206.98 |  |  |  |  |
| Spread | -2.73***  [-2.89, -2.57] | 0.08 | -34.43 | 544.01 | 2.18***  [2.02, 2.34] | 0.08 | 26.89 | 617.61 |  |  |  |  |
| Believe | -0.86***  [-1.01, -0.70] | 0.08 | -10.97 | 525.57 | 0.40***  [0.24, 0.56] | 0.08 | 4.78 | 571.52 |  |  |  |  |
| Spread * Believe | 1.42***  [1.31, 1.53] | 0.06 | 25.28 | 2647.58 | -1.31***  [-1.45, -1.18] | 0.07 | -19.11 | 2748.33 |  |  |  |  |
| Random Effects | Variance (*SD*) | | | | Variance (*SD*) | | | |  | | | |
| Participants intercept | 0.98 (0.94) | | | | 1.44 (1.20) | | | |  | | | |
| Spread | 1.99 (1.41) | | | | 1.79 (1.34) | | | |  | | | |
| Believe | 1.94 (1.39) | | | | 2.03 (1.42) | | | |  | | | |
| Trial | 0.00 (0.06) | | | | 0.01 (0.06) | | | |  | | | |
| *R^2^* |  |  |  |  |  |  |  |  |  |  |  |  |
| Conditional | .88 | | | | .81 | | | |  | | | |
| Marginal | .34 | | | | .22 | | | |  | | | |
| *Note.* CI = confidence interval.  ****p_Holm_* < .001. | | | | | | | | | | | | |

**Section S1: Model Results Expanded - Fixed Effects and Pairwise Comparisons**

With perceived competence (H1) as the outcome measure, fixed effects revealed that spread, *B* = -1.75, 95% CI = [-1.90, -1.60], *SE* = .07, *t*(562.70) = -23.39, *p* < .001, believe, *B* = -0.84, 95% CI = [-0.98, -0.69], *SE* = .07, *t*(541.17) = -11.30, *p* < .001, and the two-way interaction between spread and believe, *B* = 0.91, 95% CI = [0.80, 1.01], *SE* = .06, *t*(2656.13) = 16.31, *p* < .001, all significantly predicted perceived competence of the fictitious characters.
 Follow-up pairwise comparisons using the Holm-method for significance adjustment indicated that the fictitious characters that neither spread nor believed in conspiracy theories were perceived as significantly more competent (*M* = 5.26, 95% CI = [5.15, 5.37], *SE* = .06, *p* < .001) than those who believe but not spread (*M* = 4.42, 95% CI = [4.28, 4.57], *SE* = .07, *d* = 1.34, *p* < .001), those who spread but not believe (*M* = 3.51, 95% CI = [3.37, 3.65], *SE* = .07, *d* = 2.81, *p* < .001), and those who both spread and believe conspiracy theories (*M* = 3.58, 95% CI = [3.42, 3.75], *SE* = .08, *d* = 2.69, *p* < .001). The fictitious characters that believe but do not spread conspiracy theories were also perceived as significantly more competent compared to those who only spread (*d* = 1.46, *p* < .001), and those who both spread and believe (*d* = 1.35, *p* < .001). No significant difference was found between those who only spread and those who both spread and believe (*d* = 0.11, *p* = .351). In sum, partially supporting our hypothesis, the fictitious characters that were perceived as most competent were those who: neither believe nor spread > believe but do not spread > spread and believe ≥ spread but do not believe.
 With perceived morality (H2) as the outcome measure, fixed effects revealed that spread, *B* = -3.00, 95% CI = [-3.16, -2.84], *SE* = .08, *t*(573.00) = -36.66, *p* < .001, believe, *B* = -0.81, 95% CI = [-0.97, -0.66], *SE* = .08, *t*(554.07) = -10.29, *p* < .001, and the two-way interaction between spread and believe, *B* = 1.97, 95% CI = [1.84, 2.09], *SE* = .06, *t*(2708.13) = 31.30, *p* < .001, all significantly predicted perceived morality of the fictitious characters.
 Follow-up pairwise comparisons using the Holm-method for significance adjustment indicated that the fictitious characters that neither spread nor believed in conspiracy theories were perceived as significantly more moral (*M* = 5.35, 95% CI = [5.23, 5.46], *SE* = .06, *p* < .001) than those who believe but not spread (*M* = 4.53, 95% CI = [4.39, 4.67], *SE* = .07, *d* = 1.14, *p* < .001), those who spread but not believe (*M* = 2.34, 95% CI = [2.21, 2.48], *SE* = .07, *d* = 4.21, *p* < .001), and those who both spread and believe conspiracy theories (*M* = 3.50, 95% CI = [3.33, 3.66], *SE* = .08, *d* = 2.59, *p* < .001). The fictitious characters that believe but do not spread conspiracy theories were also perceived as significantly more moral compared to those who only spread (*d* = 2.19, *p* < .001), and those who both spread and believe (*d* = 1.45, *p* < .001). Those who both believe and spread conspiracy theories were also perceived as significantly more moral than those who only spread (*d* = 1.61, *p* < .001). In sum, partially supporting our hypothesis, the fictitious characters that were perceived as most moral were those who: neither believe nor spread > believe but do not spread > spread and believe > spread but do not believe.
 With perceived warmth (H3) as the outcome measure, fixed effects revealed that spread, *B* = -2.04, 95% CI = [-2.19, -1.90], *SE* = .07, *t*(557.09) = -27.39, *p* < .001, believe, *B* = -0.46, 95% CI =[-0.59, -0.34], *SE* = .06, *t*(574.52) = -7.24, *p* < .001, and the two-way interaction between spread and believe, *B* =0.97, 95% CI = [0.86, 1.07], *SE* = .05, *t*(2671.73) = 17.94, *p* < .001, all significantly predicted perceived warmth of the fictitious characters.
 Follow-up pairwise comparisons using the Holm-method for significance adjustment indicated that the fictitious characters that neither spread nor believed in conspiracy theories were perceived as significantly warmer (*M* = 5.12, 95% CI = [5.00, 5.24], *SE* = .06, *p* < .001) than those who believe but not spread (*M* = 4.66, 95% CI = [4.53, 4.79], *SE* = .06, *d* = 0.76, *p* < .001), those who spread but not believe (*M* = 3.08, 95% CI = [2.94, 3.22], *SE* = .07, *d* = 3.67, *p* < .001), and those who both spread and believe conspiracy theories (*M* = 3.58, 95% CI = [3.43, 3.74], *SE* = .08, *d* = 2.53, *p* < .001). The fictitious characters that believe but do not spread conspiracy theories were also perceived as significantly warmer compared to those who only spread (*d* = 2.61, *p* < .001), and those who both spread and believe (*d* = 1.77, *p* < .001). Those who both believe and spread conspiracy theories were also perceived as significantly warmer than those who only spread (*d* = 0.83, *p* < .001). In sum, partially supporting our hypothesis, the fictitious characters that were perceived as the warmest were those who: neither believe nor spread > believe but do not spread > spread and believe > spread but do not believe.
 With perceived Machiavellianism (H4) as the outcome measure, fixed effects revealed that spread, *B* = 2.78, 95% CI = [2.63, 2.94], *SE* = .08, *t*(609.48) = 34.68, *p* < .001, believe, *B* = 0.27, 95% CI =[0.13, 0.42], *SE* = .07, *t*(618.62) = 3.68, *p* < .001, and the two-way interaction between spread and believe, *B* = -1.25, 95% CI = [-1.38, -1.12], *SE* = .07, *t*(2837.97) = -18.52, *p* < .001, all significantly predicted perceived Machiavellianism of the fictitious characters.
 Follow-up pairwise comparisons using the Holm-method for significance adjustment indicated that the fictitious characters that neither spread nor believed in conspiracy theories were perceived as significantly less Machiavellian (*M* = 2.71, 95% CI = [2.59, 2.83], *SE* = .06, *p* < .001) than those who believe but not spread (*M* = 2.99, 95% CI = [2.84, 3.14], *SE* = .08, *d* = -0.34, *p* < .001), those who spread but not believe (*M* = 5.50, 95% CI = [5.39, 5.61], *SE* = .06, *d* = -3.53, *p* < .001), and those who both spread and believe conspiracy theories (*M* = 4.52, 95% CI = [4.38, 4.66], *SE* = .07, *d* = -2.29, *p* < .001). The fictitious characters that believe but do not spread conspiracy theories were also perceived as significantly less Machiavellian compared to those who only spread (*d* = -3.18, *p* < .001), and those who both spread and believe (*d* = -1.94, *p* < .001). Those who both believe and spread conspiracy theories were also perceived as significantly less Machiavellian than those who only spread (*d* = -1.23, *p* < .001). In sum, partially supporting our hypothesis, the fictitious characters that were perceived as the most Machiavellian were those who: spread but do not believe > spread and believe > believe but do not spread > neither believe nor spread.
 With perceived narcissism (H5) as the outcome measure, fixed effects revealed that spread, *B* = 1.82, 95% CI = [1.68, 1.97], *SE* = .07, *t*(587.97) = 24.54, *p* < .001, and the two-way interaction between spread and believe, *B* = -0.44, 95% CI = [-0.55, -0.32], *SE* = .06, *t*(2833.24) = -7.40, *p* < .001, significantly predicted perceived narcissism of the fictitious characters. The fixed effect of spread on perceived narcissism was not significant, *B* = -0.03, 95% CI =[-0.15, 0.08], *SE* = .06, *t*(675.22) = -0.54, *p* = .586.
 Follow-up pairwise comparisons using the Holm-method for significance adjustment indicated that the fictitious characters that neither spread nor believed in conspiracy theories were perceived as significantly less narcissistic (*M* = 3.72, 95% CI = [3.61, 3.84], *SE* = .06, *p* < .001) than those who spread but not believe (*M* = 5.55, 95% CI = [5.45, 5.64], *SE* = .05, *d* = -2.61, *p* < .001), and those who both spread and believe conspiracy theories (*M* = 5.07, 95% CI = [4.96, 5.19], *SE* = .06, *d* = -1.93, *p* < .001), but not compared to those who believe but not spread (*M* = 3.69, 95% CI = [3.55, 3.83], *SE* = .07, *d* = 0.04, *p* = .586.) The fictitious characters that believe but do not spread conspiracy theories were perceived as significantly less narcissistic compared to those who only spread (*d* = -2.65, *p* < .001), and those who both spread and believe (*d* = -1.98, *p* < .001). Those who both believe and spread conspiracy theories were also perceived as significantly less narcissistic than those who only spread (*d* = -0.67, *p* < .001). In sum, partially supporting our hypothesis, the fictitious characters that were perceived as the most narcissistic were those who: spread but do not believe > spread and believe > neither believe nor spread ≥ believe but do not spread.
 With perceived psychopathy (H6) as the outcome measure, fixed effects revealed that spread, *B* = 2.64, 95% CI = [2.49, 2.79], *SE* = .08, *t*(607.46) = 34.20, *p* < .001, believe, *B* = 0.49, 95% CI =[0.35, 0.63], *SE* = .07, *t*(618.66) = 6.94, *p* < .001, and the two-way interaction between spread and believe, *B* = -1.31, 95% CI = [-1.43, -1.18], *SE* = .06, *t*(2822.65) = -20.31, *p* < .001, all significantly predicted perceived psychopathy of the fictitious characters.
 Follow-up pairwise comparisons using the Holm-method for significance adjustment indicated that the fictitious characters that neither spread nor believed in conspiracy theories were perceived as significantly less psychopathic (*M* = 2.63, 95% CI = [2.51, 2.75], *SE* = .06, *p* < .001) than those who believe but not spread (*M* = 3.13, 95% CI = [2.98, 3.27], *SE* = .07, *d* = -0.65, *p* < .001), those who spread but not believe (*M* = 5.27, 95% CI = [5.16, 5.38], *SE* = .06, *d* = -3.51, *p* < .001), and those who both spread and believe conspiracy theories (*M* = 4.46, 95% CI = [4.32, 4.59], *SE* = .07, *d* = -2.42, *p* < .001). The fictitious characters that believe but do not spread conspiracy theories were also perceived as significantly less psychopathic compared to those who only spread (*d* = -2.85, *p* < .001), and those who both spread and believe (*d* = -1.77, *p* < .001). Those who both believe and spread conspiracy theories were also perceived as significantly less psychopathic than those who only spread (*d* = -1.08, *p* < .001). In sum, partially supporting our hypothesis, the fictitious characters that were perceived as the most psychopathic were those who: spread but do not believe > spread and believe > believe but do not spread > neither believe nor spread.
 With contact willingness (H7) as the outcome measure, fixed effects revealed that spread, *B* = -2.73, 95% CI = [-2.89, -2.57], *SE* = .08, *t*(544.04) = -34.43, *p* < .001, believe, *B* = -0.86, 95% CI =[-1.01, -0.70], *SE* = .08, *t*(525.56) = -10.97, *p* < .001, and the two-way interaction between spread and believe, *B* = 1.42, 95% CI = [1.31, 1.53], *SE* = .06, *t*(2647.57) = 25.28, *p* < .001, all significantly predicted participants’ willingness to have contact with the fictitious characters.
 Follow-up pairwise comparisons using the Holm-method for significance adjustment indicated that the fictitious characters that neither spread nor believed in conspiracy theories were the ones that the participants were more willing to have contact with (*M* = 5.39, 95% CI = [5.27, 5.50], *SE* = .06, *p* < .001) than those who believe but not spread (*M* = 4.53, 95% CI = [4.38, 4.68], *SE* = .07, *d* = 1.35, *p* < .001), those who spread but not believe (*M* = 2.66, 95% CI = [2.52, 2.79], *SE* = .07, *d* = 4.32, *p* < .001), and those who both spread and believe conspiracy theories (*M* = 3.22, 95% CI = [3.04, 3.40], *SE* = .09, *d* = 3.43, *p* < .001). The fictitious characters that believe but do not spread conspiracy theories were also the ones that the participants were more willing to have contact with compared to those who only spread (*d* = 2.97, *p* < .001), and those who both spread and believe (*d* = 2.07, *p* < .001). Those who both believe and spread conspiracy theories were also the ones that the participants were more willing to have contact with than those who only spread (*d* = 0.89, *p* < .001). In sum, partially supporting our hypothesis, the fictitious characters that the participants were more willing to have contact with were those who: neither believe nor spread > believe but do not spread > spread and believe > spread but do not believe.
 With perceived conspiracy intentions (H8) as the outcome measure, fixed effects revealed that spread, *B* = 2.18, 95% CI = [2.02, 2.34], *SE* = .08, *t*(617.57) = 26.88, *p* < .001, believe, *B* = 0.40, 95% CI =[0.24, 0.56], *SE* = .08, *t*(571.57) = 4.78, *p* < .001, and the two-way interaction between spread and believe, *B* = -1.31, 95% CI = [-1.45, -1.18], *SE* = .07, *t*(2748.34) = -19.11, *p* < .001, all significantly predicted perceived conspiracy intentions of the fictitious characters.
 Follow-up pairwise comparisons using the Holm-method for significance adjustment indicated that the fictitious characters that neither spread nor believed in conspiracy theories were perceived as having significantly less intentions to conspire (*M* = 2.37, 95% CI = [2.23, 2.50], *SE* = .07, *p* < .001) than those who believe but not spread (*M* = 2.77, 95% CI = [2.61, 2.92], *SE* = .08, *d* = -0.51, *p* < .001), those who spread but not believe (*M* = 4.55, 95% CI = [4.40, 4.69], *SE* = .07, *d* = -2.78, *p* < .001), and those who both spread and believe conspiracy theories (*M* = 3.63, 95% CI = [3.46. 3.80], *SE* = .09, *d* = -1.62, *p* < .001). The fictitious characters that believe but do not spread conspiracy theories were also perceived as having significantly less intentions to conspiracy compared to those who only spread (*d* = -2.26, *p* < .001), and those who both spread and believe (*d* = -1.11, *p* < .001). Those who both believe and spread conspiracy theories were also perceived as having significantly less intentions to conspire than those who only spread (*d* = -1.16, *p* < .001). In sum, partially supporting our hypothesis, the fictitious characters that were perceived as having the greatest intentions to conspire were those who: spread but do not believe > spread and believe > believe but do not spread > neither believe nor spread.

| **Table S6** | | | | | | | | | | | | | | | |
| --- | --- | --- | --- | --- | --- | --- | --- | --- | --- | --- | --- | --- | --- | --- | --- |
| *Results of Multi-Level Linear Models: Examining the Fixed Effects of Spread and Believe on Dependent Variables, with Control for Random Intercepts and Effects* | | | | | | | | | | | | | | | |
|  | Competence | | | | | Morality | | | | | Warmth | | | | |
| Effect | *B*  [95% CI] | *SE* | *t* | *df* | *p* | *B*  [95% CI] | *SE* | *t* | *df* | *p* | *B*  [95% CI] | *SE* | *t* | *df* | *p* |
| Fixed effects |  |  |  |  |  |  |  |  |  |  |  |  |  |  |  |
| (Intercept) | 4.19  [4.10, 4.29] | 0.05 | 83.50 | 237.97 | < .001 | 3.93  [3.84, 4.02] | 0.05 | 83.57 | 221.84 | < .001 | 4.11  [4.01, 4.21] | 0.05 | 81.20 | 97.37 | < .001 |
| Spread | -1.30  [-1.43, -1.16] | 0.07 | -18.80 | 414.48 | < .001 | -2.02  [-2.17, -1.87] | 0.08 | -26.87 | 414.15 | < .001 | -1.56  [-1.69, -1.42] | 0.07 | -22.60 | 414.99 | < .001 |
| Believe | -0.38  [-0.52, -0.25] | 0.07 | -5.57 | 412.82 | < .001 | 0.17  [0.03, 0.31] | 0.07 | 2.34 | 407.96 | .012 | 0.02  [-0.09, 0.14] | 0.06 | 0.37 | 406.82 | .711 |
| Spread * Believe | 0.91  [0.80, 1.01] | 0.06 | 16.31 | 2656.14 | < .001 | 1.97  [1.84, 2.09] | 0.06 | 31.30 | 2708.13 | < .001 | 0.97  [0.86, 1.07] | 0.05 | 17.94 | 2671.72 | < .001 |
| Random Effects | Variance (*SD*) | | | | | Variance (*SD*) | | | | | Variance (*SD*) | | | | |
| Participants intercept | 0.92 (0.96) | | | | | 0.80 (0.89) | | | | | 0.79 (0.89) | | | | |
| Spread | 1.71 (1.31) | | | | | 2.00 (1.42) | | | | | 1.73 (1.31) | | | | |
| Believe | 1.70 (1.30) | | | | | 1.86 (1.36) | | | | | 1.15 (1.07) | | | | |
| Trial | 0.00 (0.04) | | | | | 0.00 (0.71) | | | | | 0.00 (0.06) | | | | |
| *R^2^* |  |  |  |  |  |  |  |  |  |  |  |  |  |  |  |
| Conditional | .85 | | | | | .86 | | | | | .85 | | | | |
| Marginal | .19 | | | | | .35 | | | | | .26 | | | | |
| *Note.* CI = confidence interval. Variables are effect coded. | | | | | | | | | | | | | | | |

| **Table S7** | | | | | | | | | | | | | | | |
| --- | --- | --- | --- | --- | --- | --- | --- | --- | --- | --- | --- | --- | --- | --- | --- |
| *Results of Multi-Level Linear Models: Examining the Fixed Effects of Spread and Believe on Dependent Variables, with Control for Random Intercepts and Effects* | | | | | | | | | | | | | | | |
|  | Machiavellianism | | | | | Narcissism | | | | | Psychopathy | | | | |
| Effect | *B*  [95% CI] | *SE* | *t* | *df* | *p* | *B*  [95% CI] | *SE* | *t* | *df* | *p* | *B*  [95% CI] | *SE* | *t* | *df* | *p* |
| Fixed effects |  |  |  |  |  |  |  |  |  |  |  |  |  |  |  |
| (Intercept) | 3.93  [3.85, 4.01] | 0.04 | 95.78 | 147.07 | < .001 | 4.51  [4.43, 4.58] | 0.04 | 117.11 | 412.62 | < .001 | 3.87  [3.79, 3.96] | 0.04 | 89.22 | 64.68 | < .001 |
| Spread | 2.16  [2.02, 2.30] | 0.07 | 29.79 | 415.61 | < .001 | 1.60  [1.47, 1.74] | 0.07 | 23.65 | 416.18 | < .001 | 1.98  [1.85, 2.12] | 0.07 | 28.45 | 416.32 | < .001 |
| Believe | -0.35  [-0.48, -0.22] | 0.07 | -5.28 | 409.80 | < .001 | -0.25  [-0.35, -0.15] | 0.05 | -4.94 | 404.97 | < .001 | -0.16  [-0.29, -0.04] | 0.06 | -2.56 | 409.13 | .011 |
| Spread * Believe | -1.25  [-1.38, -1.12] | 0.07 | -18.52 | 2837.97 | < .001 | -0.44  [-0.55, -0.32] | 0.06 | -7.40 | 2833.24 | < .001 | -1.31  [-1.43, -1.18] | 0.06 | -20.31 | 2822.65 | < .001 |
| Random Effects | Variance (*SD*) | | | | | Variance (*SD*) | | | | | Variance (*SD*) | | | | |
| Participants intercept | 0.57 (0.75) | | | | | 0.54 (0.73) | | | | | 0.53 (0.73) | | | | |
| Spread | 1.78 (1.34) | | | | | 1.60 (1.27) | | | | | 1.66 (1.29) | | | | |
| Believe | 1.43 (1.19) | | | | | 0.76 (0.87) | | | | | 1.29 (1.14) | | | | |
| Trial | 0.00 (0.02) | | | | | 0.00 (0.00) | | | | | 0.00 (0.06) | | | | |
| *R^2^* |  |  |  |  |  |  |  |  |  |  |  |  |  |  |  |
| Conditional | .81 | | | | | .79 | | | | | .81 | | | | |
| Marginal | .39 | | | | | .29 | | | | | .37 | | | | |
| *Note.* CI = confidence interval. Variables are effect coded. | | | | | | | | | | | | | | | |

| **Table S8** | | | | | | | | | | | | | | | |
| --- | --- | --- | --- | --- | --- | --- | --- | --- | --- | --- | --- | --- | --- | --- | --- |
| *Results of Multi-Level Linear Models: Examining the Fixed Effects of Spread and Believe on Dependent Variables, with Control for Random Intercepts and Effects* | | | | | | | | | | | | | | | |
|  | Conspiracy Intentions | | | | | Contact Willingness | | | | |  | | | | |
| Effect | *B*  [95% CI] | *SE* | *t* | *df* | *p* | *B*  [95% CI] | *SE* | *t* | *df* | *p* |  |  |  |  |  |
| Fixed effects |  |  |  |  |  |  |  |  |  |  |  |  |  |  |  |
| (Intercept) | 3.33  [3.22, 3.43] | 0.05 | 61.28 | 93.36 | < .001 | 3.95  [3.85, 4.05] | 0.05 | 78.11 | 96.28 | < .001 |  |  |  |  |  |
| Spread | 1.52  [1.38, 1.67] | 0.07 | 20.94 | 413.56 | < .001 | -2.02  [-2.16, -1.88] | 0.07 | -27.38 | 413.39 | < .001 |  |  |  |  |  |
| Believe | -0.26  [-0.41, -0.11] | 0.07 | -3.35 | 410.63 | < .001 | -0.15  [-0.29, -0.00] | 0.07 | -2.00 | 409.19 | .047 |  |  |  |  |  |
| Spread * Believe | -1.31  [-1.45, -1.18] | 0.07 | -19.11 | 2748.33 | < .001 | 1.42  [1.31, 1.53] | 0.06 | 25.28 | 2647.57 | < .001 |  |  |  |  |  |
| Random Effects | Variance (*SD*) | | | | | Variance (*SD*) | | | | |  | | | | |
| Participants intercept | 0.90 (0.95) | | | | | 0.78 (0.89) | | | | |  | | | | |
| Spread | 1.79 (1.34) | | | | | 1.99 (1.41) | | | | |  | | | | |
| Believe | 2.03 (1.42) | | | | | 1.94 (1.39) | | | | |  | | | | |
| Trial | 0.00 (0.06) | | | | | 0.00 (0.63) | | | | |  | | | | |
| *R^2^* |  |  |  |  |  |  |  |  |  |  |  |  |  |  |  |
| Conditional | .81 | | | | | .88 | | | | |  | | | | |
| Marginal | .22 | | | | | .34 | | | | |  | | | | |
| *Note.* CI = confidence interval. Variables are effect coded. | | | | | | | | | | | | | | | |

| **Table S9** | | | | | | | |
| --- | --- | --- | --- | --- | --- | --- | --- |
| *Results of Multi-Level Linear Models: Testing Effects of Individual Difference Traits on Contact Willingness With the Fictitious Characters* | | | | | | | |
| Effect | *B* | 95% CI | | *SE* | *t* | *df* | *p_Holm_* |
|  |  | *LL* | *UL* |  |  |  |  |
| Fixed effects |  |  |  |  |  |  |  |
| (Intercept) | 2.55 | 2.34 | 2.76 | 0.11 | 23.63 | 3344 | < .001 |
| Competence | 0.14 | 0.10 | 0.18 | 0.02 | 7.52 | 3344 | < .001 |
| Warmth | 0.24 | 0.20 | 0.28 | 0.02 | 10.78 | 3344 | < .001 |
| Morality | 0.30 | 0.26 | 0.34 | 0.02 | 15.84 | 3344 | < .001 |
| Machiavellianism | -0.11 | -0.15 | -0.07 | 0.02 | -5.41 | 3344 | < .001 |
| Narcissism | -0.03 | -0.06 | -0.00 | 0.02 | -2.18 | 3344 | .029 |
| Psychopathy | -0.13 | -0.17 | -0.09 | 0.02 | -6.49 | 3344 | < .001 |
| Conspiracy intentions | -0.08 | -0.10 | -0.05 | 0.01 | -5.63 | 3344 | < .001 |
| Random effects | Variance (*SD*) | |  |  |  |  |  |
| Trial | 0.00 (0.00) | |  |  |  |  |  |
| *R^2^* |  |  |  |  |  |  |  |
| Conditional | .74 |  |  |  |  |  |  |
| Marginal | .74 |  |  |  |  |  |  |
| *Note.* CI = confidence interval; *LL* = lower limit; *UL* = upper limit. | | | | | | | |

| **Table S10** | | | | |
| --- | --- | --- | --- | --- |
| *Residual Diagnostics for Multi-Level Linear Models Presented in Table 3 in the Main Document* | | | | |
| Models | *p*  Dispersion Test | *p*  Outlier Test | *p*  KS Test |  |
| Competence | .496 | **< .001** | .169 |  |
| Morality | .144 | .149 | **< .001** |  |
| Warmth | .472 | **.011** | **.030** |  |
| Contact Willingness | .152 | .436 | **.005** |  |
| Machiavellianism | .496 | .495 | **< .001** |  |
| Narcissism | .528 | .206 | **< .001** |  |
| Psychopathy | .192 | .380 | **.016** |  |
| Conspiracy Intentions | .376 | **< .001** | **.019** |  |
| *Note.* KS = Kolmogorov-Smirnov | | | | |

| **Table S11** | | | | | | | | | | | | | | | |
| --- | --- | --- | --- | --- | --- | --- | --- | --- | --- | --- | --- | --- | --- | --- | --- |
| *Results of Multi-Level Linear Models: Investigating Separate two-way Interactions of Spread and Believe With Participants’ Conspiracy Beliefs and Political Orientation* | | | | | | | | | | | | | | | |
|  | Competence | | | | | Morality | | | | | Warmth | | | | |
| Effect | *B*  [95% CI] | *SE* | *t* | *df* | *p* | *B*  [95% CI] | *SE* | *t* | *df* | *p* | *B*  [95% CI] | *SE* | *t* | *df* | *p* |
| Fixed effects |  |  |  |  |  |  |  |  |  |  |  |  |  |  |  |
| (Intercept) | 5.40  [5.12, 5.69] | 0.15 | 36.81 | 390.24 | < .001 | 5.24  [4.92, 5.56] | 0.16 | 31.84 | 370.14 | < .001 | 5.04  [4.74, 5.34] | 0.16 | 32.48 | 390.35 | < .001 |
| Spread | -1.88  [-2.25, -1.50] | 0.19 | -9.84 | 415.84 | < .001 | -3.08  [-3.49, -2.66] | 0.21 | -14.56 | 414.56 | < .001 | -2.16  [-2.54, -1.79] | 0.19 | -11.33 | 415.38 | < .001 |
| Believe | -1.94  [-2.28, -1.60] | 0.17 | -11.17 | 410.36 | < .001 | -1.12  [-1.51, -0.72] | 0.20 | -5.51 | 403.43 | < .001 | -0.92  [-1.23, -0.62] | 0.16 | -5.88 | 405.61 | < .001 |
| GCB | -0.16  [-0.22, -0.09] | 0.03 | -4.60 | 388.18 | < .001 | -0.17  [-0.24, -0.09] | 0.04 | -4.32 | 367.84 | < .001 | -0.14  [-0.21, -0.06] | 0.04 | -3.76 | 389.62 | .002 |
| Political orientation | 0.03  [-0.00, 0.07] | 0.02 | 1.96 | 381.86 | .205 | 0.04  [-0.00, 0.07] | 0.02 | 1.83 | 361.06 | .275 | 0.06  [0.02, 0.09] | 0.02 | 3.03 | 383.66 | .016 |
| Political orientation^2 | 8.61  [2.65, 14.57] | 3.04 | 2.83 | 385.23 | .025 | 6.86  [0.17, 13.54] | 3.41 | 2.01 | 364.14 | .227 | 9.21  [2.95, 15.48] | 3.20 | 2.88 | 386.91 | .021 |
| GCB * Spread | **0.15**  **[0.06, 0.24]** | **0.04** | **3.38** | **416.41** | **.005** | **0.26**  **[0.16, 0.36]** | **0.05** | **5.25** | **414.54** | **< .001** | **0.15**  **[0.06, 0.24]** | **0.04** | **3.41** | **415.78** | **.005** |
| GCB * Believe | **0.32**  **[0.24, 0.40]** | **0.04** | **7.95** | **408.74** | **< .001** | **0.25**  **[0.16, 0.34]** | **0.05** | **5.24** | **401.38** | **< .001** | **0.23**  **[0.16, 0.30]** | **0.04** | **6.18** | **403.36** | **< .001** |
| Political orientation  * Spread | 0.01  [-0.03, 0.05] | 0.02 | 0.43 | 412.85 | > .999 | 0.03  [-0.02, 0.08] | 0.03 | 1.10 | 410.66 | .810 | 0.01  [-0.03, 0.06] | 0.02 | 0.62 | 412.30 | > .999 |
| Political orientation  * Believe | **0.07**  **[0.03, 0.11]** | **0.02** | **3.21** | **408.27** | **.008** | 0.06  [0.02, 0.11] | 0.02 | 2.64 | 401.19 | .051 | 0.02  [-0.02, 0.06] | 0.02 | 1.07 | 402.91 | > .999 |
| Political orientation^2 * Spread | -2.86  [-10.67, 4.94] | 3.98 | -0.72 | 414.85 | > .999 | -1.88  [-10.51, 6.75] | 4.40 | -0.43 | 413.25 | > .999 | -0.66  [-8.47, 7.14] | 3.98 | -0.17 | 414.42 | > .999 |
| Political orientation^2 * Believe | -2.25  [-9.36, 4.85] | 3.62 | -0.62 | 407.80 | > .999 | -0.83  [-9.12, 7.45] | 4.23 | -0.20 | 400.70 | > .999 | -3.31  [-9.73, 3.12] | 3.28 | -1.01 | 402.68 | > .999 |
| Random Effects | Variance (*SD*) | | | | | Variance (*SD*) | | | | | Variance (*SD*) | | | | |
| Participants intercept | 0.93 (0.97) | | | | | 1.10 (1.05) | | | | | 1.06 (1.03) | | | | |
| Spread | 1.66 (1.29) | | | | | 1.93 (1.39) | | | | | 1.67 (1.29) | | | | |
| Believe | 1.33 (1.15) | | | | | 1.74 (1.32) | | | | | 1.04 (1.02) | | | | |
| Trial | 0.00 (0.04) | | | | | 0.00 (0.04) | | | | | 0.00 (0.06) | | | | |
| *R^2^* |  |  |  |  |  |  |  |  |  |  |  |  |  |  |  |
| Conditional | .84 | | | | | .80 | | | | | .84 | | | | |
| Marginal | .24 | | | | | .34 | | | | | .29 | | | | |
| *Note.* CI = confidence interval. GCB = Generic Conspiracist Beliefs. Holm-corrected *p*-values are presented. Significant two-way interactions are presented in bold. | | | | | | | | | | | | | | | |

| **Table S12** | | | | | | | | | | | | | | | |
| --- | --- | --- | --- | --- | --- | --- | --- | --- | --- | --- | --- | --- | --- | --- | --- |
| *Results of Multi-Level Linear Models: Investigating Separate two-way Interactions of Spread and Believe With Participants’ Conspiracy Beliefs and Political Orientation* | | | | | | | | | | | | | | | |
|  | Machiavellianism | | | | | Narcissism | | | | | Psychopathy | | | | |
| Effect | *B*  [95% CI] | *SE* | *t* | *df* | *p* | *B*  [95% CI] | *SE* | *t* | *df* | *p* | *B*  [95% CI] | *SE* | *t* | *df* | *p* |
| Fixed effects |  |  |  |  |  |  |  |  |  |  |  |  |  |  |  |
| (Intercept) | 2.19  [1.88, 2.49] | 0.16 | 14.09 | 410.88 | < .001 | 3.39  [3.09, 3.69] | 0.15 | 22.02 | 420.57 | < .001 | 2.11  [1.81, 2.40] | 0.15 | 13.94 | 397.92 | < .001 |
| Spread | 3.11  [2.72, 3.49] | 0.20 | 15.74 | 417.56 | < .001 | 2.31  [1.96, 2.67] | 0.18 | 12.66 | 417.87 | < .001 | 3.14  [2.77, 3.51] | 0.19 | 16.79 | 417.10 | < .001 |
| Believe | 0.59  [0.23, 0.95] | 0.19 | 3.17 | 410.72 | .011 | 0.26  [-0.02, 0.53] | 0.14 | 1.85 | 405.67 | .394 | 0.73  [0.38, 1.08] | 0.18 | 4.10 | 408.64 | < .001 |
| GCB | 0.25  [0.18, 0.32] | 0.04 | 6.98 | 409.92 | < .001 | 0.19  [0.12, 0.26] | 0.04 | 5.18 | 419.14 | < .001 | 0.25  [0.18, 0.31] | 0.04 | 7.03 | 400.14 | < .001 |
| Political orientation | -0.01  [-0.05, 0.02] | 0.02 | -0.63 | 402.56 | > .999 | -0.04  [-0.08, -0.00] | 0.02 | -2.18 | 413.14 | .210 | -0.00  [-0.04, 0.03] | 0.02 | -0.26 | 392.93 | > .999 |
| Political orientation^2 | -5.07  [-11.38, 1.25] | 3.22 | -1.57 | 405.37 | .583 | 3.68  [-2.60, 9.97] | 3.21 | 1.15 | 416.12 | .973 | -3.63  [-9.72, 2.47] | 3.11 | -1.17 | 395.55 | > .999 |
| GCB * Spread | **-0.25**  **[-0.34, -0.16]** | **0.05** | **-5.42** | **417.75** | **< .001** | **-0.24**  **[-0.32, -0.16]** | **0.04** | **-5.58** | **418.14** | **< .001** | **-0.28**  **[-0.37, -0.19]** | **0.04** | **-6.41** | **417.15** | **< .001** |
| GCB * Believe | **-0.16**  **[-0.24, -0.07]** | **0.04** | **-3.63** | **408.46** | **.003** | **-0.10**  **[-0.16, -0.03]** | **0.03** | **-2.93** | **402.09** | **.029** | **-0.15**  **[-0.23, -0.07]** | **0.04** | **-3.61** | **406.53** | **.002** |
| Political orientation  * Spread | -0.01  [-0.06, 0.03] | 0.02 | -0.48 | 413.56 | > .999 | 0.03  [-0.02, 0.07] | 0.02 | 1.17 | 414.24 | .973 | -0.03  [-0.07, 0.02] | 0.02 | -1.26 | 412.98 | > .999 |
| Political orientation  * Believe | **-0.06**  **[-0.11, -0.02]** | **0.02** | **-2.91** | **408.16** | **.023** | -0.03  [-0.06, 0.00] | 0.02 | -1.71 | 401.26 | .439 | **-0.06**  **[-0.10, -0.02]** | **0.02** | **-2.85** | **406.16** | **.028** |
| Political orientation^2 * Spread | 3.59  [-4.47, 11.66] | 4.12 | 0.87 | 416.07 | > .999 | -1.22  [-8.70, 6.25] | 3.81 | -0.32 | 416.65 | .973 | 3.66  [-3.98, 11.30] | 3.90 | 0.94 | 415.53 | > .999 |
| Political orientation^2 * Believe | 3.18  [-4.41, 10.76] | 3.87 | 0.82 | 407.54 | > .999 | 2.69  [-3.04, 8.42] | 2.92 | 0.92 | 401.47 | .937 | 1.38  [-5.92, 8.69] | 3.73 | 0.37 | 405.45 | > .999 |
| Random Effects | Variance (*SD*) | | | | | Variance (*SD*) | | | | | Variance (*SD*) | | | | |
| Participants intercept | 0.95 (0.98) | | | | | 1.03 (1.02) | | | | | 0.89 (0.94) | | | | |
| Spread | 1.65 (1.28) | | | | | 1.48 (1.22) | | | | | 1.46 (1.21) | | | | |
| Believe | 1.40 (1.18) | | | | | 0.73 (0.86) | | | | | 1.30 (1.14) | | | | |
| Trial | 0.00 (0.03) | | | | | 0.00 (0.00) | | | | | 0.00 (0.07) | | | | |
| *R^2^* |  |  |  |  |  |  |  |  |  |  |  |  |  |  |  |
| Conditional | .79 | | | | | .78 | | | | | .78 | | | | |
| Marginal | .39 | | | | | .31 | | | | | .37 | | | | |
| *Note.* CI = confidence interval. GCB = Generic Conspiracist Beliefs. Holm-corrected *p*-values are presented. Significant two-way interactions are presented in bold. | | | | | | | | | | | | | | | |

| **Table S13** | | | | | | | | | | | | | | | |
| --- | --- | --- | --- | --- | --- | --- | --- | --- | --- | --- | --- | --- | --- | --- | --- |
| *Results of Multi-Level Linear Models: Investigating Separate two-way Interactions of Spread and Believe With Participants’ Conspiracy Beliefs and Political Orientation* | | | | | | | | | | | | | | | |
|  | Contact Willingness | | | | | Conspiracy Intentions | | | | |  | | | | |
| Effect | *B*  [95% CI] | *SE* | *t* | *df* | *p* | *B*  [95% CI] | *SE* | *t* | *df* | *p* |  |  |  |  |  |
| Fixed effects |  |  |  |  |  |  |  |  |  |  |  |  |  |  |  |
| (Intercept) | 5.53  [5.25, 5.81] | 0.14 | 38.59 | 380.01 | < .001 | 1.49  [1.16, 1.82] | 0.17 | 8.84 | 399.15 | < .001 |  |  |  |  |  |
| Spread | -3.11  [-3.50, -2.71] | 0.20 | -15.47 | 412.60 | < .001 | 2.31  [1.92, 2.71] | 0.20 | 11.45 | 414.56 | < .001 |  |  |  |  |  |
| Believe | -1.96  [-2.33, -1.60] | 0.19 | -10.54 | 405.67 | < .001 | 0.86  [0.44, 1.27] | 0.21 | 4.03 | 409.23 | < .001 |  |  |  |  |  |
| GCB | -0.18  [-0.25, -0.12] | 0.03 | -5.45 | 381.13 | < .001 | 0.37  [0.29, 0.44] | 0.04 | 9.42 | 401.21 | < .001 |  |  |  |  |  |
| Political orientation | 0.03  [-0.01, 0.08] | 0.02 | 1.54 | 374.39 | .497 | -0.02  [-0.06, 0.02] | 0.02 | -0.94 | 393.98 | > .999 |  |  |  |  |  |
| Political orientation^2 | 7.23  [1.45, 13.01] | 2.95 | 2.45 | 377.47 | .073 | -4.40  [-11.18, 2.37] | 3.46 | -1.27 | 397.24 | > .999 |  |  |  |  |  |
| GCB * Spread | **0.25**  **[0.16, 0.35]** | **0.05** | **5.39** | **412.79** | **< .001** | **-0.25**  **[-0.34, -0.16]** | **0.05** | **-5.30** | **414.50** | **< .001** |  |  |  |  |  |
| GCB * Believe | **0.35**  **[0.27, 0.44]** | **0.04** | **8.08** | **404.13** | **< .001** | **-0.22**  **[-0.32, -0.12]** | **0.05** | **-4.39** | **407.67** | **< .001** |  |  |  |  |  |
| Political orientation  * Spread | 0.03  [-0.01, 0.08] | 0.02 | 1.44 | 409.33 | .497 | 0.02  [-0.03, 0.06] | 0.02 | 0.63 | 410.24 | > .999 |  |  |  |  |  |
| Political orientation  * Believe | **0.09**  **[0.05, 0.14]** | **0.02** | **4.13** | **403.83** | **< .001** | -0.05  [-0.10, -0.00] | 0.03 | -2.14 | 407.32 | .196 |  |  |  |  |  |
| Political orientation^2 * Spread | -1.50  [-9.71, 6.71] | 4.19 | -0.36 | 411.53 | .720 | 2.96  [-5.30, 11.21] | 4.21 | 0.70 | 412.96 | > .999 |  |  |  |  |  |
| Political orientation^2 * Believe | -5.42  [-13.04, 2.20] | 3.89 | -1.39 | 403.09 | .497 | 3.82  [-4.86, 12.49] | 4.43 | 0.86 | 406.61 | > .999 |  |  |  |  |  |
| Random Effects | Variance (*SD*) | | | | | Variance (*SD*) | | | | | Variance (*SD*) | | | | |
| Participants intercept | 0.83 (0.91) | | | | | 1.14 (1.07) | | | | |  | | | | |
| Spread | 1.83 (1.35) | | | | | 1.72 (1.31) | | | | |  | | | | |
| Believe | 1.53 (1.24) | | | | | 1.96 (1.40) | | | | |  | | | | |
| Trial | 0.00 (0.06) | | | | | 0.01 (0.07) | | | | |  | | | | |
| *R^2^* |  |  |  |  |  |  |  |  |  |  |  |  |  |  |  |
| Conditional | .85 | | | | | .78 | | | | |  | | | | |
| Marginal | .40 | | | | | .22 | | | | |  | | | | |
| *Note.* CI = confidence interval. GCB = Generic Conspiracist Beliefs. Holm-corrected *p*-values are presented. Significant two-way interactions are presented in bold. | | | | | | | | | | | | | | | |

| **Table S14** | | | | |
| --- | --- | --- | --- | --- |
| *Residual Diagnostics for Multi-Level Linear Models Presented in Table 4 in the Main Document* | | | | |
| Models | *p*  Dispersion Test | *p*  Outlier Test | *p*  KS Test |  |
| Competence | .536 | **.001** | **.012** |  |
| Morality | .296 | .172 | **< .001** |  |
| Warmth | .600 | .098 | .098 |  |
| Contact Willingness | .232 | .846 | **.003** |  |
| Machiavellianism | .640 | .284 | **.002** |  |
| Narcissism | .592 | .922 | **< .001** |  |
| Psychopathy | .280 | .118 | .074 |  |
| Conspiracy Intentions | .424 | **< .001** | **.003** |  |
| *Note.* KS = Kolmogorov-Smirnov | | | | |

| **Table S15** | | | | | | | | | | | | | | | |
| --- | --- | --- | --- | --- | --- | --- | --- | --- | --- | --- | --- | --- | --- | --- | --- |
| *Results of Multi-Level Linear Models: Investigating Three-Way Interactions of Spread and Believe With Participants’ Conspiracy Beliefs and Political Orientation* | | | | | | | | | | | | | | | |
|  | Competence | | | | | Morality | | | | | Warmth | | | | |
| Effect | *B*  [95% CI] | *SE* | *t* | *df* | *p* | *B*  [95% CI] | *SE* | *t* | *df* | *p* | *B*  [95% CI] | *SE* | *t* | *df* | *p* |
| Fixed effects |  |  |  |  |  |  |  |  |  |  |  |  |  |  |  |
| (Intercept) | 5.72  [5.43, 6.01] | 0.15 | 38.83 | 445.56 | < .001 | 5.91  [5.60, 6.23] | 0.16 | 36.90 | 442.32 | < .001 | 5.38  [5.08, 5.69] | 0.16 | 34.44 | 440.78 | < .001 |
| Spread | -2.55  [-2.95, -2.15] | 0.21 | -12.40 | 570.23 | < .001 | -4.46  [-4.90, -4.03] | 0.22 | -20.32 | 590.71 | < .001 | -2.87  [-3.28, -2.47] | 0.20 | -14.04 | 564.91 | < .001 |
| Believe | -2.55  [-2.92, -2.18] | 0.19 | -13.55 | 566.36 | < .001 | -2.39  [-2.80, -1.98] | 0.21 | -11.42 | 568.39 | < .001 | -1.57  [-1.90, -1.24] | 0.17 | -9.24 | 590.65 | < .001 |
| GCB | -0.17  [-0.24, -0.10] | 0.03 | -4.93 | 443.44 | < .001 | -0.20  [-0.27, -0.12] | 0.04 | -5.24 | 440.59 | < .001 | -0.15  [-0.22, -0.08] | 0.04 | -4.10 | 439.99 | < .001 |
| Political orientation | 0.03  [-0.01, 0.06] | 0.02 | 1.45 | 433.39 | .595 | 0.02  [-0.01, 0.06] | 0.02 | 1.25 | 430.36 | .583 | 0.05  [0.01, 0.08] | 0.02 | 2.56 | 430.12 | .075 |
| Political orientation^2 | 9.50  [3.51, 15.59] | 3.05 | 3.11 | 439.77 | .016 | 8.10  [1.58, 14.61] | 3.32 | 2.44 | 436.54 | .084 | 10.95  [4.64, 17.26] | 3.22 | 3.40 | 436.85 | .007 |
| GCB * Spread | 0.17  [0.08, 0.27] | 0.05 | 3.62 | 564.43 | .003 | 0.31  [0.21, 0.42] | 0.05 | 6.15 | 583.98 | < .001 | 0.18  [0.08, 0.27] | 0.05 | 3.72 | 559.13 | .002 |
| GCB * Believe | 0.35  [0.26, 0.43] | 0.04 | 7.85 | 573.04 | < .001 | 0.30  [0.21, 0.40] | 0.05 | 6.20 | 575.05 | < .001 | 0.25  [0.18, 0.33] | 0.04 | 6.36 | 597.50 | < .001 |
| Political orientation  * Spread | 0.03  [-0.02, 0.08] | 0.02 | 1.26 | 558.33 | .620 | 0.06  [0.01, 0.11] | 0.03 | 2.24 | 577.47 | .103 | 0.03  [-0.01, 0.08] | 0.02 | 1.40 | 552.61 | .323 |
| Political orientation  * Believe | 0.08  [0.04, 0.13] | 0.02 | 3.70 | 550.84 | .002 | 0.09  [0.04, 0.13] | 0.02 | 3.44 | 552.90 | .006 | 0.03  [-0.00, 0.07] | 0.02 | 1.72 | 572.03 | .314 |
| Political orientation^2 * Spread | -5.43  [-13.86, 2.99] | 4.30 | -1.26 | 575.32 | .620 | -5.97  [-14.98, 3.04] | 4.60 | -1.30 | 596.58 | .583 | -5.03  [-13.42, 3.36] | 4.28 | -1.17 | 569.60 | .323 |
| Political orientation^2 * Believe | -4.00  [-11.66, 3.66] | 3.91 | -1.02 | 553.65 | .620 | -3.41  [-11.93, 5.11] | 4.35 | -0.79 | 555.67 | .583 | -6.57  [-13.48, 0.34] | 3.52 | -1.86 | 576.06 | .314 |
| Spread * Believe | 1.31  [1.00, 1.62] | 0.16 | 8.35 | 2779.11 | < .001 | 2.71  [2.37, 3.06] | 0.18 | 15.32 | 2834.93 | < .001 | 1.38  [1.08, 1.68] | 0.15 | 9.06 | 2791.27 | < .001 |
| GCB * Spread * Believe | -0.06  [-0.13, 0.01] | 0.04 | -1.57 | 2722.40 | .585 | **-0.13**  **[-0.21, -0.05]** | **0.04** | **-3.15** | **2771.18** | **.013** | -0.06  [-0.13, 0.01] | 0.04 | -1.77 | 2732.54 | .314 |
| Political orientation * Spread * Believe | -0.04  [-0.07, -0.00] | 0.02 | -2.03 | 2637.85 | .301 | -0.05  [-0.09, -0.01] | 0.02 | -2.51 | 2682.50 | .084 | -0.03  [-0.07, 0.00] | 0.02 | -1.95 | 2644.27 | .308 |
| Political orientation^2 * Spread * Believe | 5.41  [-0.90, 11.71] | 3.22 | 1.68 | 2710.93 | .558 | 8.99  [1.87, 16.12] | 3.63 | 2.47 | 2758.68 | .084 | **8.69**  **[2.56, 14.82]** | **3.13** | **2.78** | **2719.91** | **.044** |
| Random Effects | Variance (*SD*) | | | | | Variance (*SD*) | | | | | Variance (*SD*) | | | | |
| Participants intercept | 0.88 (0.94) | | | | | 1.01 (1.01) | | | | | 1.02 (1.01) | | | | |
| Spread | 1.65 (1.28) | | | | | 1.81 (1.34) | | | | | 1.66 (1.29) | | | | |
| Believe | 1.34 (1.16) | | | | | 1.63 (1.28) | | | | | 1.01 (1.01) | | | | |
| Trial | 0.00 (0.04) | | | | | 0.00 (0.03) | | | | | 0.00 (0.06) | | | | |
| *R^2^* |  |  |  |  |  |  |  |  |  |  |  |  |  |  |  |
| Conditional | .85 | | | | | .86 | | | | | .86 | | | | |
| Marginal | .26 | | | | | .41 | | | | | .31 | | | | |
| *Note.* CI = confidence interval. GCB = Generic Conspiracist Beliefs. Holm-corrected *p*-values are presented. Significant three-way interactions are presented in bold. | | | | | | | | | | | | | | | |

| **Table S16** | | | | | | | | | | | | | | | |
| --- | --- | --- | --- | --- | --- | --- | --- | --- | --- | --- | --- | --- | --- | --- | --- |
| *Results of Multi-Level Linear Models: Investigating Three-Way Interactions of Spread and Believe With Participants’ Conspiracy Beliefs and Political Orientation* | | | | | | | | | | | | | | | |
|  | Machiavellianism | | | | | Narcissism | | | | | Psychopathy | | | | |
| Effect | *B*  [95% CI] | *SE* | *t* | *df* | *p* | *B*  [95% CI] | *SE* | *t* | *df* | *p* | *B*  [95% CI] | *SE* | *t* | *df* | *p* |
| Fixed effects |  |  |  |  |  |  |  |  |  |  |  |  |  |  |  |
| (Intercept) | 1.86  [1.54, 2.17] | 0.16 | 11.56 | 475.33 | < .001 | 3.31  [3.00, 3.62] | 0.16 | 20.75 | 473.99 | < .001 | 1.71  [1.41, 2.01] | 0.15 | 11.16 | 473.70 | < .001 |
| Spread | 3.78  [3.36, 4.21] | 0.22 | 17.50 | 626.55 | < .001 | 2.48  [2.09, 2.88] | 0.20 | 12.38 | 602.26 | < .001 | 3.94  [3.54, 4.34] | 0.20 | 19.30 | 631.27 | < .001 |
| Believe | 1.23  [0.83, 1.62] | 0.20 | 6.09 | 631.66 | < .001 | 0.41  [0.09, 0.73] | 0.16 | 2.53 | 681.05 | .138 | 1.50  [1.13, 1.88] | 0.19 | 7.83 | 629.85 | < .001 |
| GCB | 0.26  [0.18, 0.33] | 0.04 | 6.80 | 474.76 | < .001 | 0.16  [0.09, 0.24] | 0.04 | 4.37 | 471.94 | < .001 | 0.26  [0.19, 0.33] | 0.04 | 7.43 | 474.47 | < .001 |
| Political orientation | -0.01  [-0.05, 0.03] | 0.02 | -0.55 | 464.31 | > .999 | -0.03  [-0.07, 0.01] | 0.02 | -1.61 | 462.21 | .758 | -0.00  [-0.04, 0.03] | 0.02 | -0.27 | 463.91 | > .999 |
| Political orientation^2 | -4.50  [-11.04, 2.04] | 3.34 | -1.35 | 468.94 | > .999 | 5.03  [-1.48, 11.53] | 3.32 | 1.52 | 467.72 | .782 | -4.44  [-10.64, 1.76] | 3.16 | -1.40 | 468.38 | .862 |
| GCB * Spread | -0.25  [-0.35, -0.15] | 0.05 | -5.00 | 619.57 | < .001 | -0.19  [-0.28, -0.10] | 0.05 | -4.07 | 595.95 | < .001 | -0.32  [-0.41, -0.22] | 0.05 | -6.62 | 624.11 | < .001 |
| GCB * Believe | -0.16  [-0.25, -0.07] | 0.05 | -3.37 | 640.66 | .008 | -0.05  [-0.12, 0.03] | 0.04 | -1.23 | 690.74 | .877 | -0.19  [-0.28, -0.10] | 0.05 | -4.15 | 638.83 | < .001 |
| Political orientation  * Spread | -0.02  [-0.07, 0.03] | 0.03 | -0.67 | 613.25 | > .999 | 0.00  [-0.04, 0.05] | 0.02 | 0.20 | 590.09 | > .999 | -0.03  [-0.08, 0.02] | 0.02 | -1.26 | 617.64 | .862 |
| Political orientation  * Believe | -0.07  [-0.11, -0.02] | 0.02 | -2.79 | 613.18 | .049 | -0.05  [-0.08, -0.01] | 0.02 | -2.48 | 658.23 | .145 | -0.06  [-0.10, -0.02] | 0.02 | -2.62 | 611.22 | .080 |
| Political orientation^2 * Spread | 3.22  [-5.64, 12.08] | 4.52 | 0.71 | 633.80 | > .999 | -3.85  [-12.07, 4.38] | 4.20 | -0.92 | 608.55 | > .999 | 6.26  [-2.12, 14.64] | 4.28 | 1.46 | 638.55 | .862 |
| Political orientation^2 * Believe | 2.17  [-6.02, 10.36] | 4.18 | 0.52 | 614.50 | > .999 | 0.13  [-6.43, 6.70] | 3.35 | 0.04 | 659.65 | > .999 | 3.08  [-4.71, 10.87] | 3.98 | 0.77 | 612.51 | > .999 |
| Spread * Believe | -1.34  [-1.71, -0.96] | 0.19 | -7.03 | 2956.21 | < .001 | -0.32  [-0.65, 0.00] | 0.17 | -1.95 | 2932.11 | .414 | -1.60  [-1.96, -1.25] | 0.18 | -8.84 | 2946.46 | < .001 |
| GCB * Spread * Believe | 0.01  [-0.07, 0.10] | 0.04 | 0.31 | 2895.78 | > .999 | -0.09  [-0.17, -0.02] | 0.04 | -2.42 | 2876.89 | .156 | 0.08  [-0.00, 0.16] | 0.04 | 1.94 | 2886.14 | .421 |
| Political orientation * Spread * Believe | 0.01  [-0.04, 0.05] | 0.02 | 0.36 | 2818.10 | > .999 | 0.04  [0.00, 0.08] | 0.02 | 2.04 | 2807.22 | .377 | 0.00  [-0.04, 0.04] | 0.02 | 0.06 | 2808.20 | > .999 |
| Political orientation^2 * Spread * Believe | 0.02  [-7.63, 7.68] | 3.91 | 0.01 | 2879.79 | > .999 | 4.74  [-1.99, 11.48] | 3.44 | 1.38 | 2866.98 | .839 | -5.76  [-13.06, 1.54] | 3.72 | -1.55 | 2870.11 | .853 |
| Random Effects | Variance (*SD*) | | | | | Variance (*SD*) | | | | | Variance (*SD*) | | | | |
| Participants intercept | 0.96 (0.98) | | | | | 1.03 (1.02) | | | | | 0.86 (0.93) | | | | |
| Spread | 1.62 (1.27) | | | | | 1.47 (1.21) | | | | | 1.43 (1.20) | | | | |
| Believe | 1.31 (1.15) | | | | | 0.72 (0.85) | | | | | 1.19 (1.09) | | | | |
| Trial | 0.00 (0.02) | | | | | 0.00 (0.00) | | | | | 0.00 (0.06) | | | | |
| *R^2^* |  |  |  |  |  |  |  |  |  |  |  |  |  |  |  |
| Conditional | .81 | | | | | .79 | | | | | .81 | | | | |
| Marginal | .42 | | | | | .32 | | | | | .40 | | | | |
| *Note.* CI = confidence interval. GCB = Generic Conspiracist Beliefs. Holm-corrected *p*-values are presented. Significant three-way interactions are presented in bold. | | | | | | | | | | | | | | | |

| **Table S17** | | | | | | | | | | | | | | | |
| --- | --- | --- | --- | --- | --- | --- | --- | --- | --- | --- | --- | --- | --- | --- | --- |
| *Results of Multi-Level Linear Models: Investigating Three-Way Interactions of Spread and Believe With Participants’ Conspiracy Beliefs and Political Orientation* | | | | | | | | | | | | | | | |
|  | Contact Willingness | | | | | Conspiracy Intentions | | | | |  | | | | |
| Effect | *b*  [95% CI] | *SE* | *t* | *df* | *p* | *b*  [95% CI] | *SE* | *t* | *df* | *p* |  |  |  |  |  |
| Fixed effects |  |  |  |  |  |  |  |  |  |  |  |  |  |  |  |
| (Intercept) | 6.04  [5.75, 6.32] | 0.15 | 41.38 | 437.84 | < .001 | 1.22  [0.88, 1.56] | 0.17 | 7.08 | 463.87 | < .001 |  |  |  |  |  |
| Spread | -4.15  [-4.57, -3.74] | 0.21 | -19.61 | 558.33 | < .001 | 2.88  [2.45, 3.31] | 0.22 | 13.08 | 628.93 | < .001 |  |  |  |  |  |
| Believe | -2.92  [-3.31, -2.54] | 0.20 | -14.92 | 551.32 | < .001 | 1.36  [0.92, 1.81] | 0.23 | 6.04 | 578.75 | < .001 |  |  |  |  |  |
| GCB | -0.20  [-0.26, -0.13] | 0.03 | -5.87 | 438.94 | < .001 | 0.35  [0.27, 0.42] | 0.04 | 8.66 | 463.85 | < .001 |  |  |  |  |  |
| Political orientation | 0.01  [-0.02, 0.04] | 0.02 | 0.60 | 429.00 | .549 | -0.02  [-0.06, 0.02] | 0.02 | -0.84 | 452.94 | > .999 |  |  |  |  |  |
| Political orientation^2 | 9.27  [3.39, 15.15] | 3.00 | 3.09 | 435.18 | .013 | -6.13  [-13.08, 0.83] | 3.55 | -1.73 | 458.90 | .595 |  |  |  |  |  |
| GCB * Spread | 0.28  [0.19, 0.38] | 0.05 | 5.75 | 552.47 | < .001 | -0.21  [-0.31, -0.11] | 0.05 | -4.03 | 621.36 | < .001 |  |  |  |  |  |
| GCB * Believe | 0.38  [0.29, 0.47] | 0.05 | 8.32 | 557.70 | < .001 | -0.18  [-0.28, -0.07] | 0.05 | -3.34 | 585.93 | .009 |  |  |  |  |  |
| Political orientation  * Spread | 0.07  [0.02, 0.12] | 0.03 | 2.81 | 546.59 | .026 | 0.01  [-0.04, 0.06] | 0.03 | 0.28 | 613.75 | > .999 |  |  |  |  |  |
| Political orientation  * Believe | 0.12  [0.08, 0.17] | 0.02 | 5.20 | 537.22 | < .001 | -0.06  [-0.11, -0.00] | 0.03 | -2.14 | 563.22 | .284 |  |  |  |  |  |
| Political orientation^2 * Spread | -6.64  [-15.33, 2.04] | 4.43 | -1.50 | 562.77 | .269 | 7.26  [-1.78, 16.29] | 4.61 | 1.57 | 636.32 | .695 |  |  |  |  |  |
| Political orientation^2 * Believe | -9.34  [-17.31, -1.37] | 4.07 | -2.30 | 539.59 | .088 | 6.84  [-2.34, 16.02] | 4.69 | 1.46 | 565.50 | .695 |  |  |  |  |  |
| Spread * Believe | 2.05  [1.74, 2.36] | 0.16 | 12.95 | 2775.54 | < .001 | -1.11  [-1.49, -0.73] | 0.19 | -5.75 | 2873.46 | < .001 |  |  |  |  |  |
| GCB * Spread * Believe | -0.07  [-0.15, -0.00] | 0.04 | -2.03 | 2714.94 | .127 | -0.07  [-0.16, 0.02] | 0.04 | -1.56 | 2811.34 | .695 |  |  |  |  |  |
| Political orientation * Spread * Believe | **-0.06**  **[-0.10, -0.03]** | **0.02** | **-3.55** | **2628.67** | **.003** | 0.01  [-0.03, 0.05] | 0.02 | 0.43 | 2726.62 | > .999 |  |  |  |  |  |
| Political orientation^2 * Spread * Believe | **10.68**  **[4.34, 17.03]** | **3.24** | **3.30** | **2704.19** | **.007** | -8.55  [-16.34, -0.76] | 3.97 | -2.15 | 2799.75 | .284 |  |  |  |  |  |
| Random Effects | Variance (*SD*) | | | | | Variance (*SD*) | | | | |  | | | | |
| Participants intercept | 0.83 (0.91) | | | | | 1.13 (1.06) | | | | |  | | | | |
| Spread | 1.79 (1.34) | | | | | 1.66 (1.29) | | | | |  | | | | |
| Believe | 1.49 (1.22) | | | | | 1.86 (1.36) | | | | |  | | | | |
| Trial | 0.00 (0.06) | | | | | 0.00 (0.06) | | | | |  | | | | |
| *R^2^* |  |  |  |  |  |  |  |  |  |  |  |  |  |  |  |
| Conditional | .88 | | | | | .81 | | | | |  | | | | |
| Marginal | .44 | | | | | .26 | | | | |  | | | | |
| *Note.* CI = confidence interval. GCB = Generic Conspiracist Beliefs. Holm-corrected *p*-values are presented. Significant three-way interactions are presented in bold. | | | | | | | | | | | | | | | |

| **Table S18** | | | | | | | | | | | | | | | |
| --- | --- | --- | --- | --- | --- | --- | --- | --- | --- | --- | --- | --- | --- | --- | --- |
| *Results of Multi-Level Linear Models: Investigating Three-Way Interactions of Spread and Believe With Gender of the Fictitious Characters* | | | | | | | | | | | | | | | |
|  | Competence | | | | | Morality | | | | | Warmth | | | | |
| Effect | *B*  [95% CI] | *SE* | *t* | *df* | *p* | *B*  [95% CI] | *SE* | *t* | *df* | *p* | *B*  [95% CI] | *SE* | *t* | *df* | *p* |
| Fixed effects |  |  |  |  |  |  |  |  |  |  |  |  |  |  |  |
| (Intercept) | 5.24  [5.12, 5.36] | 0.06 | 84.58 | 194.11 | < .001 | 5.30  [5.17, 5.42] | 0.07 | 80.92 | 317.59 | < .001 | 5.08  [4.96, 5.21] | 0.06 | 79.51 | 203.43 | < .001 |
| Spread | -1.76  [-1.92, -1.60] | 0.08 | -21.26 | 813.13 | < .001 | -2.96  [-3.14, -2.78] | 0.09 | -32.38 | 849.85 | < .001 | -2.09  [-2.25, -1.93] | 0.08 | -25.40 | 792.73 | < .001 |
| Believe | -0.80  [-0.96, -0.64] | 0.08 | -9.75 | 783.85 | < .001 | -0.78  [-0.95, -0.60] | 0.09 | -8.76 | 837.37 | < .001 | -0.45  [-0.60, -0.31] | 0.07 | -6.29 | 894.46 | < .001 |
| Gender ^a^ | 0.04  [-0.07, 0.15] | 0.06 | 0.69 | 46.34 | > .999 | 0.10  [-0.01, 0.21] | 0.06 | 1.77 | 99.91 | .319 | 0.08  [-0.03, 0.19] | 0.06 | 1.41 | 42.24 | .661 |
| Spread * Believe | 0.89  [0.74, 1.03] | 0.08 | 11.76 | 2449.93 | < .001 | 1.91  [1.74, 2.08] | 0.09 | 22.31 | 2460.29 | < .001 | 0.99  [0.85, 1.14] | 0.07 | 13.59 | 2468.30 | < .001 |
| Gender * Spread | 0.02  [-0.12, 0.16] | 0.07 | 0.26 | 2283.47 | > .999 | -0.09  [-0.26, 0.07] | 0.08 | -1.14 | 2300.91 | .765 | 0.08  [-0.06, 0.22] | 0.07 | 1.18 | 2268.72 | .715 |
| Gender * Believe | -0.08  [-0.22, 0.06] | 0.07 | -1.12 | 2292.20 | > .999 | -0.08  [-0.23, 0.08] | 0.08 | -0.94 | 2293.87 | .765 | -0.02  [-0.15, 0.12] | 0.07 | -0.25 | 2286.68 | > .999 |
| Gender * Spread * Believe | 0.04  [-0.16, 0.25] | 0.10 | 0.43 | 2239.01 | > .999 | 0.12  [-0.11, 0.35] | 0.12 | 1.04 | 2246.25 | .765 | -0.05  [-0.25, 0.15] | 0.10 | -0.48 | 2239.01 | > .999 |
| Random Effects | Variance (*SD*) | | | | | Variance (*SD*) | | | | | Variance (*SD*) | | | | |
| Participants intercept | 0.94 (0.97) | | | | | 1.08 (1.04) | | | | | 1.05 (1.02) | | | | |
| Spread | 1.71 (1.31) | | | | | 2.00 (1.42) | | | | | 1.73 (1.31) | | | | |
| Believe | 1.71 (1.31) | | | | | 1.87 (1.37) | | | | | 1.15 (1.07) | | | | |
| Gender | 0.01 (1.11) | | | | | 0.00 (0.05) | | | | | 0.01 (0.10) | | | | |
| Trial | 0.00 (0.04) | | | | | 0.00 (0.01) | | | | | 0.00 (0.04) | | | | |
| *R^2^* |  |  |  |  |  |  |  |  |  |  |  |  |  |  |  |
| Conditional | .86 | | | | | .86 | | | | | .86 | | | | |
| Marginal | .19 | | | | | .36 | | | | | .26 | | | | |
| *Note.* CI = confidence interval. GCB = Generic Conspiracist Beliefs. Holm-corrected *p*-values are presented.  ^a^ 0 = male, 1 = female. | | | | | | | | | | | | | | | |

| **Table S19** | | | | | | | | | | | | | | | |
| --- | --- | --- | --- | --- | --- | --- | --- | --- | --- | --- | --- | --- | --- | --- | --- |
| *Results of Multi-Level Linear Models: Investigating Three-Way Interactions of Spread and Believe With Gender of the Fictitious Characters* | | | | | | | | | | | | | | | |
|  | Machiavellianism | | | | | Narcissism | | | | | Psychopathy | | | | |
| Effect | *B*  [95% CI] | *SE* | *t* | *df* | *p* | *B*  [95% CI] | *SE* | *t* | *df* | *p* | *B*  [95% CI] | *SE* | *t* | *df* | *p* |
| Fixed effects |  |  |  |  |  |  |  |  |  |  |  |  |  |  |  |
| (Intercept) | 2.73  [2.60, 2.86] | 0.07 | 40.59 | 611.25 | < .001 | 3.77  [3.64, 3.89] | 0.06 | 58.60 | 570.66 | < .001 | 2.62  [2.49, 2.75] | 0.07 | 39.52 | 172.45 | < .001 |
| Spread | 2.82  [2.65, 3.00] | 0.09 | 30.95 | 953.39 | < .001 | 1.74  [1.58, 1.91] | 0.08 | 20.89 | 895.12 | < .001 | 2.71  [2.54, 2.88] | 0.09 | 31.00 | 945.25 | < .001 |
| Believe | 0.29  [0.12, 0.46] | 0.09 | 3.40 | 1023.84 | .004 | -0.09  [-0.23, 0.05] | 0.07 | -1.31 | 1195.41 | .348 | 0.54  [0.38, 0.70] | 0.08 | 6.55 | 1020.76 | < .001 |
| Gender ^a^ | -0.04  [-0.16, 0.08] | 0.06 | -0.62 | 2394.50 | > .999 | -0.09  [-0.20, 0.02] | 0.06 | -1.57 | 1776.22 | .348 | 0.03  [-0.10, 0.15] | 0.07 | 0.40 | 52.20 | > .999 |
| Spread * Believe | -1.31  [-1.49, -1.13] | 0.09 | -14.24 | 2552.11 | < .001 | -0.30  [-0.46, -0.15] | 0.08 | -3.75 | 2606.42 | .001 | -1.34  [-1.51, -1.16] | 0.09 | -15.21 | 2540.08 | < .001 |
| Gender * Spread | -0.08  [-0.26, 0.10] | 0.09 | -0.90 | 2489.64 | > .999 | 0.16  [0.01, 0.32] | 0.08 | 2.03 | 2504.66 | .170 | -0.14  [-0.31, 0.03] | 0.09 | -1.66 | 2474.83 | .385 |
| Gender * Believe | -0.04  [-0.21, 0.14] | 0.09 | -0.41 | 2386.81 | > .999 | 0.12  [-0.04, 0.27] | 0.08 | 1.48 | 2396.70 | .348 | -0.09  [-0.25, 0.07] | 0.08 | -1.08 | 2377.33 | .845 |
| Gender * Spread * Believe | 0.12  [-0.13, 0.38] | 0.13 | 0.96 | 2486.36 | > .999 | -0.27  [-0.49, -0.05] | 0.11 | -2.38 | 2519.88 | .087 | 0.05  [-0.19, 0.29] | 0.12 | 0.42 | 2473.31 | > .999 |
| Random Effects | Variance (*SD*) | | | | | Variance (*SD*) | | | | | Variance (*SD*) | | | | |
| Participants intercept | 1.07 (1.04) | | | | | 1.09 (1.04) | | | | | 0.94 (0.97) | | | | |
| Spread | 1.79 (1.34) | | | | | 1.59 (1.26) | | | | | 1.65 (1.29) | | | | |
| Believe | 1.43 (1.20) | | | | | 0.76 (0.87) | | | | | 1.29 (1.14) | | | | |
| Gender | 0.00 (0.06) | | | | | 0.06 (0.24) | | | | | 0.00 (0.06) | | | | |
| Trial | 0.00 (0.00) | | | | | 0.00 (0.00) | | | | | 0.00 (0.04) | | | | |
| *R^2^* |  |  |  |  |  |  |  |  |  |  |  |  |  |  |  |
| Conditional | .81 | | | | | .80 | | | | | .81 | | | | |
| Marginal | .39 | | | | | .29 | | | | | .37 | | | | |
| *Note.* CI = confidence interval. GCB = Generic Conspiracist Beliefs. Holm-corrected *p*-values are presented.  ^a^ 0 = male, 1 = female. | | | | | | | | | | | | | | | |

| **Table S20** | | | | | | | | | | | | | | | |
| --- | --- | --- | --- | --- | --- | --- | --- | --- | --- | --- | --- | --- | --- | --- | --- |
| *Results of Multi-Level Linear Models: Investigating Three-Way Interactions of Spread and Believe With Gender of the Fictitious Characters* | | | | | | | | | | | | | | | |
|  | Contact Willingness | | | | | Conspiracy Intentions | | | | |  | | | | |
| Effect | *B*  [95% CI] | *SE* | *t* | *df* | *p* | *B*  [95% CI] | *SE* | *t* | *df* | *p* |  |  |  |  |  |
| Fixed effects |  |  |  |  |  |  |  |  |  |  |  |  |  |  |  |
| (Intercept) | 5.36  [5.24, 5.47] | 0.06 | 91.69 | 324.41 | < .001 | 2.38  [2.24, 2.53] | 0.08 | 31.31 | 150.80 | < .001 |  |  |  |  |  |
| Spread | -2.78  [-2.95, -2.61] | 0.09 | -32.00 | 762.98 | < .001 | 2.22  [2.04, 2.40] | 0.09 | 24.12 | 962.90 | < .001 |  |  |  |  |  |
| Believe | -0.91  [-1.08, -0.74] | 0.09 | -10.60 | 742.41 | < .001 | 0.42  [0.24, 0.61] | 0.09 | 4.51 | 868.68 | < .001 |  |  |  |  |  |
| Gender ^a^ | 0.06  [-0.04, 0.16] | 0.05 | 1.19 | 111.79 | .496 | -0.04  [-0.18, 0.10] | 0.07 | -0.53 | 39.05 | > .999 |  |  |  |  |  |
| Spread * Believe | 1.51  [1.36, 1.66] | 0.08 | 19.80 | 2427.39 | < .001 | -1.37  [-1.55, -1.18] | 0.09 | -14.69 | 2501.85 | < .001 |  |  |  |  |  |
| Gender * Spread | 0.10  [-0.04, 0.25] | 0.07 | 1.38 | 2302.39 | .496 | -0.07  [-0.25, 0.11] | 0.09 | -0.80 | 2377.20 | > .999 |  |  |  |  |  |
| Gender * Believe | 0.10  [-0.04, 0.24] | 0.07 | 1.39 | 2282.92 | .496 | -0.05  [-0.22, 0.12] | 0.09 | -0.58 | 2339.81 | > .999 |  |  |  |  |  |
| Gender * Spread * Believe | -0.16  [-0.37, 0.04] | 0.11 | -1.56 | 2230.02 | .479 | 0.11  [-0.15, 0.36] | 0.13 | 0.82 | 2382.31 | > .999 |  |  |  |  |  |
| Random Effects | Variance (*SD*) | | | | | Variance (*SD*) | | | | |  | | | | |
| Participants intercept | 0.87 (0.93) | | | | | 1.31 (1.15) | | | | |  | | | | |
| Spread | 1.99 (1.41) | | | | | 1.78 (1.33) | | | | |  | | | | |
| Believe | 1.95 (1.40) | | | | | 2.03 (1.43) | | | | |  | | | | |
| Gender | 0.02 (0.16) | | | | | 0.01 (0.10) | | | | |  | | | | |
| Trial | 0.00 (0.01) | | | | | 0.00 (0.05) | | | | |  | | | | |
| *R^2^* |  |  |  |  |  |  |  |  |  |  |  |  |  |  |  |
| Conditional | .88 | | | | | .81 | | | | |  | | | | |
| Marginal | .34 | | | | | .22 | | | | |  | | | | |
| *Note.* CI = confidence interval. GCB = Generic Conspiracist Beliefs. Holm-corrected *p*-values are presented.  ^a^ 0 = male, 1 = female. | | | | | | | | | | | | | | | |

**Study 2**

| **Table S21** | | |
| --- | --- | --- |
| *Detailed Sample Demographics in the Two-Image Forced-Choice Task (Step 1)* | | |
| Variable | *n* | % |
| Gender |  |  |
| Male | 198 | 48.1 |
| Female | 203 | 49.3 |
| Missing | 11 | 2.7 |
| Ethnicity |  |  |
| White/Caucasian | 266 | 64.6 |
| African American | 55 | 13.3 |
| Hispanic | 37 | 9.0 |
| Asian | 30 | 7.3 |
| Native American | 7 | 1.7 |
| Jewish | 1 | 0.2 |
| Other | 16 | 3.9 |
| Political identification |  |  |
| Republican | 104 | 25.2 |
| Democrat | 141 | 34.2 |
| Independent | 152 | 36.9 |
| Other | 6 | 1.5 |
| No preference | 9 | 2.2 |
| Education |  |  |
| Less than high school degree | 7 | 1.7 |
| High school graduate (high school diploma or equivalent including GED) | 69 | 16.7 |
| Some college but no degree | 88 | 21.4 |
| Associate degree in college (2-year) | 48 | 11.7 |
| Bachelor’s degree in college (4-year) | 130 | 31.6 |
| Master’s degree | 55 | 13.3 |
| Doctoral degree | 5 | 1.2 |
| Professional degree (JD, MD) | 10 | 2.4 |
| Annual household income (before taxes) |  |  |
| Less than $15,000 | 23 | 5.6 |
| $15,000 – $24,999 | 26 | 6.3 |
| $25,000 – $34,999 | 38 | 9.2 |
| $35,000 – $49,999 | 55 | 13.3 |
| $50,000 – $74,999 | 78 | 18.9 |
| $75,000 – $99,999 | 69 | 16.7 |
| $100,000 – $149,999 | 89 | 21.6 |
| $150,000 or more | 34 | 8.3 |
| *Note. N* = 412 | | |

| **Table S22** | | |
| --- | --- | --- |
| *Detailed Sample Demographics in the Image-Rating Task (Step 2)* | | |
| Variable | *n* | % |
| Gender |  |  |
| Male | 89 | 48.6 |
| Female | 94 | 51.4 |
| Ethnicity |  |  |
| White/Caucasian | 148 | 80.9 |
| Black/African American | 23 | 12.6 |
| American Indian / Alaska Native | 1 | 0.5 |
| Chinese | 3 | 1.6 |
| Korean | 2 | 1.1 |
| Vietnamese | 3 | 1.6 |
| Japanese | 1 | 0.5 |
| Other | 2 | 1.1 |
| Hispanic/Latino/Spanish origin |  |  |
| No | 157 | 85.8 |
| Yes, Mexican/Mexican American/Chicano | 14 | 7.7 |
| Yes, Puerto Rican | 3 | 1.6 |
| Yes, Cuban | 4 | 2.2 |
| Yes, other Hispanic/Latino/Spanish origin (e.g., Salvadoran, Dominican) | 5 | 2.7 |
| Education |  |  |
| Less than high school degree | 5 | 2.7 |
| High school graduate (high school diploma or equivalent including GED) | 55 | 30.1 |
| Some college but no degree | 33 | 18.0 |
| Associate degree in college (2-year) | 20 | 10.9 |
| Bachelor’s degree in college (4-year) | 45 | 24.6 |
| Master’s degree | 20 | 10.9 |
| Doctoral degree | 4 | 2.2 |
| Professional degree (JD, MD) | 1 | 0.5 |
| Annual household income (before taxes) |  |  |
| Less than $15,000 | 10 | 5.5 |
| $15,000 – $24,999 | 23 | 12.6 |
| $25,000 – $34,999 | 16 | 8.7 |
| $35,000 – $49,999 | 23 | 12.6 |
| $50,000 – $74,999 | 38 | 20.8 |
| $75,000 – $99,999 | 26 | 14.2 |
| $100,000 – $149,999 | 28 | 15.3 |
| $150,000 or more | 19 | 10.4 |
| *Note. N* = 183 | | |

| **Table S23** | | | | | | | | | | | | | | | | |
| --- | --- | --- | --- | --- | --- | --- | --- | --- | --- | --- | --- | --- | --- | --- | --- | --- |
| *Bias Estimates From the Bootstrapped (N = 1,000) Models in Study 2* | | | | | | | | | | | | | | | | |
|  | Competence | | Morality | | Warmth | | Machiavellianism | | Narcissism | | Psychopathy | | Conspiracy intentions | | Contact willingness | |
|  | original | bias | original | bias | original | bias | original | bias | original | bias | original | bias | original | bias | original | bias |
| Intercept | 3.69 | 0.01 | 3.81 | 0.00 | 3.49 | 0.01 | 3.83 | 0.00 | 3.70 | 0.01 | 3.52 | 0.00 | 3.74 | -0.01 | 3.98 | 0.00 |
| Spread | -0.26 | 0.00 | -0.34 | 0.00 | -0.35 | 0.00 | 0.22 | 0.00 | 0.22 | 0.00 | 0.47 | 0.00 | 0.36 | 0.00 | -0.37 | 0.00 |
| Believe | -0.44 | 0.00 | -0.47 | 0.00 | -0.50 | 0.00 | 0.27 | 0.00 | 0.33 | 0.00 | 0.63 | 0.00 | 0.50 | 0.00 | -0.54 | 0.00 |
| Spread * Believe | -0.06 | 0.00 | -0.04 | 0.00 | -0.08 | 0.00 | 0.04 | 0.00 | 0.09 | 0.00 | 0.08 | 0.00 | 0.09 | 0.01 | -0.09 | 0.00 |
| *Note.* Table displays the original coefficients from the models and bias estimates from the bootstrap. | | | | | | | | | | | | | | | | |

***Pre-Test of Base Images*** Given that we wanted to explore what gender individuals who engage with conspiracy theories most resembles it was important to utilize a relative “gender-neutral” base image in order to minimize a potential bias. In a pre-test we recruited 50 participants (50% women; *M_age_* = 39.24, *SD_age_* = 12.95) and asked them to rate 11 facial images with gradual variation (i.e., 10%) in the opacity of the aggregated male and female base images from the Karolinska Facial Database. The opacity was adjusted in increments of 10% for each image. For instance, the first image was the aggregated male facial base image, whereas the second image had the female base image superimposed with 10% opacity while the male base image had 90% opacity. In the third base image the female base image had 20% opacity, whereas the male base image had 80% opacity. These 10% adjustments in opacity were repeated until the last image which was the aggregated female base image with 100% opacity whereas the male base image had 0% opacity. The participants were asked “to what extent does the individual in the image look like a man or a woman”, and the images were rated from 1 (*definitely a man*) to 11 (*definitely a woman*). The base image that received scores closest to the midpoint of the scale (i.e., 6) was used as base image in the main study. The results indicated that the base image with 40% opacity of the male image and 60% opacity of the female image was perceived as closest to the midpoint of the scale (*M* = 6.44, *SD* = 2.32; Median = 6.50).

***Section 2: Trait Descriptions in the Image-Rating Task***

***Competence:*** In each trial, we would like you to evaluate how competent the individual appears. By "competence," we refer to the individual's perceived capability, skillfulness, intelligence, and confidence.

***Morality:*** In each trial, we would like you to evaluate how moral the individual appears. By "moral," we refer to the individual's perceived honesty, sincerity, and trustworthiness.

***Warmth:*** In each trial, we would like you to evaluate how warm the individual appears. By "warmth," we mean the individual's perceived friendliness, likability, and good-natured demeanor.

***Narcissism:*** In each trial, we would like you to evaluate how narcissistic the individual appears. By "narcissism," we refer to an individual's excessive preoccupation with themselves, characterized by a heightened sense of self-importance, a need for admiration, and a lack of empathy for others.

***Machiavellianism:*** In each trial, we would like you to evaluate how manipulative the individual appears. By "manipulative," we mean an individual's tendency to deceive, lie, exploit others, and use flattery strategically to achieve their own goals.

***Psychopathy:*** In each trial, we would like you to evaluate how psychopathic the individual appears. By "psychopathy," we refer to an individual's callousness, insensitivity, and marked lack of remorse, combined with a disregard for morality and the consequences of their actions, often accompanied by cynicism.

| **Figure S1** |
| --- |
| *Attention Check Example From Study 2* |
| **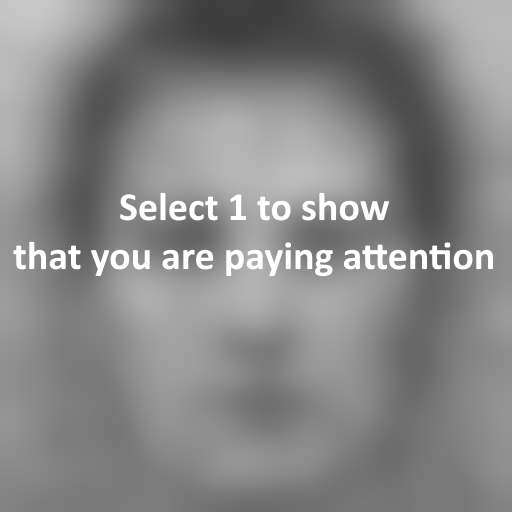** |
| *Note.* Randomly interspersed between the images to be rated we included three attention checks which asked the participants to select a specific response option. Participants who failed two or more out of three total attention checks were excluded from the final sample. |

| **Table S24** | | | | | | | | | | | | | | | |
| --- | --- | --- | --- | --- | --- | --- | --- | --- | --- | --- | --- | --- | --- | --- | --- |
| *Study 2 Results of Multi-Level Linear Models: Examining the Fixed Effects of Spread and Believe, and Their Interaction on Dependent Variables, with Control for Random Intercepts and Effects* | | | | | | | | | | | | | | | |
|  | Competence | | | | | Morality | | | | | Warmth | | | | |
| Effect | *B*  [95% CI] | *SE* | *t* | *df* | *p* | *B*  [95% CI] | *SE* | *t* | *df* | *p* | *B*  [95% CI] | *SE* | *t* | *df* | *p* |
| Fixed effects |  |  |  |  |  |  |  |  |  |  |  |  |  |  |  |
| (Intercept) | 4.03  [3.62, 4.46] | 0.22 | 18.23 | 22.91 | < .001 | 4.21  [3.93, 4.51] | 0.15 | 27.56 | 32.58 | < .001 | 3.90  [3.52, 4.28] | 0.20 | 19.36 | 27.39 | < .001 |
| Spread | -0.24  [-0.39, -0.06] | 0.09 | -2.67 | 218.16 | .008 | -0.32  [-0.52, -0.13] | 0.10 | -3.27 | 190.28 | .001 | -0.32  [-0.54, -0.11] | 0.11 | -2.92 | 263.69 | .004 |
| Believe | -0.41  [-0.61, -0.22] | 0.10 | -3.99 | 97.03 | < .001 | -0.45  [-0.67, -0.25] | 0.10 | -4.34 | 151.45 | < .001 | -0.46  [-0.71, -0.24] | 0.12 | -3.99 | 172.34 | < .001 |
| Spread * believe | -0.06  [-0.28, 0.15] | 0.11 | -0.49 | 410.66 | .621 | -0.04  [-0.27, 0.21] | 0.12 | -0.30 | 410.72 | .763 | -0.08  [-0.34, 0.19] | 0.14 | -0.56 | 410.78 | .573 |
| Random Effects | Variance (*SD*) | | | | | Variance (*SD*) | | | | | Variance (*SD*) | | | | |
| Participants intercept | 0.90 (0.95) | | | | | 0.44 (0.67) | | | | | 0.74 (0.86) | | | | |
| Spread | 0.03 (0.17) | | | | | 0.06 (0.24) | | | | | 0.05 (0.21) | | | | |
| Believe | 0.08 (0.29) | | | | | 0.08 (0.28) | | | | | 0.08 (0.28) | | | | |
| Trial | 0.27 (0.52) | | | | | 0.32 (0.57) | | | | | 0.44 (0.67) | | | | |
| *R^2^* |  |  |  |  |  |  |  |  |  |  |  |  |  |  |  |
| Conditional | .53 | | | | | .49 | | | | | .65 | | | | |
| Marginal | .03 | | | | | .05 | | | | | .04 | | | | |
| *Note.* CI = confidence interval. | | | | | | | | | | | | | | | |

| **Table S25** | | | | | | | | | | | | | | | |
| --- | --- | --- | --- | --- | --- | --- | --- | --- | --- | --- | --- | --- | --- | --- | --- |
| *Study 2 Results of Multi-Level Linear Models: Examining the Fixed Effects of Spread and Believe, and Their Interaction on Dependent Variables, with Control for Random Intercepts and Effects* | | | | | | | | | | | | | | | |
|  | Machiavellianism | | | | | Narcissism | | | | | Psychopathy | | | | |
| Effect | *B*  [95% CI] | *SE* | *t* | *df* | *p* | *B*  [95% CI] | *SE* | *t* | *df* | *p* | *B*  [95% CI] | *SE* | *t* | *df* | *p* |
| Fixed effects |  |  |  |  |  |  |  |  |  |  |  |  |  |  |  |
| (Intercept) | 3.60  [3.28, 3.95] | 0.17 | 21.21 | 21.94 | < .001 | 3.44  [3.18, 3.70] | 0.14 | 24.93 | 25.56 | < .001 | 2.99  [2.53, 3.50] | 0.25 | 12.17 | 25.45 | < .001 |
| Spread | 0.19  [0.04, 0.33] | 0.07 | 2.66 | 71.23 | .010 | 0.17  [0.02, 0.34] | 0.08 | 2.14 | 92.04 | .035 | 0.43  [0.20, 0.69] | 0.12 | 3.55 | 334.47 | < .001 |
| Believe | 0.24  [0.05, 0.42] | 0.09 | 2.63 | 41.53 | .012 | 0.29  [0.09, 0.51] | 0.11 | 2.60 | 41.77 | .013 | 0.60  [0.34, 0.85] | 0.13 | 4.66 | 252.12 | < .001 |
| Spread * believe | 0.04  [-0.11, 0.19] | 0.08 | 0.54 | 410.36 | .592 | 0.09  [-0.08, 0.26] | 0.09 | 0.97 | 410.47 | .335 | 0.08  [-0.23, 0.38] | 0.16 | 0.47 | 410.91 | .639 |
| Random Effects | Variance (*SD*) | | | | | Variance (*SD*) | | | | | Variance (*SD*) | | | | |
| Participants intercept | 0.54 (0.74) | | | | | 0.35 (0.59) | | | | | 1.06 (1.03) | | | | |
| Spread | 0.05 (0.21) | | | | | 0.05 (0.22) | | | | | 0.03 (0.17) | | | | |
| Believe | 0.11 (0.33) | | | | | 0.17 (0.41) | | | | | 0.06 (0.24) | | | | |
| Trial | 0.10 (0.32) | | | | | 0.14 (0.37) | | | | | 0.59 (0.77) | | | | |
| *R^2^* |  |  |  |  |  |  |  |  |  |  |  |  |  |  |  |
| Conditional | .36 | | | | | .30 | | | | | .58 | | | | |
| Marginal | .02 | | | | | .02 | | | | | .05 | | | | |
| *Note.* CI = confidence interval. | | | | | | | | | | | | | | | |

| **Table S26** | | | | | | | | | | | | | | | |
| --- | --- | --- | --- | --- | --- | --- | --- | --- | --- | --- | --- | --- | --- | --- | --- |
| *Study 2 Results of Multi-Level Linear Models: Examining the Fixed Effects of Spread and Believe, and Their Interaction on Dependent Variables, with Control for Random Intercepts and Effects* | | | | | | | | | | | | | | | |
|  | Conspiracy Intentions | | | | | Contact Willingness | | | | |  | | | | |
| Effect | *B*  [95% CI] | *SE* | *t* | *df* | *p* | *B*  [95% CI] | *SE* | *t* | *df* | *p* |  |  |  |  |  |
| Fixed effects |  |  |  |  |  |  |  |  |  |  |  |  |  |  |  |
| (Intercept) | 3.33  [3.02, 3.66] | 0.17 | 19.93 | 27.51 | < .001 | 4.41  [4.16, 4.67] | 0.13 | 33.38 | 37.52 | < .001 |  |  |  |  |  |
| Spread | 0.32  [0.11, 0.52] | 0.10 | 3.09 | 295.61 | .002 | -0.32  [-0.51, -0.12] | 0.10 | -3.31 | 297.18 | .001 |  |  |  |  |  |
| Believe | 0.46  [0.24, 0.67] | 0.11 | 4.17 | 196.72 | < .001 | -0.49  [-0.69, -0.29] | 0.11 | -4.53 | 159.35 | < .001 |  |  |  |  |  |
| Spread * believe | 0.09  [-0.18, 0.34] | 0.14 | 0.64 | 410.97 | .524 | -0.09  [-0.34, 0.15] | 0.12 | -0.75 | 410.91 | .457 |  |  |  |  |  |
| Random Effects | Variance (*SD*) | | | | | Variance (*SD*) | | | | |  | | | | |
| Participants intercept | 0.43 (0.66) | | | | | 0.28 (0.54) | | | | |  | | | | |
| Spread | 0.02 (0.15) | | | | | 0.03 (0.17) | | | | |  | | | | |
| Believe | 0.05 (0.22) | | | | | 0.08 (0.28) | | | | |  | | | | |
| Trial | 0.41 (0.64) | | | | | 0.36 (0.60) | | | | |  | | | | |
| *R^2^* |  |  |  |  |  |  |  |  |  |  |  |  |  |  |  |
| Conditional | .41 | | | | | .50 | | | | |  | | | | |
| Marginal | .04 | | | | | .06 | | | | |  | | | | |
| *Note.* CI = confidence interval. | | | | | | | | | | | | | | | |

| **Table S27** | | | | | | | | | | | | | | | |
| --- | --- | --- | --- | --- | --- | --- | --- | --- | --- | --- | --- | --- | --- | --- | --- |
| *Study 2 Results of Multi-Level Linear Models: Examining the Fixed Effects of Spread and Believe on Dependent Variables, with Control for Random Intercepts and Effects* | | | | | | | | | | | | | | | |
|  | Competence | | | | | Morality | | | | | Warmth | | | | |
| Effect | *B*  [95% CI] | *SE* | *t* | *df* | *p* | *B*  [95% CI] | *SE* | *t* | *df* | *p* | *B*  [95% CI] | *SE* | *t* | *df* | *p* |
| Fixed effects |  |  |  |  |  |  |  |  |  |  |  |  |  |  |  |
| (Intercept) | 3.69  [3.23, 4.12] | 0.20 | 18.50 | 20.69 | < .001 | 3.81  [3.56, 4.08] | 0.14 | 28.10 | 25.11 | < .001 | 3.49  [3.04, 3.97] | 0.20 | 17.69 | 22.22 | < .001 |
| Spread | -0.26  [-0.37, -0.13] | 0.07 | -3.93 | 101.11 | < .001 | -0.34  [-0.46, -0.18] | 0.08 | -4.37 | 91.61 | < .001 | -0.35  [-0.51, -0.17] | 0.08 | -4.28 | 133.26 | < .001 |
| Believe | -0.44  [-0.57, -0.29] | 0.09 | -5.16 | 48.08 | < .001 | -0.47  [-0.64, -0.33] | 0.08 | -5.62 | 72.59 | < .001 | -0.50  [-0.67, -0.32] | 0.09 | -5.46 | 81.04 | < .001 |
| Spread * believe | -0.06  [-0.29, 0.19] | 0.11 | -0.49 | 410.66 | .621 | -0.04  [-0.24, 0.16] | 0.12 | -0.30 | 410.72 | .763 | -0.08  [-0.30, 0.18] | 0.14 | -0.56 | 410.78 | .573 |
| Random Effects | Variance (*SD*) | | | | | Variance (*SD*) | | | | | Variance (*SD*) | | | | |
| Participants intercept | 0.79 (0.88) | | | | | 0.40 (0.64) | | | | | 0.79 (0.89) | | | | |
| Spread | 0.03 (0.17) | | | | | 0.06 (0.24) | | | | | 0.05 (0.21) | | | | |
| Believe | 0.08 (0.29) | | | | | 0.08 (0.28) | | | | | 0.08 (0.28) | | | | |
| Trial | 0.27 (0.52) | | | | | 0.32 (0.57) | | | | | 0.44 (0.67) | | | | |
| *R^2^* |  |  |  |  |  |  |  |  |  |  |  |  |  |  |  |
| Conditional | .53 | | | | | .49 | | | | | .65 | | | | |
| Marginal | .03 | | | | | .05 | | | | | .04 | | | | |
| *Note.* CI = confidence interval. Variables are effect coded. | | | | | | | | | | | | | | | |

| **Table S28** | | | | | | | | | | | | | | | |
| --- | --- | --- | --- | --- | --- | --- | --- | --- | --- | --- | --- | --- | --- | --- | --- |
| *Study 2 Results of Multi-Level Linear Models: Examining the Fixed Effects of Spread and Believe on Dependent Variables, with Control for Random Intercepts and Effects* | | | | | | | | | | | | | | | |
|  | Machiavellianism | | | | | Narcissism | | | | | Psychopathy | | | | |
| Effect | *B*  [95% CI] | *SE* | *t* | *df* | *p* | *B*  [95% CI] | *SE* | *t* | *df* | *p* | *B*  [95% CI] | *SE* | *t* | *df* | *p* |
| Fixed effects |  |  |  |  |  |  |  |  |  |  |  |  |  |  |  |
| (Intercept) | 3.83  [3.61, 4.11] | 0.16 | 24.44 | 20.41 | < .001 | 3.70  [3.45, 3.99] | 0.15 | 24.30 | 21.62 | < .001 | 3.52  [3.14, 4.01] | 0.24 | 14.87 | 21.07 | < .001 |
| Spread | 0.22  [0.10, 0.31] | 0.06 | 3.52 | 36.83 | .001 | 0.22  [0.05, 0.34] | 0.07 | 3.29 | 43.94 | .002 | 0.47  [0.30, 0.62] | 0.09 | 5.25 | 196.44 | < .001 |
| Believe | 0.27  [0.12, 0.40] | 0.08 | 3.19 | 27.14 | .003 | 0.33  [0.11, 0.50] | 0.10 | 3.33 | 27.87 | .002 | 0.63  [0.47, 0.82] | 0.10 | 6.58 | 120.52 | < .001 |
| Spread * believe | 0.04  [-0.08, 0.19] | 0.08 | 0.54 | 410.36 | .592 | 0.09  [-0.08, 0.28] | 0.09 | 0.97 | 410.47 | .338 | 0.08  [-0.34, 0.48] | 0.16 | 0.47 | 410.91 | .639 |
| Random Effects | Variance (*SD*) | | | | | Variance (*SD*) | | | | | Variance (*SD*) | | | | |
| Participants intercept | 0.48 (0.70) | | | | | 0.47 (0.69) | | | | | 1.08 (1.04) | | | | |
| Spread | 0.05 (0.21) | | | | | 0.05 (0.22) | | | | | 0.03 (0.17) | | | | |
| Believe | 0.11 (0.33) | | | | | 0.17 (0.41) | | | | | 0.06 (0.24) | | | | |
| Trial | 0.10 (0.32) | | | | | 0.14 (0.37) | | | | | 0.59 (0.77) | | | | |
| *R^2^* |  |  |  |  |  |  |  |  |  |  |  |  |  |  |  |
| Conditional | .36 | | | | | .30 | | | | | .58 | | | | |
| Marginal | .02 | | | | | .02 | | | | | .04 | | | | |
| *Note.* CI = confidence interval. Variables are effect coded. | | | | | | | | | | | | | | | |

| **Table S29** | | | | | | | | | | | | | | | |
| --- | --- | --- | --- | --- | --- | --- | --- | --- | --- | --- | --- | --- | --- | --- | --- |
| *Study 2 Results of Multi-Level Linear Models: Examining the Fixed Effects of Spread and Believe on Dependent Variables, with Control for Random Intercepts and Effects* | | | | | | | | | | | | | | | |
|  | Conspiracy Intentions | | | | | Contact Willingness | | | | |  | | | | |
| Effect | *B*  [95% CI] | *SE* | *t* | *df* | *p* | *B*  [95% CI] | *SE* | *t* | *df* | *p* |  |  |  |  |  |
| Fixed effects |  |  |  |  |  |  |  |  |  |  |  |  |  |  |  |
| (Intercept) | 3.74  [3.49, 3.98] | 0.13 | 28.06 | 21.31 | < .001 | 3.98  [3.79, 4.23] | 0.14 | 28.88 | 24.16 | < .001 |  |  |  |  |  |
| Spread | 0.36  [0.20, 0.51] | 0.08 | 4.74 | 155.63 | < .001 | -0.37  [-0.51, -0.23] | 0.07 | -5.05 | 158.09 | < .001 |  |  |  |  |  |
| Believe | 0.50  [0.35, 0.68] | 0.08 | 5.96 | 88.21 | < .001 | -0.54  [-0.70, -0.32] | 0.09 | -6.24 | 75.18 | < .001 |  |  |  |  |  |
| Spread * believe | 0.09  [-0.13, 0.35] | 0.14 | 0.64 | 410.97 | .524 | -0.09  [-0.36, 0.17] | 0.12 | -0.75 | 410.91 | .457 |  |  |  |  |  |
| Random Effects | Variance (*SD*) | | | | | Variance (*SD*) | | | | |  | | | | |
| Participants intercept | 0.32 (0.56) | | | | | 0.40 (0.63) | | | | |  | | | | |
| Spread | 0.02 (0.15) | | | | | 0.03 (0.17) | | | | |  | | | | |
| Believe | 0.05 (0.22) | | | | | 0.08 (0.28) | | | | |  | | | | |
| Trial | 0.41 (0.64) | | | | | 0.36 (0.60) | | | | |  | | | | |
| *R^2^* |  |  |  |  |  |  |  |  |  |  |  |  |  |  |  |
| Conditional | .41 | | | | | .50 | | | | |  | | | | |
| Marginal | .04 | | | | | .06 | | | | |  | | | | |
| *Note.* CI = confidence interval. Variables are effect coded. | | | | | | | | | | | | | | | |

| **Figure S2** | | | |
| --- | --- | --- | --- |
| *Estimated Marginal Means of all Combinations of Spread and Believe on Perceived Individual Differences, Contact Willingness, and Conspiracy Intentions* | | | |
| 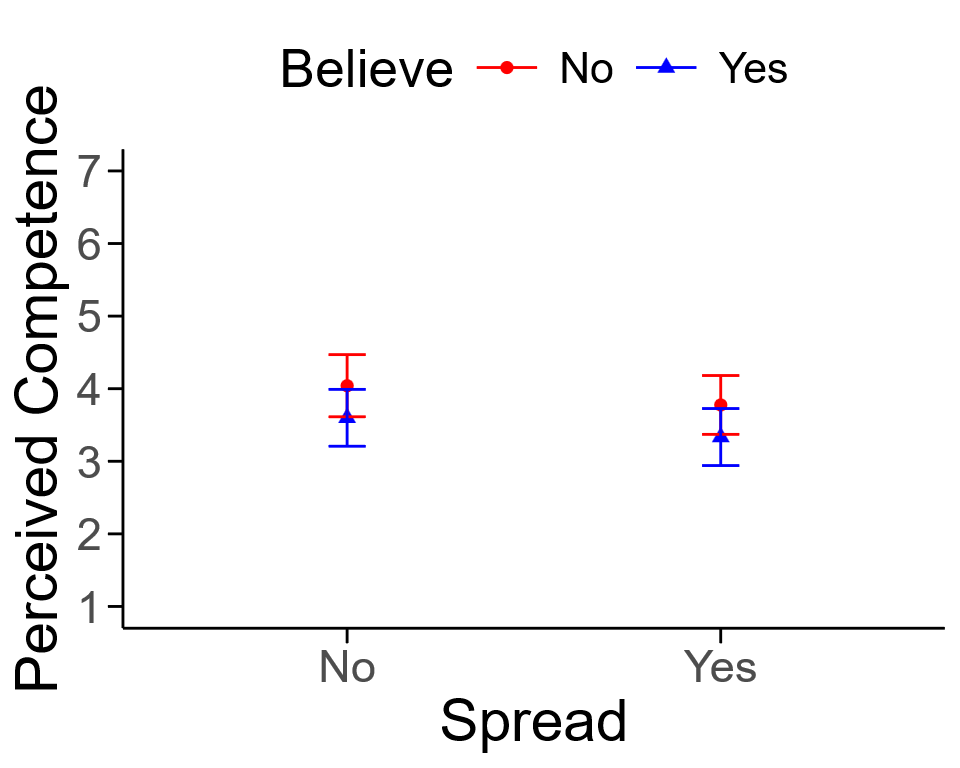 | 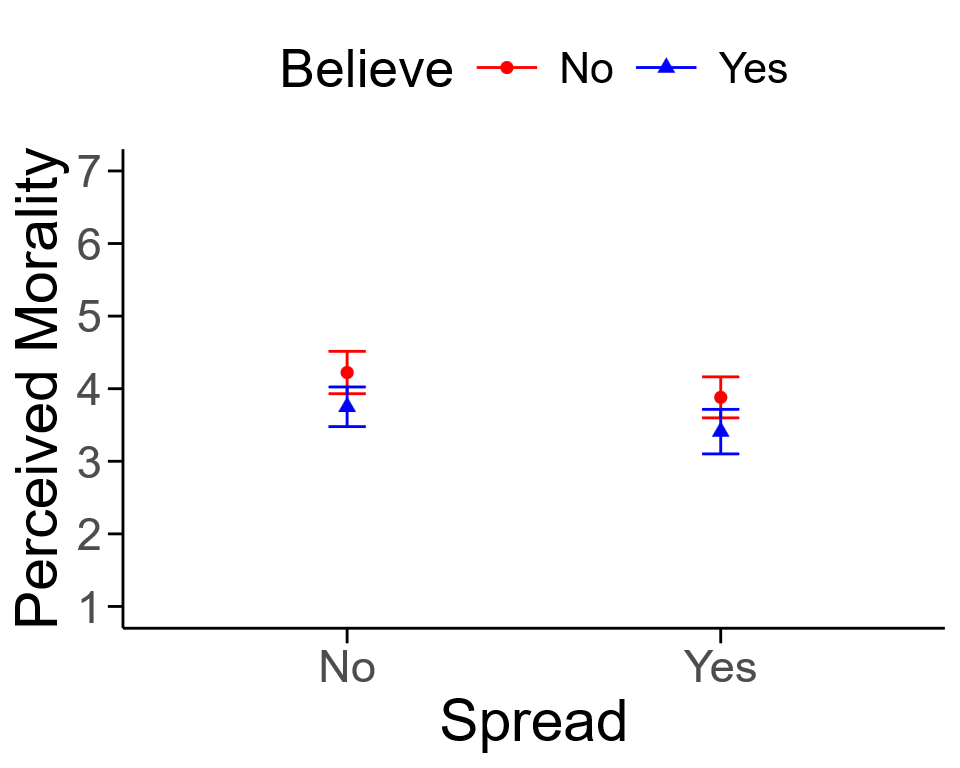 | | 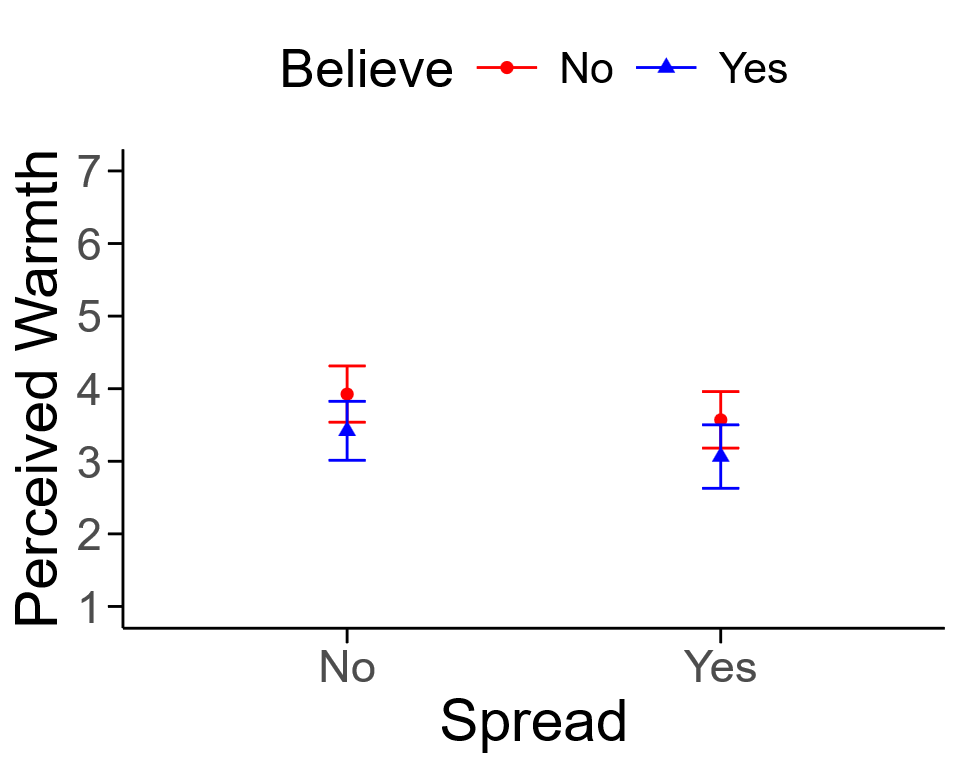 |
| 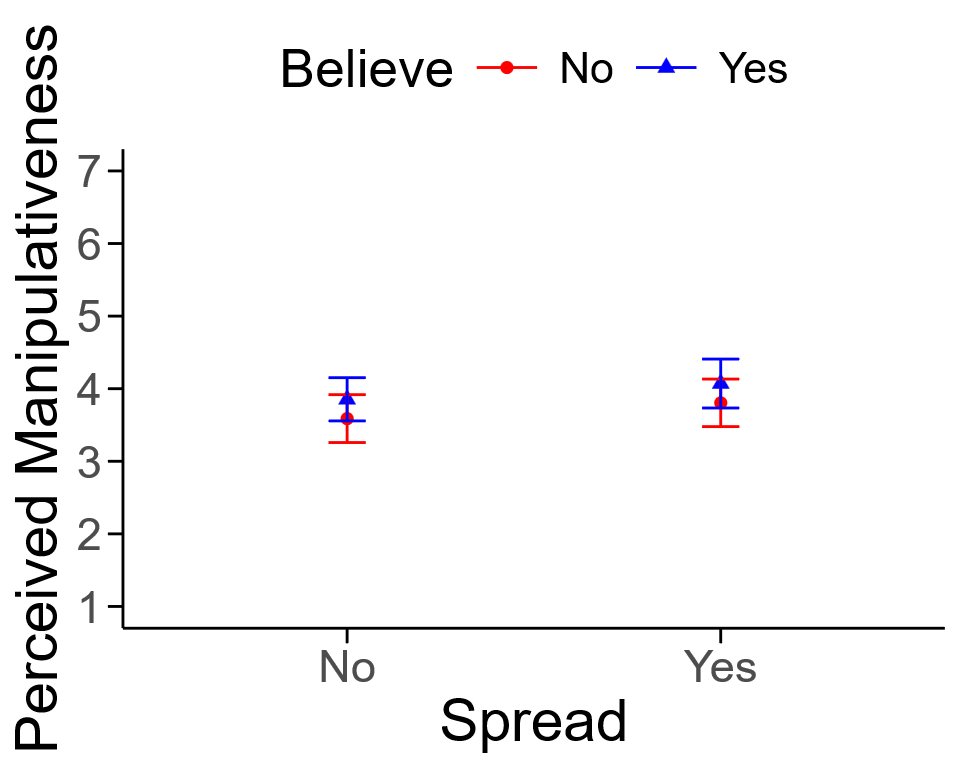 | 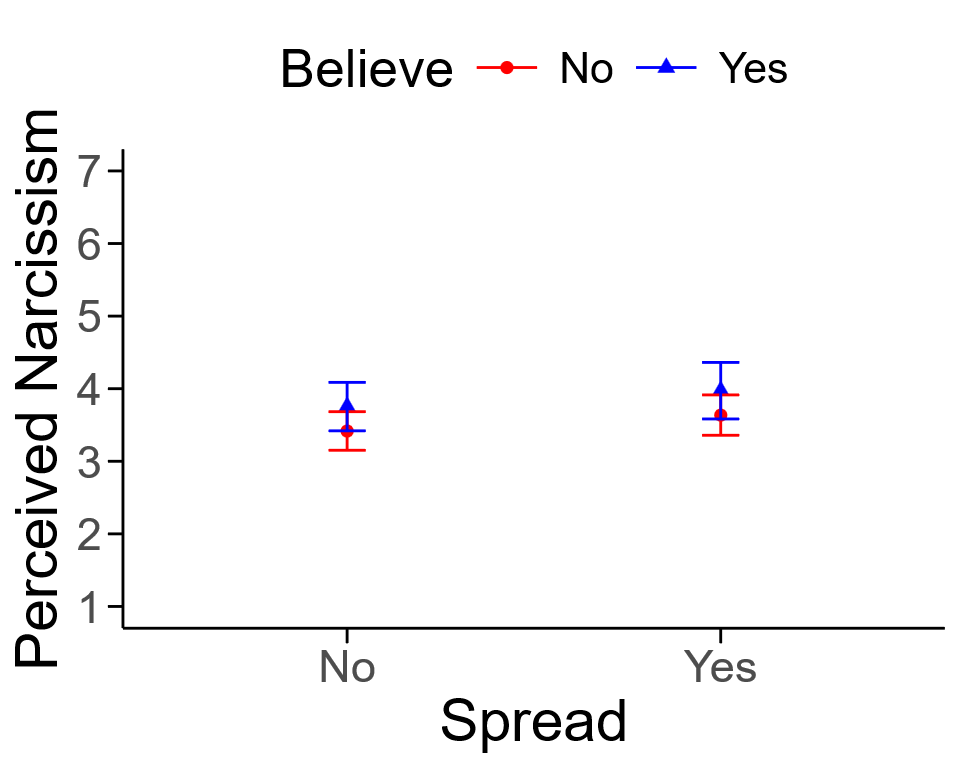 | | 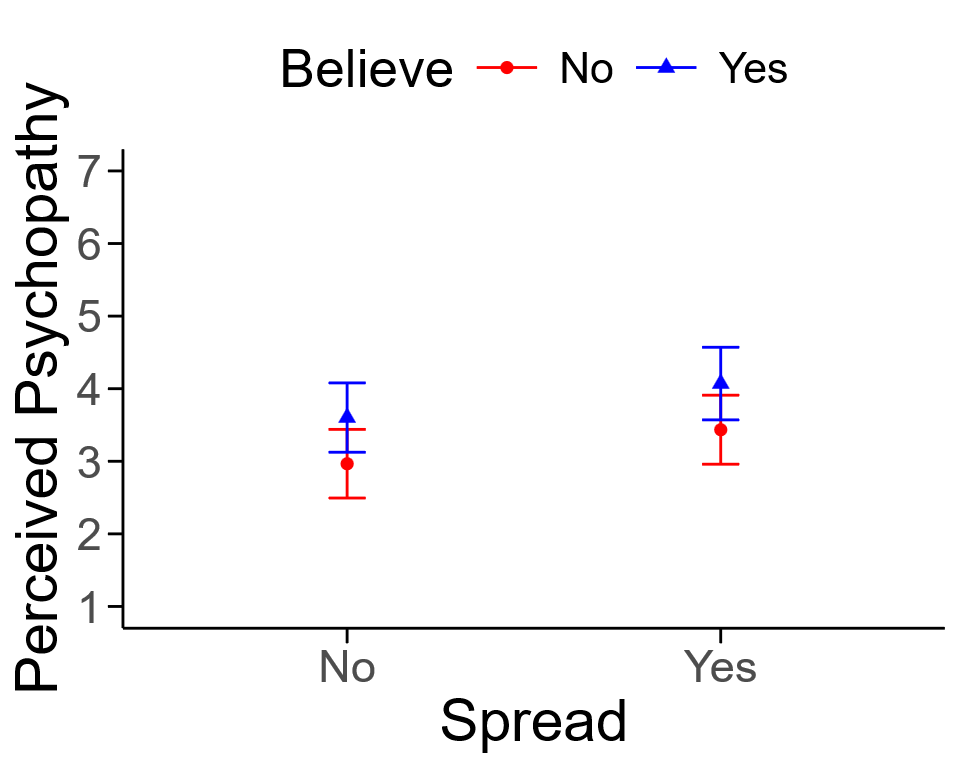 |
| 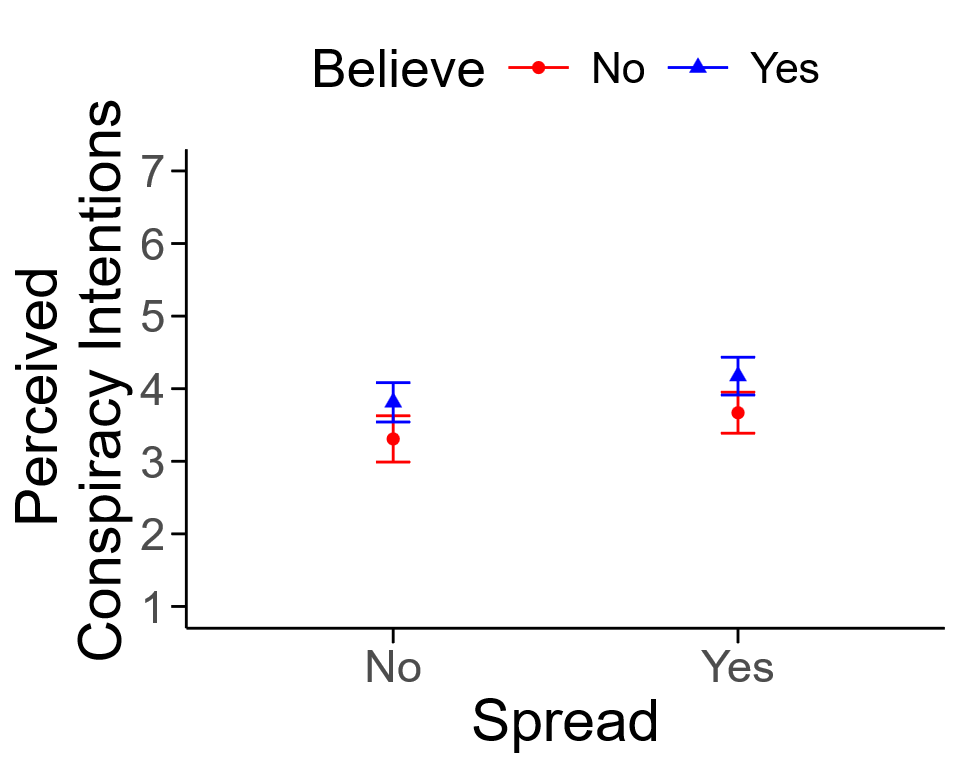 | | 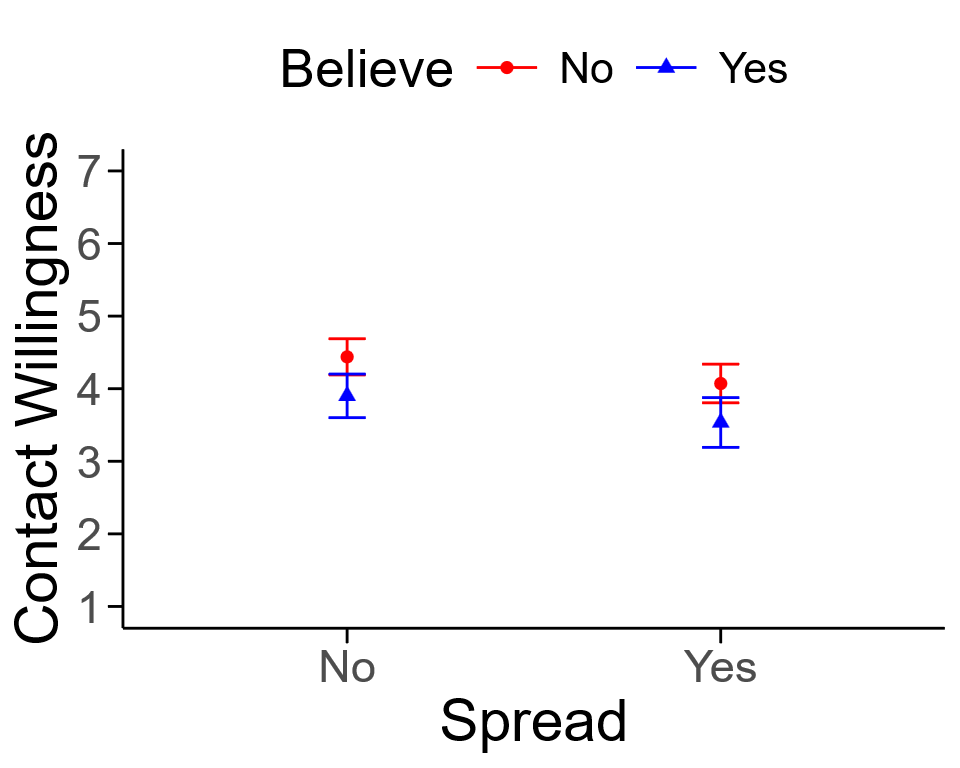 | |
| *Note*. Error bars represent 95% confidence intervals of estimated mean values. | | | |

| **Table S30** | | | | | | | | | | | | | | | |
| --- | --- | --- | --- | --- | --- | --- | --- | --- | --- | --- | --- | --- | --- | --- | --- |
| *Results of Multi-Level Linear Models: Investigating Separate two-way Interactions of Spread and Believe With Participants’ Conspiracy Beliefs and Political Orientation* | | | | | | | | | | | | | | | |
|  | Competence | | | | | Morality | | | | | Warmth | | | | |
| Effect | *B*  [95% CI] | *SE* | *t* | *df* | *p* | *B*  [95% CI] | *SE* | *t* | *df* | *p* | *B*  [95% CI] | *SE* | *t* | *df* | *p* |
| Fixed effects |  |  |  |  |  |  |  |  |  |  |  |  |  |  |  |
| (Intercept) | 4.26  [3.75, 4.90] | 0.27 | 15.87 | 49.35 | < .001 | 4.47  [4.02, 4.83] | 0.22 | 20.18 | 127.44 | < .001 | 4.12  [3.53, 4.64] | 0.27 | 15.02 | 87.96 | < .001 |
| Spread | -0.40  [-0.79, -0.11] | 0.18 | -2.28 | 414.89 | .223 | -0.47  [-0.75, -0.03] | 0.19 | -2.47 | 417.21 | .124 | -0.46  [-0.91, -0.08] | 0.22 | -2.14 | 424.91 | .299 |
| Believe | -0.93  [-1.29, -0.61] | 0.18 | -5.06 | 354.13 | < .001 | -1.01  [-1.38, -0.63] | 0.19 | -5.32 | 401.91 | < .001 | -1.02  [-1.42, -0.62] | 0.22 | -4.65 | 411.31 | < .001 |
| GCB | -0.06  [-0.13, 0.01] | 0.04 | -1.50 | 410.65 | .948 | -0.05  [-0.12, 0.02] | 0.04 | -1.31 | 410.69 | > .999 | -0.03  [-0.11, 0.06] | 0.04 | -0.77 | 410.77 | > .999 |
| Political orientation | -0.00  [-0.03, 0.04] | 0.02 | -0.03 | 410.65 | > .999 | -0.01  [-0.04, 0.03] | 0.02 | -0.37 | 410.69 | > .999 | -0.01  [-0.06, 0.03] | 0.02 | -0.39 | 410.77 | > .999 |
| Political orientation^2 | 6.47  [-19.92, 28.66] | 14.19 | 0.46 | 410.65 | > .999 | 1.02  [-25.35, 26.62] | 14.89 | 0.07 | 410.69 | > .999 | -0.35  [-24.78, 43.45] | 17.22 | -0.02 | 410.77 | > .999 |
| GCB * Spread | 0.04  [-0.02, 0.12] | 0.04 | 1.06 | 410.65 | > .999 | 0.03  [-0.06, 0.10] | 0.04 | 0.60 | 410.69 | > .999 | 0.03  [-0.07, 0.12] | 0.05 | 0.53 | 410.77 | > .999 |
| GCB * Believe | 0.08  [0.01, 0.15] | 0.04 | 2.03 | 410.65 | .391 | 0.06  [-0.02, 0.13] | 0.04 | 1.42 | 410.69 | > .999 | 0.05  [-0.03, 0.15] | 0.05 | 1.10 | 410.77 | > .999 |
| Political orientation  * Spread | -0.01  [-0.05, 0.03] | 0.02 | -0.29 | 410.65 | > .999 | 0.00  [-0.04, 0.04] | 0.02 | 0.17 | 410.69 | > .999 | -0.00  [-0.04, 0.06] | 0.02 | -0.01 | 410.77 | > .999 |
| Political orientation  * Believe | 0.03  [-0.00, 0.07] | 0.02 | 1.67 | 410.65 | .762 | 0.06  [0.01, 0.10] | 0.02 | 2.75 | 410.69 | .063 | 0.06  [0.01, 0.10] | 0.02 | 2.36 | 410.77 | .186 |
| Political orientation^2 * Spread | -18.49  [-48.64, 9.70] | 15.59 | -1.19 | 410.65 | > .999 | -19.81  [-52.61, 6.52] | 16.36 | -1.21 | 410.69 | > .999 | -16.22  [-56.41, 13.67] | 18.92 | -0.86 | 410.77 | > .999 |
| Political orientation^2 * Believe | 14.76  [-9.42, 45.55] | 15.37 | 0.96 | 410.65 | > .999 | 32.23  [3.57, 60.68] | 16.13 | 2.00 | 410.69 | .370 | 36.05  [-13.35, 69.01] | 18.65 | 1.93 | 410.77 | .432 |
| Random Effects | Variance (*SD*) | | | | | Variance (*SD*) | | | | | Variance (*SD*) | | | | |
| Participants intercept | 0.90 (0.95) | | | | | 0.44 (0.67) | | | | | 0.74 (0.86) | | | | |
| Spread | 0.03 (0.17) | | | | | 0.06 (0.24) | | | | | 0.05 (0.21) | | | | |
| Believe | 0.08 (0.29) | | | | | 0.08 (0.28) | | | | | 0.81 (0.28) | | | | |
| Trial | 0.26 (0.51) | | | | | 0.30 (0.55) | | | | | 0.42 (0.65) | | | | |
| *R^2^* |  |  |  |  |  |  |  |  |  |  |  |  |  |  |  |
| Conditional | .53 | | | | | .49 | | | | | .65 | | | | |
| Marginal | .03 | | | | | .06 | | | | | .05 | | | | |
| *Note.* CI = confidence interval. GCB = Generic Conspiracist Beliefs. Holm-corrected *p*-values are presented. | | | | | | | | | | | | | | | |

| **Table S31** | | | | | | | | | | | | | | | |
| --- | --- | --- | --- | --- | --- | --- | --- | --- | --- | --- | --- | --- | --- | --- | --- |
| *Results of Multi-Level Linear Models: Investigating Separate two-way Interactions of Spread and Believe With Participants’ Conspiracy Beliefs and Political Orientation* | | | | | | | | | | | | | | | |
|  | Machiavellianism | | | | | Narcissism | | | | | Psychopathy | | | | |
| Effect | *B*  [95% CI] | *SE* | *t* | *df* | *p* | *B*  [95% CI] | *SE* | *t* | *df* | *p* | *B*  [95% CI] | *SE* | *t* | *df* | *p* |
| Fixed effects |  |  |  |  |  |  |  |  |  |  |  |  |  |  |  |
| (Intercept) | 3.52  [3.24, 3.84] | 0.20 | 17.54 | 42.39 | < .001 | 3.35  [3.04, 3.74] | 0.19 | 17.97 | 80.55 | < .001 | 2.63  [2.03, 3.22] | 0.33 | 7.98 | 78.43 | < .001 |
| Spread | 0.30  [0.01, 0.51] | 0.13 | 2.31 | 314.81 | .217 | 0.23  [-0.08, 0.53] | 0.15 | 1.53 | 354.39 | > .999 | 0.72  [0.26, 1.14] | 0.25 | 2.86 | 420.36 | .045 |
| Believe | 0.49  [0.24, 0.73] | 0.14 | 3.49 | 175.26 | .007 | 0.68  [0.39, 0.97] | 0.17 | 4.07 | 169.80 | < .001 | 1.14  [0.70, 1.51] | 0.25 | 4.50 | 420.80 | < .001 |
| GCB | 0.01  [-0.05, 0.05] | 0.03 | 0.56 | 410.35 | > .999 | 0.03  [-0.03, 0.07] | 0.03 | 1.15 | 410.46 | > .999 | 0.05  [-0.02, 0.15] | 0.05 | 0.98 | 410.91 | > .999 |
| Political orientation | 0.00  [-0.02, 0.03] | 0.01 | 0.06 | 410.35 | > .999 | -0.01  [-0.04, 0.02] | 0.02 | -0.80 | 410.46 | > .999 | 0.02  [-0.03, 0.07] | 0.03 | 0.88 | 410.91 | > .999 |
| Political orientation^2 | -10.49  [-30.30, 6.40] | 9.93 | -1.06 | 410.35 | > .999 | 10.87  [-14.22, 34.05] | 11.63 | 0.94 | 410.46 | > .999 | 0.73  [-38.60, 42.10] | 20.37 | 0.04 | 410.91 | > .999 |
| GCB * Spread | -0.02  [-0.07, 0.04] | 0.03 | -0.53 | 410.35 | > .999 | -0.02  [-0.08, 0.06] | 0.03 | -0.50 | 410.46 | > .999 | -0.05  [-0.15, 0.05] | 0.06 | -0.84 | 410.91 | > .999 |
| GCB * Believe | -0.03  [-0.08, 0.02] | 0.03 | -1.12 | 410.35 | > .999 | -0.06  [-0.11, 0.01] | 0.03 | -1.69 | 410.46 | .909 | -0.04  [-0.16, 0.07] | 0.06 | -0.67 | 410.91 | > .999 |
| Political orientation  * Spread | -0.00  [-0.02, 0.02] | 0.01 | -0.29 | 410.35 | > .999 | 0.01  [-0.02, 0.04] | 0.02 | 0.63 | 410.46 | > .999 | -0.01  [-0.06, 0.04] | 0.03 | -0.36 | 410.91 | > .999 |
| Political orientation  * Believe | -0.02  [-0.04, 0.01] | 0.01 | -1.41 | 410.35 | > .999 | -0.03  [-0.06, -0.00] | 0.02 | -1.58 | 410.46 | > .999 | -0.07  [-0.12, -0.01] | 0.03 | -2.30 | 410.91 | .197 |
| Political orientation^2 * Spread | 18.45  [-1.02, 40.36] | 10.90 | 1.69 | 410.35 | .822 | -8.31  [-36.15, 17.33] | 12.77 | -0.65 | 410.46 | > .999 | 15.47  [-29.30, 57.78] | 22.38 | 0.69 | 410.91 | > .999 |
| Political orientation^2 * Believe | -11.75  [-31.60, 7.89] | 10.75 | -1.09 | 410.35 | > .999 | -18.04  [-42.17, 6.83] | 12.59 | -1.43 | 410.46 | > .999 | -37.93  [-78.21, 8.78] | 22.06 | -1.72 | 410.91 | .691 |
| Random Effects | Variance (*SD*) | | | | | Variance (*SD*) | | | | | Variance (*SD*) | | | | |
| Participants intercept | 0.54 (0.74) | | | | | 0.35 (0.59) | | | | | 1.06 (1.02) | | | | |
| Spread | 0.05 (0.21) | | | | | 0.05 (0.22) | | | | | 0.03 (0.17) | | | | |
| Believe | 0.11 (0.33) | | | | | 0.17 (0.41) | | | | | 0.06 (0.24) | | | | |
| Trial | 0.09 (0.31) | | | | | 0.13 (0.36) | | | | | 0.57 (0.76) | | | | |
| *R^2^* |  |  |  |  |  |  |  |  |  |  |  |  |  |  |  |
| Conditional | .36 | | | | | .30 | | | | | .58 | | | | |
| Marginal | .02 | | | | | .02 | | | | | .05 | | | | |
| *Note.* CI = confidence interval. GCB = Generic Conspiracist Beliefs. Holm-corrected *p*-values are presented. | | | | | | | | | | | | | | | |

| **Table S32** | | | | | | | | | | | | | | | |
| --- | --- | --- | --- | --- | --- | --- | --- | --- | --- | --- | --- | --- | --- | --- | --- |
| *Results of Multi-Level Linear Models: Investigating Separate two-way Interactions of Spread and Believe With Participants’ Conspiracy Beliefs and Political Orientation* | | | | | | | | | | | | | | | |
|  | Conspiracy Intentions | | | | | Contact Willingness | | | | |  | | | | |
| Effect | *B*  [95% CI] | *SE* | *t* | *df* | *p* | *B*  [95% CI] | *SE* | *t* | *df* | *p* |  |  |  |  |  |
| Fixed effects |  |  |  |  |  |  |  |  |  |  |  |  |  |  |  |
| (Intercept) | 3.04  [2.59, 3.39] | 0.25 | 12.70 | 119.65 | < .001 | 4.74  [4.30, 5.06] | 0.21 | 22.08 | 197.24 | < .001 |  |  |  |  |  |
| Spread | 0.56  [0.21, 1.02] | 0.21 | 2.63 | 418.48 | .088 | -0.58  [-0.94, -0.14] | 0.20 | -2.99 | 423.81 | .029 |  |  |  |  |  |
| Believe | 1.03  [0.59, 1.40] | 0.21 | 4.79 | 412.45 | < .001 | -1.08  [-1.47, -0.69] | 0.20 | -5.41 | 406.24 | < .001 |  |  |  |  |  |
| GCB | 0.06  [-0.02, 0.15] | 0.04 | 1.37 | 410.96 | > .999 | -0.06  [-0.14, 0.02] | 0.04 | -1.52 | 410.90 | .774 |  |  |  |  |  |
| Political orientation | 0.00  [-0.03, 0.05] | 0.02 | 0.22 | 410.96 | > .999 | -0.01  [-0.04, 0.03] | 0.02 | -0.45 | 410.90 | > .999 |  |  |  |  |  |
| Political orientation^2 | -3.91  [-32.32, 27.02] | 17.10 | -0.23 | 410.96 | > .999 | -3.25  [-36.01, 24.27] | 15.69 | -0.21 | 410.90 | > .999 |  |  |  |  |  |
| GCB * Spread | -0.05  [-0.15, 0.03] | 0.05 | -1.08 | 410.96 | > .999 | 0.05  [-0.05, 0.13] | 0.05 | 1.05 | 410.90 | > .999 |  |  |  |  |  |
| GCB * Believe | -0.06  [-0.15, 0.02] | 0.05 | -1.17 | 410.96 | > .999 | 0.07  [-0.00, 0.18] | 0.05 | 1.64 | 410.90 | .708 |  |  |  |  |  |
| Political orientation  * Spread | 0.00  [-0.03, 0.04] | 0.02 | 0.13 | 410.96 | > .999 | 0.01  [-0.04, 0.05] | 0.02 | 0.27 | 410.90 | > .999 |  |  |  |  |  |
| Political orientation  * Believe | -0.06  [-0.10, 0.00] | 0.02 | -2.36 | 410.96 | .167 | 0.05  [0.01, 0.08] | 0.02 | 2.21 | 410.90 | .251 |  |  |  |  |  |
| Political orientation^2 * Spread | 12.55  [-25.68, 41.10] | 18.79 | 0.67 | 410.96 | > .999 | -16.74  [-41.07, 17.85] | 17.23 | -0.97 | 410.90 | > .999 |  |  |  |  |  |
| Political orientation^2 * Believe | -17.37  [-44.57, 11.63] | 18.53 | -0.94 | 410.96 | > .999 | 32.65  [-3.63, 58.52] | 16.99 | 1.92 | 410.90 | .443 |  |  |  |  |  |
| Random Effects | Variance (*SD*) | | | | | Variance (*SD*) | | | | |  | | | | |
| Participants intercept | 0.43 (0.66) | | | | | 0.29 (0.53) | | | | |  | | | | |
| Spread | 0.02 (0.15) | | | | | 0.03 (0.17) | | | | |  | | | | |
| Believe | 0.05 (0.22) | | | | | 0.08 (0.28) | | | | |  | | | | |
| Trial | 0.39 (0.62) | | | | | 0.34 (0.58) | | | | |  | | | | |
| *R^2^* |  |  |  |  |  |  |  |  |  |  |  |  |  |  |  |
| Conditional | .41 | | | | | .50 | | | | |  | | | | |
| Marginal | .05 | | | | | .07 | | | | |  | | | | |
| *Note.* CI = confidence interval. GCB = Generic Conspiracist Beliefs. Holm-corrected *p*-values are presented. | | | | | | | | | | | | | | | |

| **Table S33** | | | | | | | | | | | | | | | |
| --- | --- | --- | --- | --- | --- | --- | --- | --- | --- | --- | --- | --- | --- | --- | --- |
| *Results of Multi-Level Linear Models: Investigating Separate three-way Interactions of Spread and Believe With Participants’ Conspiracy Beliefs and Political Orientation* | | | | | | | | | | | | | | | |
|  | Competence | | | | | Morality | | | | | Warmth | | | | |
| Effect | *B*  [95% CI] | *SE* | *t* | *df* | *p* | *B*  [95% CI] | *SE* | *t* | *df* | *p* | *B*  [95% CI] | *SE* | *t* | *df* | *p* |
| Fixed effects |  |  |  |  |  |  |  |  |  |  |  |  |  |  |  |
| (Intercept) | 4.27  [3.67, 4.78] | 0.29 | 14.55 | 68.66 | < .001 | 4.49  [4.00, 4.87] | 0.25 | 17.70 | 191.30 | < .001 | 4.13  [3.47, 4.68] | 0.31 | 13.35 | 132.02 | < .001 |
| Spread | -0.42  [-0.84, 0.00] | 0.26 | -1.63 | 420.65 | > .999 | -0.52  [-1.08, 0.05] | 0.27 | -1.89 | 427.43 | .772 | -0.50  [-1.11, 0.03] | 0.32 | -1.59 | 423.53 | > .999 |
| Believe | -0.97  [-1.40, -0.45] | 0.27 | -3.59 | 421.21 | .006 | -1.08  [-1.72, -0.60] | 0.28 | -3.84 | 428.97 | .002 | -1.08  [-1.63, -0.56] | 0.32 | -3.34 | 427.85 | .014 |
| GCB | -0.06  [-0.15, 0.01] | 0.04 | -1.48 | 410.65 | > .999 | -0.07  [-0.16, 0.02] | 0.05 | -1.53 | 410.69 | > .999 | -0.06  [-0.15, 0.05] | 0.05 | -1.08 | 410.76 | > .999 |
| Political orientation | 0.00  [-0.04, 0.04] | 0.02 | 0.06 | 410.65 | > .999 | -0.00  [-0.05, 0.04] | 0.02 | -0.06 | 410.69 | > .999 | -0.00  [-0.05, 0.05] | 0.03 | -0.04 | 410.76 | > .999 |
| Political orientation^2 | -7.41  [-47.28, 27.17] | 16.92 | -0.44 | 410.65 | > .999 | -8.92  [-45.94, 21.36] | 17.76 | -0.50 | 410.69 | > .999 | -14.98  [-57.70, 20.68] | 20.52 | -0.73 | 410.76 | > .999 |
| GCB * Spread | 0.06  [-0.02, 0.17] | 0.06 | 1.01 | 410.65 | > .999 | 0.06  [-0.06, 0.19] | 0.06 | 0.96 | 410.69 | > .999 | 0.07  [-0.06, 0.17] | 0.07 | 0.93 | 410.76 | > .999 |
| GCB * Believe | 0.11  [-0.02, 0.22] | 0.06 | 1.76 | 410.65 | > .999 | 0.10  [-0.00, 0.22] | 0.06 | 1.61 | 410.69 | > .999 | 0.11  [-0.04, 0.23] | 0.07 | 1.45 | 410.76 | > .999 |
| Political orientation  * Spread | -0.01  [-0.06, 0.03] | 0.03 | -0.27 | 410.65 | > .999 | -0.00  [-0.07, 0.06] | 0.03 | -0.16 | 410.69 | > .999 | -0.01  [-0.07, 0.04] | 0.03 | -0.32 | 410.76 | > .999 |
| Political orientation  * Believe | 0.03  [-0.03, 0.08] | 0.03 | 0.98 | 410.65 | > .999 | 0.05  [-0.02, 0.11] | 0.03 | 1.46 | 410.69 | > .999 | 0.04  [-0.03, 0.11] | 0.04 | 1.16 | 410.76 | > .999 |
| Political orientation^2 * Spread | 2.93  [-35.90, 45.99] | 21.61 | 0.14 | 410.65 | > .999 | -5.11  [-50.44, 35.69] | 22.69 | -0.23 | 410.69 | > .999 | 5.29  [-36.85, 55.03] | 26.22 | 0.20 | 410.76 | > .999 |
| Political orientation^2 * Believe | 40.27  [-0.20, 91.19] | 22.85 | 1.76 | 410.65 | > .999 | 51.19  [-2.97, 105.69] | 23.99 | 2.13 | 410.69 | .468 | 63.54  [19.26, 121.67] | 27.72 | 2.29 | 410.76 | .313 |
| Spread * Believe | 0.05  [-0.56, 0.74] | 0.35 | 0.16 | 410.65 | > .999 | 0.12  [-0.64, 0.89] | 0.36 | 0.32 | 410.69 | > .999 | 0.10  [-0.58, 0.84] | 0.42 | 0.25 | 410.76 | > .999 |
| GCB * Spread * Believe | -0.03  [-0.18, 0.14] | 0.08 | -0.41 | 410.65 | > .999 | -0.07  [-0.23, 0.09] | 0.09 | -0.82 | 410.69 | > .999 | -0.09  [-0.25, 0.09] | 0.10 | -0.86 | 410.76 | > .999 |
| Political orientation * Spread * Believe | 0.00  [-0.07, 0.08] | 0.04 | 0.06 | 410.65 | > .999 | 0.02  [-0.07, 0.10] | 0.04 | 0.41 | 410.69 | > .999 | 0.02  [-0.06, 0.12] | 0.05 | 0.46 | 410.76 | > .999 |
| Political orientation^2 * Spread * Believe | -44.88  [-103.32, 4.83] | 31.14 | -1.44 | 410.65 | > .999 | -30.65  [-93.75, 41.27] | 32.70 | -0.94 | 410.69 | > .999 | -44.90  [-108.26, 28.13] | 37.78 | -1.19 | 410.76 | > .999 |
| Random Effects | Variance (*SD*) | | | | | Variance (*SD*) | | | | | Variance (*SD*) | | | | |
| Participants intercept | 0.90 (0.95) | | | | | 0.44 (0.67) | | | | | 0.74 (0.86) | | | | |
| Spread | 0.03 (0.17) | | | | | 0.06 (0.24) | | | | | 0.05 (0.21) | | | | |
| Believe | 0.08 (0.29) | | | | | 0.08 (0.28) | | | | | 0.08 (0.28) | | | | |
| Trial | 0.26 (0.51) | | | | | 0.30 (0.55) | | | | | 0.42 (0.65) | | | | |
| *R^2^* |  |  |  |  |  |  |  |  |  |  |  |  |  |  |  |
| Conditional | .53 | | | | | .49 | | | | | .65 | | | | |
| Marginal | .04 | | | | | .06 | | | | | .05 | | | | |
| *Note.* CI = confidence interval. GCB = Generic Conspiracist Beliefs. Holm-corrected *p*-values are presented. | | | | | | | | | | | | | | | |

| **Table S34** | | | | | | | | | | | | | | | |
| --- | --- | --- | --- | --- | --- | --- | --- | --- | --- | --- | --- | --- | --- | --- | --- |
| *Results of Multi-Level Linear Models: Investigating Separate three-way Interactions of Spread and Believe With Participants’ Conspiracy Beliefs and Political Orientation* | | | | | | | | | | | | | | | |
|  | Machiavellianism | | | | | Narcissism | | | | | Psychopathy | | | | |
| Effect | *B*  [95% CI] | *SE* | *t* | *df* | *p* | *B*  [95% CI] | *SE* | *t* | *df* | *p* | *B*  [95% CI] | *SE* | *t* | *df* | *p* |
| Fixed effects |  |  |  |  |  |  |  |  |  |  |  |  |  |  |  |
| (Intercept) | 3.50  [3.14, 3.91] | 0.22 | 16.14 | 57.02 | < .001 | 3.34  [2.92, 3.80] | 0.21 | 15.95 | 119.88 | < .001 | 2.54  [1.87, 3.19] | 0.37 | 6.87 | 116.98 | < .001 |
| Spread | 0.34  [-0.04, 0.74] | 0.19 | 1.83 | 407.50 | .883 | 0.27  [-0.10, 0.60] | 0.21 | 1.25 | 415.80 | > .999 | 0.88  [0.25, 1.60] | 0.37 | 2.39 | 417.60 | .227 |
| Believe | 0.55  [0.13, 0.84] | 0.20 | 2.77 | 336.37 | .088 | 0.74*  [0.42, 1.08] | 0.23 | 3.21 | 329.43 | .022 | 1.34  [0.71, 1.87] | 0.38 | 3.54 | 421.90 | .007 |
| GCB | 0.02  [-0.04, 0.08] | 0.03 | 0.79 | 410.35 | > .999 | 0.05  [-0.03, 0.11] | 0.04 | 1.44 | 410.45 | > .999 | 0.08  [-0.03, 0.22] | 0.06 | 1.23 | 410.90 | > .999 |
| Political orientation | 0.00  [-0.03, 0.03] | 0.02 | 0.04 | 410.35 | > .999 | -0.02  [-0.04, 0.02] | 0.02 | -0.83 | 410.45 | > .999 | 0.03  [-0.04, 0.08] | 0.03 | 0.88 | 410.90 | > .999 |
| Political orientation^2 | -0.70  [-25.15, 23.84] | 11.83 | -0.06 | 410.35 | > .999 | 27.88  [2.35, 48.38] | 13.79 | 2.02 | 410.45 | .527 | 25.64  [-19.97, 57.30] | 24.22 | 1.06 | 410.90 | > .999 |
| GCB * Spread | -0.03  [-0.11, 0.06] | 0.04 | -0.77 | 410.35 | > .999 | -0.05  [-0.12, 0.04] | 0.05 | -0.96 | 410.45 | > .999 | -0.09  [-0.27, 0.04] | 0.08 | -1.09 | 410.90 | > .999 |
| GCB * Believe | -0.06  [-0.14, 0.03] | 0.04 | -1.30 | 410.35 | > .999 | -0.10  [-0.19, 0.01] | 0.05 | -1.97 | 410.45 | .541 | -0.10  [-0.28, 0.04] | 0.09 | -1.12 | 410.90 | > .999 |
| Political orientation  * Spread | -0.00  [-0.04, 0.03] | 0.02 | -0.24 | 410.35 | > .999 | 0.01  [-0.03, 0.05] | 0.02 | 0.57 | 410.45 | > .999 | -0.02  [-0.09, 0.06] | 0.04 | -0.49 | 410.90 | > .999 |
| Political orientation  * Believe | -0.02  [-0.06, 0.02] | 0.02 | -0.89 | 410.35 | > .999 | -0.02  [-0.07, 0.03] | 0.02 | -0.80 | 410.45 | > .999 | -0.07  [-0.16, 0.01] | 0.04 | -1.68 | 410.90 | > .999 |
| Political orientation^2 * Spread | 3.56  [-30.17, 38.75] | 15.11 | 0.24 | 410.35 | > .999 | -33.81  [-66.83, -4.18] | 17.62 | -1.92 | 410.45 | .556 | -23.30  [-68.39, 31.05] | 30.94 | -0.75 | 410.90 | > .999 |
| Political orientation^2 * Believe | -29.85  [-63.06, 1.51] | 15.97 | -1.87 | 410.35 | .872 | -49.41  [-84.17, -9.62] | 18.62 | -2.65 | 410.45 | .116 | -84.22  [-140.72, -26.88] | 32.71 | -2.57 | 410.90 | .145 |
| Spread * Believe | -0.09  [-0.58, 0.44] | 0.24 | -0.37 | 410.35 | > .999 | -0.10  [-0.65, 0.31] | 0.28 | -0.36 | 410.45 | > .999 | -0.34  [-1.20, 0.69] | 0.50 | -0.68 | 410.90 | > .999 |
| GCB * Spread * Believe | 0.04  [-0.07, 0.13] | 0.06 | 0.61 | 410.35 | > .999 | 0.06  [-0.03, 0.18] | 0.07 | 0.94 | 410.45 | > .999 | 0.08  [-0.11, 0.32] | 0.12 | 0.72 | 410.90 | > .999 |
| Political orientation * Spread * Believe | 0.00  [-0.06, 0.06] | 0.03 | 0.06 | 410.35 | > .999 | -0.00  [-0.07, 0.05] | 0.03 | -0.15 | 410.45 | > .999 | 0.02  [-0.12, 0.12] | 0.06 | 0.35 | 410.90 | > .999 |
| Political orientation^2 * Spread * Believe | 31.45  [-16.06, 82.10] | 21.77 | 1.44 | 410.35 | > .999 | 53.64  [2.31, 107.93] | 25.39 | 2.11 | 410.45 | .458 | 82.25  [0.12, 158.29] | 44.59 | 1.84 | 410.90 | .790 |
| Random Effects | Variance (*SD*) | | | | | Variance (*SD*) | | | | | Variance (*SD*) | | | | |
| Participants intercept | 0.54 (0.74) | | | | | 0.35 (0.59) | | | | | 1.06 (1.03) | | | | |
| Spread | 0.05 (0.21) | | | | | 0.05 (0.22) | | | | | 0.03 (0.17) | | | | |
| Believe | 0.11 (0.33) | | | | | 0.17 (0.41) | | | | | 0.06 (0.24) | | | | |
| Trial | 0.09 (0.31) | | | | | 0.13 (0.35) | | | | | 0.56 (0.75) | | | | |
| *R^2^* |  |  |  |  |  |  |  |  |  |  |  |  |  |  |  |
| Conditional | .36 | | | | | .30 | | | | | .58 | | | | |
| Marginal | .02 | | | | | .02 | | | | | .05 | | | | |
| *Note.* CI = confidence interval. GCB = Generic Conspiracist Beliefs. Holm-corrected *p*-values are presented. | | | | | | | | | | | | | | | |

| **Table S35** | | | | | | | | | | | | | | | |
| --- | --- | --- | --- | --- | --- | --- | --- | --- | --- | --- | --- | --- | --- | --- | --- |
| *Results of Multi-Level Linear Models: Investigating Separate three-way Interactions of Spread and Believe With Participants’ Conspiracy Beliefs and Political Orientation* | | | | | | | | | | | | | | | |
|  | Conspiracy Intentions | | | | | Contact Willingness | | | | |  | | | | |
| Effect | *B*  [95% CI] | *SE* | *t* | *df* | *p* | *B*  [95% CI] | *SE* | *t* | *df* | *p* |  |  |  |  |  |
| Fixed effects |  |  |  |  |  |  |  |  |  |  |  |  |  |  |  |
| (Intercept) | 2.99  [2.43,3.54] | 0.29 | 10.46 | 181.98 | < .001 | 4.83  [4.41, 5.25] | 0.25 | 19.26 | 279.73 | < .001 |  |  |  |  |  |
| Spread | 0.65  [-0.08, 1.33] | 0.31 | 2.10 | 417.93 | .511 | -0.76  [-1.26, -0.25] | 0.29 | -2.64 | 421.05 | .121 |  |  |  |  |  |
| Believe | 1.15  [0.57, 1.69] | 0.32 | 3.60 | 422.59 | .005 | -1.27  [-1.76, -0.62] | 0.30 | -4.31 | 428.24 | < .001 |  |  |  |  |  |
| GCB | 0.09  [-0.03, 0.17] | 0.05 | 1.69 | 410.95 | > .999 | -0.09  [-0.18, 0.01] | 0.05 | -1.88 | 410.90 | .671 |  |  |  |  |  |
| Political orientation | -0.00  [-0.04, 0.06] | 0.03 | -0.02 | 410.95 | > .999 | -0.01  [-0.07, 0.05] | 0.02 | -0.49 | 410.90 | > .999 |  |  |  |  |  |
| Political orientation^2 | 14.30  [-26.04, 50.12] | 20.36 | 0.70 | 410.95 | > .999 | -11.88  [-45.75, 25.25] | 18.69 | -0.64 | 410.90 | > .999 |  |  |  |  |  |
| GCB * Spread | -0.10  [-0.22, 0.04] | 0.07 | -1.45 | 410.95 | > .999 | 0.10  [-0.05, 0.02] | 0.06 | 1.54 | 410.90 | > .999 |  |  |  |  |  |
| GCB * Believe | -0.12  [-0.23, 0.04] | 0.07 | -1.65 | 410.95 | > .999 | 0.13  [0.01, 0.26] | 0.07 | 1.99 | 410.90 | .612 |  |  |  |  |  |
| Political orientation  * Spread | 0.01  [-0.06, 0.07] | 0.03 | 0.30 | 410.95 | > .999 | 0.01  [-0.06, 0.07] | 0.03 | 0.38 | 410.90 | > .999 |  |  |  |  |  |
| Political orientation  * Believe | -0.04  [-0.11, 0.01] | 0.04 | -1.25 | 410.95 | > .999 | 0.05  [-0.02, 0.12] | 0.03 | 1.59 | 410.90 | > .999 |  |  |  |  |  |
| Political orientation^2 * Spread | -14.80  [-65.71, 40.71] | 26.00 | -0.57 | 410.95 | > .999 | -5.14  [-47.45, 39.68] | 23.88 | -0.22 | 410.90 | > .999 |  |  |  |  |  |
| Political orientation^2 * Believe | -51.69  [-102.21, -10.29] | 27.49 | -1.88 | 410.95 | .790 | 49.41  [-5.37, 103.10] | 25.24 | 1.96 | 410.90 | .612 |  |  |  |  |  |
| Spread * Believe | -0.21  [-1.03, 0.61] | 0.42 | -0.50 | 410.95 | > .999 | 0.34  [-0.49, 0.90] | 0.38 | 0.89 | 410.90 | > .999 |  |  |  |  |  |
| GCB * Spread * Believe | 0.10  [-0.15, 0.28] | 0.10 | 1.03 | 410.95 | > .999 | -0.11  [-0.27, 0.05] | 0.09 | -1.16 | 410.90 | > .999 |  |  |  |  |  |
| Political orientation * Spread * Believe | -0.01  [-0.08, 0.08] | 0.05 | -0.30 | 410.95 | > .999 | -0.01  [-0.11, 0.09] | 0.04 | -0.22 | 410.90 | > .999 |  |  |  |  |  |
| Political orientation^2 * Spread * Believe | 57.44  [-14.17, 128.68] | 37.47 | 1.53 | 410.95 | > .999 | -25.66  [-82.06, 30.18] | 34.41 | -0.75 | 410.90 | > .999 |  |  |  |  |  |
| Random Effects | Variance (*SD*) | | | | | Variance (*SD*) | | | | |  | | | | |
| Participants intercept | 0.43 (0.66) | | | | | 0.29 (0.53) | | | | |  | | | | |
| Spread | 0.02 (0.15) | | | | | 0.03 (0.17) | | | | |  | | | | |
| Believe | 0.04 (0.22) | | | | | 0.08 (0.28) | | | | |  | | | | |
| Trial | 0.38 (0.62) | | | | | 0.34 (0.58) | | | | |  | | | | |
| *R^2^* |  |  |  |  |  |  |  |  |  |  |  |  |  |  |  |
| Conditional | .41 | | | | | .50 | | | | |  | | | | |
| Marginal | .06 | | | | | .07 | | | | |  | | | | |
| *Note.* CI = confidence interval. GCB = Generic Conspiracist Beliefs. Holm-corrected *p*-values are presented. | | | | | | | | | | | | | | | |

***Section 3: Ratings of Aggregated Classification Images (Condition)***
 The aggregated images from each condition (see Figure 9 in the manuscript) were analyzed using separate repeated-measures ANOVAs for each rating dimension to test the effect of condition on the ratings. Holm-adjusted *p-*values are reported for multiple comparisons. The ANOVA results are displayed in Table S33 below.

| **Table S36** | | | | | | | | |
| --- | --- | --- | --- | --- | --- | --- | --- | --- |
| *Effects of Condition on Image Ratings in Repeated-Measures ANOVAs* | | | | | | | | |
|  | | | | | Condition | | | |
|  | | | | | Spread = No  Believe = No | Spread = No  Believe = Yes | Spread = Yes  Believe = No | Spread = Yes  Believe = Yes |
| Variable | *F* | *p* | η_p_^2^ | *N* | *M (SD)* | *M (SD)* | *M (SD)* | *M (SD)* |
| Competence | 23.88 | < .001 | .56 | 20 | 4.20 (1.85) | 3.80 (1.51) | 4.95 (1.57) | 1.60 (1.27) |
| Morality | 54.03 | < .001 | .71 | 23 | 5.13 (1.55) | 3.57 (0.99) | 5.00 (1.13) | 1.61 (0.89) |
| Warmth | 51.45 | <. 001 | .72 | 21 | 5.75 (1.14) | 3.33 (1.12) | 4.62 (1.36) | 1.76 (1.45) |
| Machiavellianism | 6.94 | .002 | .27 | 20 | 3.30 (1.84) | 3.55 (0.95) | 3.50 (1.40) | 5.10 (1.89) |
| Narcissism | 5.22 | .007 | .21 | 21 | 2.52 (1.69) | 3.43 (1.33) | 3.33 (1.35) | 4.67 (2.35) |
| Psychopathy | 25.50 | < .001 | .57 | 20 | 2.60 (1.96) | 4.05 (1.79) | 2.40 (1.54) | 6.40 (1.31) |
| Conspiracy Intentions | 33.89 | < .001 | .65 | 19 | 2.42 (1.64) | 3.89 (1.52) | 2.37 (0.90) | 5.95 (1.13) |
| Contact Willingness | 40.19 | < .001 | .66 | 22 | 5.50 (1.10) | 3.55 (0.86) | 5.14 (1.08) | 2.09 (1.72) |
| Gender Resemblance | 11.68 | < .001 | .42 | 17 | 7.65 (2.15) | 5.06 (2.75) | 6.24 (2.80) | 2.94 (2.56) |
|  | | | | | | | | |

**Competence.** The individual who neither believe nor spread conspiracy theories was perceived as significantly more competent than the individual who only spread (*d* = 1.58, *p_Holm_* < .001), but did not differ significantly from the individual who only believe (*d* = 0.74, *p_Holm_* = .060), or the individual who both believe and spread (*d* = 0.24, *p_Holm_* = .397). The individual who only believe was also perceived as significantly more competent than the individual who only spread (*d* = 2.35, *p_Holm_* < .001), but not significantly different from the individual who both spread and believe (*d* = 0.44, *p_Holm_* = .122). Lastly, the individual who only spread was seen as significantly less competent than the individual who both spread and believe (*d* = 1.64, *p_Holm_* < .001).  **Morality.** The individual who neither spread nor believe conspiracy theories was perceived as significantly more moral than the individual who only believe (*d* = 1.20, *p_Holm_* < .001), and the individual who both spread and believe (*d* = 2.79, *p_Holm_* < .001). There was no significant difference between the individual who both spread and believe and the individual who only spread (*d* = 0.10, *p_Holm_* = .695). The individual who only believe was perceived as significantly less competent than the individual who only spread (*d* = 1.35, *p_Holm_* < .001) , but more competent than the individual who both spread and believe (*d* = 2.08, *p_Holm_* < .001). Lastly, the individual who only spread was perceived as significantly more competent than the individual who both spread and believe (*d* = 3.33, *p_Holm_* < .001).
 **Warmth.** The individual who neither spread nor believe conspiracy theories was perceived as significantly warmer than the individual who only believe (*d* = 2.14, *p_Holm_* < .001), the individual who only spread (*d* = 0.90, *p_Holm_* = .003), and the individual who both spread and believe (*d* = 3.06, *p_Holm_* < .001). The individual who only believe was perceived as significantly less warm than the individual who only spread (*d* = 1.04, *p_Holm_* < .001), but warmer than the individual who both spread and believe (*d* = 1.21, *p_Holm_* < .001). Lastly, the individual who only spread was perceived as significantly warmer than the individual who both spread and believe (*d* = 2.03, *p_Holm_* < .001).
 **Machiavellianism.** The individual who neither spread nor believe conspiracy theories was perceived as significantly less Machiavellian than the individual who both spread and believe (*d* = 0.97, *p_Holm_* =.028). There was no significant difference between the individual who neither spread nor believe and the individual who only believe (*d* = 0.17, *p_Holm_* > .999), or the individual who only spread (*d* = 0.12, *p_Holm_* > .999). There was neither a significant difference between the individual who only spread and the individual who only believe (*d* = 0.04, *p_Holm_* > .999). However, the individual who both spread and believe was perceived as significantly more Machiavellian than the individual who only believe (*d* = 1.04, *p_Holm_* = .018), and the individual who only spread (*d* = 0.96, *p_Holm_* = .020). **Narcissism.** The individual who neither spread nor believe in conspiracy theories was perceived as significantly less narcissistic than the individual who both spread and believe (*d* = 1.05, *p_Holm_* = .024). The individual who neither spread nor believe did not significantly differ from the individual who only believe (*d* = 0.60, *p_Holm_* = .250), nor did it differ from the individual who only spread (*d* = 0.53, *p_Holm_* = .075). There was also no significant difference between the individual who only spread and the individual who only believe (*d* = 0.07, *p_Holm_* = .859), nor between the individual who only believe and the individual who both spread and believe (*d* = 0.64, *p_Holm_* = .159). Lastly, there was no significant difference between the individual who only spread and the individual who both spread and believe (*d* = 0.70, *p_Holm_* = .112).
 **Psychopathy.** The individual who neither spread nor believe in conspiracy theories was perceived as significantly less psychopathic than the individual who both spread and believe (*d* = 2.28, *p_Holm_* < .001). There was no significant difference between the individual who neither spread and believe and the individual who only believe (*d* = 0.77, *p_Holm_* = .062), nor with the individual who only spread (*d* = 0.11, *p_Holm_* = .654). The individual who only believe was perceived as significantly more psychopathic than the individual who only spread (*d* = 0.99, *p_Holm_* < .001), and significantly less psychopathic than the individual who both spread and believe (*d* = 1.50, *p_Holm_* < .001). Lastly, the individual who only spread was perceived as significantly less psychopathic than the individual who both spread and believe (*d* = 2.80, *p_Holm_* < .001). **Conspiracy Intentions.** The individual who neither spread nor believe conspiracy theories was perceived as having lower intentions to conspire than the individual who only believe (*d* = 0.93, *p_Holm_* = .004), and the individual who both spread and believe (*d* = 2.50, *p_Holm_* < .001). There was no significant difference between the individual who neither spread nor believe and the individual who only spread (*d* = 0.04, *p_Holm_* = .884). The individual who only believe was perceived as having significantly higher intentions to conspire than the individual who only spread (*d* = 1.22, *p_Holm_* < .001), but significantly lower intentions to conspire than the individual who both spread and believe (*d* = 1.54, *p_Holm_* < .001). Lastly, the individual who only spread was perceived as having significantly lower intentions to conspire than the individual who both spread and believe (*d* = 3.50, *p_Holm_* < .001). **Contact Willingness.** The participants indicate a significant higher willingness to have contact with the individual who neither spread nor believe conspiracy theories than the individual who only believe (*d* =1.98, *p_Holm_* < .001), and the individual that both spread and believe (*d* = 2.36, *p_Holm_* < .001). There was no significant difference between the individual who neither spread nor believe and the individual who only spread (*d* = 0.33, *p_Holm_* = .134). Participant also indicated a significant lower willingness to have contact with the individual who only believe than the individual who only spread (*d* = 1.63, *p_Holm_* < .001), but a higher willingness to have contact with the individual who only believe than the individual who both spread and believe (*d* = 1.07, *p_Holm_* < .001). Lastly, participants were significantly more positive to have contact with the individual who only spread than the individual who both spread and believe (*d* = 2.12, *p_Holm_* < .001). **Gender Resemblance*.*** The individual who neither spread nor believe conspiracy theories was perceived as significantly more female than the individual who only believe (*d* = 1.05, *p_Holm_* = .032), and significantly more female than the individual who both spread and believe (*d* = 1.98, *p_Holm_* < .001). There was no significant difference in gender perception of the individual who neither spread nor believe and the individual who only spread (*d* = 0.56, *p_Holm_* = .204). The individual who only believe was perceived as significantly less male than the individual who both spread and believe (*d* = 0.79, *p_Holm_* = .036). There was no significant difference in gender perception between the individual who only believe and the individual who only spread (*d* = 0.43, *p_Holm_* = .204). Lastly, the individual who only spread was perceived as significantly less male (more female) than the individual who both spread and believe (*d* = 1.22, *p_Holm_* = .015). ***Section 3 cont.: Ratings of Aggregated Classification Images (Conspiracy Beliefs)*** For each of our rating dimensions, we ran separate 2 (conspiracy beliefs: low vs. high) x 4 (condition: neither believe nor spread, believe but do not spread, spread but do not believe, both believe and spread) repeated-measures ANOVAs to test whether the classification images were moderated by participants own conspiracy beliefs.
 **Competence.** Results revealed significant main effects of condition, *F*(1.95, 37.02) = 26.55, η_p_^2^ = .58, *p* < .001, and conspiracy beliefs, *F*(1, 19) = 12.76, η_p_^2^ = .40, *p* = .002, and the condition x conspiracy beliefs interaction effect, *F*(3, 57) = 3.43, η_p_^2^ = .15, *p* = .023.
 Simple effects analysis revealed a significant effect of conspiracy beliefs on perceived competence of the classification image of the individual who believe but do not spread conspiracy theories, *F*(1, 19) = 15.83, η_p_^2^ = .46, *p* < .001. Pairwise comparisons revealed that the classification image of the individual who believe in conspiracy theories, generated from participants high in conspiracy beliefs, was perceived as significantly more competent than the classification image generated from those low in conspiracy beliefs (*d* = 0.76, *p* < .001). The moderating effect of participants’ conspiracy beliefs was not significant in any of the other conditions (all *ps.* > .05).
 **Morality.** Results revealed a significant main effect of condition, *F*(1.78, 39.25) = 50.68, η_p_^2^ = .70, *p* < .001, and a significant condition x conspiracy beliefs interaction effect, *F*(2.18, 47.93) = 3.88, η_p_^2^ = .15, *p* = .024. The main effect of conspiracy beliefs was not significant, *F*(1, 22) = 2.63, η_p_^2^ = .11, *p* = .119.
 Simple effects analysis revealed a significant effect of conspiracy beliefs on perceived morality of the classification image of the individual who believe but do not spread conspiracy theories, *F*(1, 22) = 14.14, η_p_^2^ = .39, *p* < .001. Pairwise comparisons revealed that the classification image of the individual who believe in conspiracy theories, generated from participants high in conspiracy beliefs, was perceived as significantly more moral than the classification image generated from those low in conspiracy beliefs (*d* = 0.77, *p* < .001). The moderating effect of participants’ conspiracy beliefs was not significant in any of the other conditions (all *ps.* > .05).
 **Warmth.** Results revealed significant main effects of condition, *F*(1.91, 38.20) = 79.01, η_p_^2^ = .80, *p* < .001, and conspiracy beliefs, *F*(1, 20) = 6.50, η_p_^2^ = .25, *p* = .019, and the condition x conspiracy beliefs interaction effect, *F*(3, 60) = 3.29, η_p_^2^ = .14, *p* = .027.
 Simple effects analysis revealed a significant effect of conspiracy beliefs on perceived warmth of the classification image of the individual who believe but do not spread conspiracy theories, *F*(1, 20) = 14.14, η_p_^2^ = .25, *p* = .018. Pairwise comparisons revealed that the classification image of the individual who believe in conspiracy theories, generated from participants high in conspiracy beliefs, was perceived as significantly warmer than the classification image generated from those low in conspiracy beliefs (*d* = 0.52, *p* = .018). The moderating effect of participants’ conspiracy beliefs was not significant in any of the other conditions (all *ps.* > .05).
 **Narcissism.** Results revealed a significant main effect of condition, *F*(2.03, 40.50) = 11.81, η_p_^2^ = .37, *p* < .001. The main effect of conspiracy beliefs was not significant, *F*(1, 20) = 0.81, η_p_^2^ = .04, *p* = .380, neither was the condition x conspiracy beliefs interaction effect, *F*(3, 60) = 1.70, η_p_^2^ = .08, *p* = .078.
 Simple effects analysis revealed no significant effects of conspiracy beliefs within each condition (all *ps.* > .05).
 **Machiavellianism.** Results revealed a significant main effect of condition, *F*(1.80, 34.26) = 10.55, η_p_^2^ = .36, *p* < .001. The main effect of conspiracy beliefs was not significant, *F*(1, 19) = 0.37, η_p_^2^ = .02, *p* = .550, neither was the condition x conspiracy beliefs interaction effect, *F*(3, 57) = 0.41, η_p_^2^ = .02, *p* = .744.
 Simple effects analysis revealed no significant effects of conspiracy beliefs within each condition (all *ps.* > .05).
 **Psychopathy.** Results revealed significant main effects of condition, *F*(3, 57) = 35.83, η_p_^2^ = .65, *p* < .001, and conspiracy beliefs, *F*(1, 19) = 6.00, η_p_^2^ = .24, *p* = .024, and the condition x conspiracy beliefs interaction effect, *F*(3, 57) = 2.88, η_p_^2^ = .13, *p* = .044.
 Simple effects analysis revealed a significant effect of conspiracy beliefs on perceived psychopathy of the classification image of the individual who believe but do not spread conspiracy theories, *F*(1, 19) = 9.75, η_p_^2^ = .34, *p* = .006. Pairwise comparisons revealed that the classification image of the individual who believe in conspiracy theories, generated from participants high in conspiracy beliefs, was perceived as significantly less psychopathic than the classification image generated from those low in conspiracy beliefs (*d* = 0.61, *p* = .006). The moderating effect of participants’ conspiracy beliefs was not significant in any of the other conditions (all *ps.* > .05).
 **Conspiracy Intentions.** Results revealed significant main effects of condition, *F*(1.75, 31.43) = 36.50, η_p_^2^ = .67, *p* < .001, and conspiracy beliefs, *F*(1, 18) = 4.86, η_p_^2^ = .21, *p* = .041, and the condition x conspiracy beliefs interaction effect, *F*(3, 54) = 6.25, η_p_^2^ = .26, *p* = .001.
 Simple effects analysis revealed a significant effect of conspiracy beliefs on perceived conspiracy intentions of the classification image of the individual who believe but do not spread conspiracy theories, *F*(1, 18) = 17.58, η_p_^2^ = .49, *p* < .001. Pairwise comparisons revealed that the classification image of the individual who believe in conspiracy theories, generated from participants high in conspiracy beliefs, was perceived as having significantly lower intentions to conspire than the classification image generated from those low in conspiracy beliefs (*d* = 1.10, *p* < .001). The moderating effect of participants’ conspiracy beliefs was not significant in any of the other conditions (all *ps.* > .05).
 **Contact Willingness.** Results revealed significant main effects of condition, *F*(1.52, 31.90) = 60.34, η_p_^2^ = .74, *p* < .001, and conspiracy beliefs, *F*(1, 21) = 12.00, η_p_^2^ = .36, *p* = .002. The interaction effect of condition x conspiracy beliefs was not significant, *F*(3, 63) = 1.97, η_p_^2^ = .09, *p* = .127.
 Simple effects analysis revealed a significant effect of conspiracy beliefs on contact willingness with the individual who believe but do not spread conspiracy theories, *F*(1, 21) = 8.09, η_p_^2^ = .28, *p* = .010. Pairwise comparisons revealed that the classification image of the individual who believe in conspiracy theories, generated from participants high in conspiracy beliefs, was rated significantly more positive in terms of contact willingness than the classification image generated from those low in conspiracy beliefs (*d* = 0.83, *p* = .010).
 There was also a significant effect of conspiracy beliefs on contact willingness with the individual who spread but do not believe in conspiracy theories, *F*(1, 21) = 5.91, η_p_^2^ = .22, *p* = .024. Pairwise comparisons revealed that the classification image of the individual who spread conspiracy theories, generated from participants high in conspiracy beliefs, was rated significantly more positive in terms of contact willingness than the classification image generated from those low in conspiracy beliefs (*d* = 0.51, *p* = .024).
***Section 3 cont.: Ratings of Aggregated Classification Images (Political Orientation)*** For each of our rating dimensions, we ran separate 2 (political orientation: left vs. right) x 4 (condition: neither believe nor spread, believe but do not spread, spread but do not believe, both believe and spread) repeated-measures ANOVAs to test whether the classification images were moderated by participants own political orientation.
 **Competence.** Results revealed significant main effects of condition, *F*(1.75, 33.13) = 33.47, η_p_^2^ = .64, *p* < .001, and the condition x political orientation interaction effect, *F*(3, 57) = 5.91, η_p_^2^ = .24, *p* = .001. The main effect of political orientation was not significant, *F*(1, 19) = 0.01, η_p_^2^ = .00, *p* = .922.
 Simple effects analysis revealed a significant effect of political orientation on perceived competence of the classification image of the individual who believe but do not spread conspiracy theories, *F*(1, 19) = 9.32, η_p_^2^ = .33, *p* = .007. Pairwise comparisons revealed that the classification image of the individual who believe in conspiracy theories, generated from participants on the political right, was perceived as significantly more competent than the classification image generated from those on the political left (*d* = 0.85, *p* = .007). The moderating effect of participants’ political orientation was not significant in any of the other conditions (all *ps.* > .05).
 **Morality.** Results revealed significant main effects of condition, *F*(1.91, 42.12) = 36.70, η_p_^2^ = .63, *p* < .001, political orientation, *F*(1, 22) = 19.90, η_p_^2^ = .48, *p* < .001, and the condition x political orientation interaction effect, *F*(2.27, 49.95) = 10.36, η_p_^2^ = .32, *p* < .001. Simple effects analysis revealed a significant effect of political orientation on perceived morality of the classification image of the individual who believe but do not spread conspiracy theories, *F*(1, 22) = 37.18, η_p_^2^ = .63, *p* < .001. Pairwise comparisons revealed that the classification image of the individual who believe in conspiracy theories, generated from participants on the political right, was perceived as significantly more moral than the classification image generated from those on the political left (*d* = 1.35, *p* < .001). The moderating effect of participants’ political orientation was not significant in any of the other conditions (all *ps.* > .05).
 **Warmth.** Results revealed significant main effects of condition, *F*(3, 60) = 115.57, η_p_^2^ = .85, *p* < .001, political orientation, *F*(1, 20) = 47.24, η_p_^2^ = .70, *p* < .001, and the condition x political orientation interaction effect, *F*(3, 60) = 21.03, η_p_^2^ = .51, *p* < .001.
 Simple effects analysis revealed a significant effect of political orientation on perceived warmth of the classification image of the individual who believe but do not spread conspiracy theories, *F*(1, 20) = 52.26, η_p_^2^ = .72, *p* < .001. Pairwise comparisons revealed that the classification image of the individual who believe in conspiracy theories, generated from participants on the political right, was perceived as significantly warmer than the classification image generated from those on the political left (*d* = 2.17, *p* < .001). The moderating effect of participants’ political orientation was not significant in any of the other conditions (all *ps.* > .05).
 **Narcissism.** Results revealed a significant main effect of condition, *F*(1.47, 29.32) = 11.19, η_p_^2^ = .36, *p* < .001, and a significant condition x political orientation interaction effect, *F*(3, 60) = 3.65, η_p_^2^ = .15, *p* = .017. The main effect of political orientation was not significant, *F*(1, 20) = 4.31, η_p_^2^ = .18, *p* = .051.
 Simple effects analysis revealed a significant effect of political orientation on perceived narcissism of the classification image of the individual who believe but do not spread conspiracy theories, *F*(1, 20) = 10.46, η_p_^2^ = .34, *p* = .004. Pairwise comparisons revealed that the classification image of the individual who believe in conspiracy theories, generated from participants on the political right, was perceived as significantly less narcissistic than the classification image generated from those on the political left (*d* = 1.12, *p* < .001). The moderating effect of participants’ political orientation was not significant in any of the other conditions (all *ps.* > .05).
 **Machiavellianism.** Results revealed a significant main effect of condition, *F*(2.15, 40.76) = 15.33, η_p_^2^ = .45, *p* < .001, and a significant condition x political orientation interaction effect, *F*(3, 57) = 4.34, η_p_^2^ = .19, *p* = .008. The main effect of political orientation was not significant, *F*(1, 19) = 0.68, η_p_^2^ = .03, *p* = .420.
 Simple effects analysis revealed a significant effect of political orientation on perceived Machiavellianism of the classification image of the individual who believe but do not spread conspiracy theories, *F*(1, 19) = 6.08, η_p_^2^ = .24, *p* = .023. Pairwise comparisons revealed that the classification image of the individual who believe in conspiracy theories, generated from participants on the political right, was perceived as significantly less Machiavellian than the classification image generated from those on the political left (*d* = 0.68, *p* = .023). The moderating effect of participants’ political orientation was not significant in any of the other conditions (all *ps.* > .05).
 **Psychopathy.** Results revealed significant main effects of condition, *F*(2.19, 41.61) = 55.19, η_p_^2^ = .74, *p* < .001, political orientation, *F*(1, 19) = 25.72, η_p_^2^ = .58, *p* < .001, and the condition x political orientation interaction effect, *F*(3, 57) = 19.58, η_p_^2^ = .51, *p* < .001.
 Simple effects analysis revealed a significant effect of political orientation on perceived manipulativeness of the classification image of the individual who believe but do not spread conspiracy theories, *F*(1, 19) = 49.76, η_p_^2^ = .72, *p* < .001. Pairwise comparisons revealed that the classification image of the individual who believe in conspiracy theories, generated from participants on the political right, was perceived as significantly less psychopathic than the classification image generated from those on the political left (*d* = 2.05, *p* < .001). The moderating effect of participants’ political orientation was not significant in any of the other conditions (all *ps.* > .05).
 **Conspiracy Intentions.** Results revealed a significant main effect of condition, *F*(3, 54) = 77.28, η_p_^2^ = .81, *p* < .001, and a significant condition x political orientation interaction effect, *F*(3, 54) = 6.08, η_p_^2^ = .25, *p* = .001. The main effect of political orientation was not significant, *F*(1, 18) = 1.47, η_p_^2^ = .08, *p* = .239.
 Simple effects analysis revealed a significant effect of political orientation on perceived conspiracy intentions of the classification image of the individual who believe but do not spread conspiracy theories, *F*(1, 18) = 7.71, η_p_^2^ = .30, *p* = .012. Pairwise comparisons revealed that the classification image of the individual who believe in conspiracy theories, generated from participants on the political right, was perceived as having significantly lower intentions to conspire than the classification image generated from those on the political left (*d* = 0.94, *p* = .012). The moderating effect of participants’ political orientation was not significant in any of the other conditions (all *ps.* > .05).
 **Contact Willingness.** Results revealed a significant main effect of condition, *F*(2.00, 42.14) = 71.72, η_p_^2^ = .77, *p* < .001, and a significant condition x political orientation interaction effect, *F*(3, 63) = 33.87, η_p_^2^ = .62, *p* < .001. The main effect of political orientation was not significant, *F*(1, 21) = 2.75, η_p_^2^ = .12, *p* = .112.
 Simple effects analysis revealed a significant effect of political orientation on contact willingness with the individual who believe but do not spread conspiracy theories, *F*(1, 21) = 41.68, η_p_^2^ = .67, *p* < .001. Pairwise comparisons revealed that the classification image of the individual who believe in conspiracy theories, generated from participants on the political right, was rated significantly more positive in terms of contact willingness than the classification image generated from those on the political left (*d* = 1.97, *p* < .001). The moderating effect of participants’ political orientation was not significant in any of the other conditions (all *ps.* > .05).
